# Estradiol-Based Salicylaldehyde (Thio)Semicarbazones and Their Copper Complexes with Anticancer, Antibacterial and Antioxidant Activities

**DOI:** 10.3390/molecules28010054

**Published:** 2022-12-21

**Authors:** Tatsiana V. Petrasheuskaya, Ferenc Kovács, Nóra Igaz, Andrea Rónavári, Bálint Hajdu, Laura Bereczki, Nóra V. May, Gabriella Spengler, Béla Gyurcsik, Mónika Kiricsi, Éva Frank, Éva A. Enyedy

**Affiliations:** 1 Department of Inorganic and Analytical Chemistry, Interdisciplinary Excellence Centre, University of Szeged, Dóm tér 7, H-6720 Szeged, Hungary; 2 MTA-SZTE Lendület Functional Metal Complexes Research Group, University of Szeged, Dóm tér 7, H-6720 Szeged, Hungary; 3 Department of Organic Chemistry, University of Szeged, Dóm tér 8, H-6720 Szeged, Hungary; 4 Department of Biochemistry and Molecular Biology, University of Szeged, Közép fasor 52, H-6726 Szeged, Hungary; 5 Department of Applied and Environmental Chemistry, University of Szeged, Rerrich Béla tér 1, H-6720 Szeged, Hungary; 6 Institute of Materials and Environmental Chemistry, Research Centre for Natural Sciences, Magyar tudósok körútja 2, H-1117 Budapest, Hungary; 7 Centre for Structural Science, Research Centre for Natural Sciences, Magyar tudósok körútja 2, H-1117 Budapest, Hungary; 8 Department of Medical Microbiology, Albert Szent-Györgyi Health Center and Albert Szent-Györgyi Medical School, University of Szeged, Semmelweis u. 6, H-6725 Szeged, Hungary

**Keywords:** estradiol hybrids, (thio)semicarbazones, cytotoxicity, solution equilibrium, EPR spectroscopy, X-ray crystal structure

## Abstract

A series of novel estradiol-based salicylaldehyde (thio)semicarbazones ((T)SCs) bearing (O,N,S) and (O,N,O) donor sets and their Cu(II) complexes were developed and characterized in detail by ^1^H and ¹³C nuclear magnetic resonance spectroscopy, UV–visible and electron paramagnetic resonance spectroscopy, electrospray ionization mass spectrometry and elemental analysis. The structure of the Cu(II)-estradiol-semicarbazone complex was revealed by X-ray crystallography. Proton dissociation constants of the ligands and stability constants of the metal complexes were determined in 30% (*v/v*) DMSO/H_2_O. Estradiol-(T)SCs form mono-ligand complexes with Cu(II) ions and exhibit high stability with the exception of estradiol-SC. The Cu(II) complexes of estradiol-TSC and its *N,N*-dimethyl derivative displayed the highest cytotoxicity among the tested compounds in MCF-7, MCF-7 KCR, DU-145, and A549 cancer cells. The complexes do not damage DNA according to both in vitro cell-free and cellular assays. All the Cu(II)-TSC complexes revealed significant activity against the Gram-positive *Staphylococcus aureus* bacteria strain. Estradiol-TSCs showed efficient antioxidant activity, which was decreased by complexation with Cu(II) ions. The exchange of estrone moiety to estradiol did not result in significant changes to physico-chemical and biological properties.

## 1. Introduction

(Thio)semicarbazones ((T)SCs) represent a class of organic compounds that have a broad range of pharmacological effects [1]. TSCs were found to be strong metal chelating agents due to their unique geometry, the placement and distance of sulfur and nitrogen atoms that allow for bidentate coordination of metal ions such as Fe(III), Fe(II), Zn(II), Cu(II) [2]. Some TSC derivatives were reported to be effective against medical conditions such as tuberculosis, leprosy, bacterial and viral infections, psoriasis, rheumatism, trypanosomiasis, coccidiosis and against malignant diseases [3]. 3-Aminopyridine-2-carboxaldehyde thiosemicarbazone (Triapine) is the most prominent representative compound of α-*N*-heterocyclic TSCs. Triapine is a potent ribonucleotide reductase (RNR) inhibitor, and it has already been involved in ~30 phase I–III clinical trials for the treatment of mainly gynecologic and hematologic malignancies [4]. Notably, two other TSCs, namely, di-2-pyridylketone 4-cyclohexyl-4-methyl-3-thiosemicarbazone (DpC) [5] and 4-(2-pyridinyl)-2-(6,7-dihydro-8(5H)-quinolinylidene)-hydrazide (COTI-2), are currently undergoing a phase I evaluation as anticancer agents as well [6]. Another prominent class is the salicylaldehyde TSCs since, among them, many derivatives possess remarkable in vitro cytotoxic activity [7,8]. However, this type of compound generally exhibits lower cytotoxic activity in human cancer cells in comparison to α-*N*-pyridyl TSCs. Usually, complexation with certain metal ions may increase cytotoxic effects, especially with Cu(II) due to the redox properties of the complexes linked to the anticancer activity [9,10,11]. Additionally, anticancer activity can be tuned by the modification of the chemical profile of the lead compound [12].

In the last decades, compounds with steroidal scaffolds have been in focus not only due to their fascinating structural framework but also due to their astonishing array of biological properties [13]. It should be noted that sterane-based compounds have an excellent ability to penetrate cell membranes and bind to the nuclear and membrane receptors. Even a small change in the sterane moiety can bring out a comprehensive biological response [14]. Many anticancer sterane derivatives and their Cu(II), Ru(II), Pt(II) Ni(II), Fe(II) and Zn(II) complexes have been recently synthesized [15,16,17,18,19,20,21]. Recently, we also developed a number of tridentate estrone-(T)SC hybrid molecules and their Cu(II) complexes with promising anticancer activity [22,23] but with limited water solubility. They showed anticancer activity against the hormone-responsive MCF-7 breast cancer cell line (estrone-TSC: IC_50_ = 6.4 μM) and HCT-116 colon cancer cell line (Me-estrone-TSC and Me_2_-estrone-TSC: IC_50_ = 25 μM and 21 μM, respectively) [8,22,23]. However, their Cu(II) complexes displayed stronger cytotoxicity than the ligands for a series of human cancer cell lines (IC_50_ = 0.59–41 μM).

In this work, we developed four new (thio)semicarbazones based on an estradiol scaffold with a hydroxyl group at C-17 on ring D instead of a carbonyl moiety of the estrone-based ligands, which was expected to increase the hydrophilic nature of the new compounds. This modification was indicated by a four-times higher aqueous solubility of estradiol than estrone (*S* = 14.3 µM and 2.96 µM, respectively) [24]. The Cu(II) complexes were also synthesized and characterized by electron paramagnetic resonance (EPR), UV–visible (UV–vis) spectrophotometry, electrospray ionization mass spectrometry (ESI-MS), elemental analysis and single crystal X-ray diffraction. Their solution behavior and stability were investigated using UV–vis titrations. Due to the importance of the redox behavior of the Cu(II)-TSC complexes in their biological activity, the redox properties were monitored by cyclic voltammetry together with spectroelectrochemical studies. Radical scavenging activity and DNA cleavage ability were also tested. Finally, the anticancer and antimicrobial activities of the estradiol derivatives and their Cu(II) complexes were assayed against the hormone-responsive MCF-7 breast cancer cell line and the multidrug-resistant MCF-7/KCR cells and various Gram-positive and negative bacterial strains.

## 2. Results and Discussion

### 2.1. Synthesis, Proton Dissociation Processes and Fluorescence of (Thio)Semicarbazones of Estradiol Hybrids

**Scheme 1:**
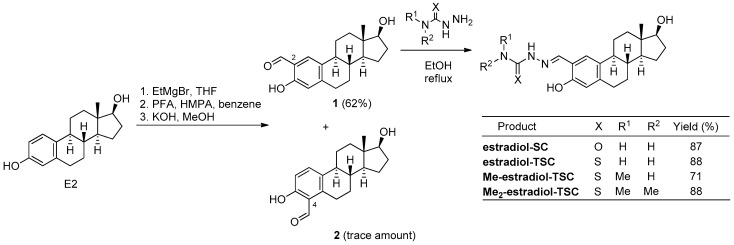
Synthesis of 2-formyl-estradiol and its condensation reactions with (thio)semicarbazides (PFA = paraformaldehyde, THF = tetrahydrofuran, HMPA = hexamethylphosphoramide, EtMgBr = ethylmagnesium bromide, EtOH = ethanol).

Regioselective ortho-formylation of estradiol, obtained from estrone by simple reduction, was performed according to methods in the literature [25,26] (Scheme 1). The regioselectivity for the formation of the 2-formyl- (**1**) over the 4-formyl isomer (**2**) was found to be better (isomeric ratio: 12:1) than in a similar reaction of estrone previously described [8]; therefore, the desired compound (**1**) could be purified by recrystallization from hot isopropyl alcohol.

Next, 2-formyl-estradiol (**1**) was reacted with an equivalent amount of semicarbazide (X = O, R^1^ = R^2^ = H), thiosemicarbazide (X = S, R^1^ = R^2^ = H), 4-methyl-3-thiosemicarbazide (X = S, R^1^ = Me, R^2^ = H) or 4,4-dimethyl-3-thiosemicarbazide (X = S, R^1^ = R^2^ = Me), respectively, in refluxing ethanol (EtOH) without using any catalyst. Since semicarbazide was used as its HCl salt, sodium acetate (NaOAc) was required as a base to release the reagent. The transformations were monitored by thin-layer chromatography (TLC) until complete conversions were achieved. After purification of the crude products by column chromatography, the corresponding estradiol-SC, estradiol-TSC, Me-estradiol-TSC and Me_2_-estradiol-TSC (Scheme 1) were obtained as (*E*) configurational isomers in good yields. The structures of all synthesized compounds were confirmed by ^1^H, ^13^C NMR spectroscopy and ESI–MS measurements.

**Figure 1:**
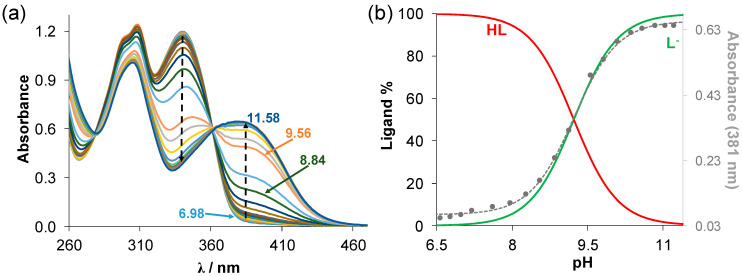
(**a**) UV–vis absorption spectra of estradiol-TSC recorded at various pH values in 30% (*v/v*) DMSO/H_2_O solvent mixture (c_ligand_ = 20 μM; *T* = 25.0 °C; *I* = 0.1 M (KCl); *ℓ* = 2 cm); (**b**) concentration distribution curves for this ligand with absorbance values measured at 381 nm (●) with the fitted curve. HL denotes the neutral form of the compound.

The title compounds (Scheme 1) were expected to have better solubility than their estrone congeners; however, their aqueous solubility was still too low for a pH-potentiometric study of their protonation equilibria. Therefore, these estradiol derivatives were investigated by UV–vis spectrophotometry at low ligand concentrations (20 µM) in 30% (*v/v*) dimethyl sulfoxide (DMSO)/H_2_O solvent mixture, similar to our former reports on estrone-(T)SCs [8,22] to obtain comparable data. Representative UV–vis spectra recorded for estradiol-TSC and estradiol-SC in the pH range (2.0–12.5) are shown in Figure 1a and Figure S1a, respectively.

No spectral changes were observed at pH < 7.5 (Figure 1a and Figure S1a), and the appearance of well-defined isosbestic points indicates that two species (HL, L^−^) are involved in the equilibrium process (Figure 1b and Figure S1b). The development of a new strong band with a higher wavelength of maximum absorbance was observed (estradiol-TSC: λ_max_ = 381 nm, Me-estradiol-TSC: λ_max_ = 384 nm, Me_2_-estradiol-TSC: λ_max_ = 380 nm, estradiol-SC: λ_max_ = 374 nm) most probably due to the deprotonation of the phenolic-OH moiety [8]. These compounds have two more proton dissociable groups, namely the hydrazonic-NH of the (T)SC moiety and the OH group of the D-ring. Their deprotonation might take place at a pH higher than 12.5, which is outside of our measured pH range. p*K*_a_ values (Table 1) and the spectra of the individual ligand species were calculated on the basis of deconvolution of recorded UV–vis spectra (Figure S2).

The estradiol-TSCs have similar p*K*_a_ values, and the monomethyl- and dimethyl-substitution has only a minor impact on the acidity of the compounds. On the other hand, the estradiol-SC has a higher p*K*_a_ by ca. half logarithmic unit than these thiosemicarbazones as it was also observed for the salicylaldehyde TSC–SC ligand pair (p*K*_a_ STSC = 8.84 [27], p*K*_a_ SSC = 9.30 [28]). Based on these p*K*_a_ values, it can be concluded that the studied compounds are present mostly in their neutral HL form at physiological pH. The obtained p*K*_a_ values are somewhat higher than those of the analogous estrone-(T)SCs (Table 1) [8,22,23]. These compounds were found to be more fluorescent than the corresponding estrone-(T)SCs at pH 7.4 in H_2_O (1% (*v*/*v*) DMSO/H_2_O) emitting light in the 350−550 nm wavelength range (Figure S3) [8,22,23].

### 2.2. Complex Formation Equilibria of Estradiol-(T)SCs with Cu(II) Ions and Their Structure

**Figure 2:**
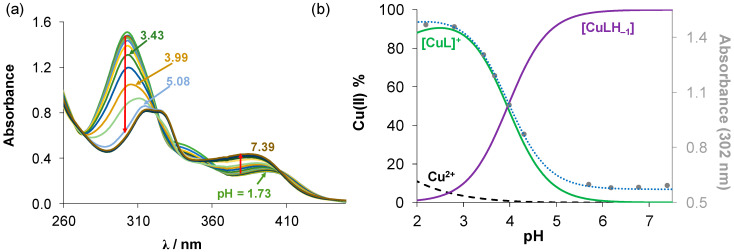
(**a**) UV–vis absorption spectra of the Cu(II)-estradiol-TSC (1:1) system in the pH range 1.73–7.39 in 30% (*v/v*) DMSO/H_2_O solvent mixture. (**b**) Concentration distribution curves calculated for the same system plotted together with the absorbance changes at 302 nm (●) with the fitted curve (dotted line). (c_ligand_ = 20 µM; *c*_Cu_(II) = 20 µM; *T* = 25.0 °C; *I* = 0.1 M (KCl)).

In order to reveal the stability and speciation of the Cu(II) complexes of estradiol-(T)SC, solution equilibrium studies were performed by UV–vis spectrophotometric titrations in 30% (*v*/*v*) DMSO/H_2_O. The complex formation processes of the analogous estrone-(T)SCs have already been investigated in our previous reports [8,22,23]; herein, similar binding modes and speciation are expected based on the close similarity of the chelating moieties of the ligands. Representative UV–vis spectra recorded for Cu(II)-estradiol-TSC and Cu(II)-estradiol-SC (1:1) systems are presented in Figure 2a and Figure S4a. Variation in the pH values reveals characteristic spectral changes in the UV–vis spectra and shows a single process up to pH 6.8, which is typical for deprotonation of hydrazonic nitrogen resulting in the (O^−^, N, S^−^) binding mode [8,22]. Although fairly low ligand concentrations were used (20 μM), some precipitate was observed at pH > ~8 using both 1:1 and 1:2 metal-to-ligand ratios, which hindered the determination of the formation constants for species present in the basic pH range. It should be noted that there was no precipitate in the case of the estrone analogs under the same conditions suggesting a worse aqueous solubility of the complexes of the estradiol derivatives despite our expectations. At ligand excess, there was no indication of the formation of bis complexes.

**Scheme 2:**
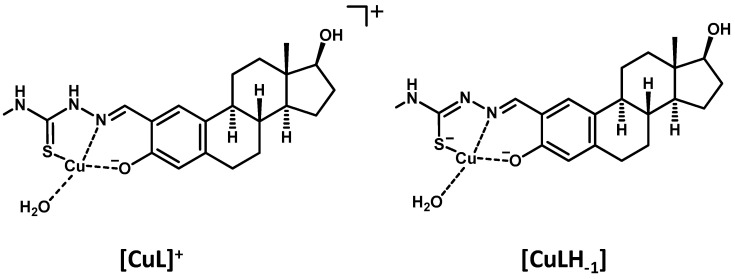
Suggested structures for the Cu(II) complexes formed with Me-estadiol-TSC.

Therefore, estradiol-(T)SCs form only mono-complexes [CuL]^+^ and [CuLH_−1_] at pH < 7.5. Complex [CuL]^+^ is formed in the acidic pH range, where the ligands coordinate in a tridentate mode via (O^−^,N,S)(H_2_O) for estradiol-TSCs and via the (O^−^,N,O)(H_2_O) donor set for estradiol-SC, respectively. Further deprotonation of hydrazonic nitrogen leads to the formation of complex [CuLH_−1_], which is the sole species between pH 5 and 7.4. The suggested structures for the complexes formed with estradiol-TSC are represented in Scheme 2. The deprotonation of the coordinated co-ligand water is feasible only at pH > 9 based on the p*K*_a_ values of [CuL]^+^ species determined for the estrone derivatives [8,22,23].

Overall stability constants for complexes [CuL]^+^ and [CuLH_−1_] were computed by the evaluation of the UV–vis spectra obtained at various pH values (Table 2, and for the individual molar absorbance spectra, see Figure S5). Additionally, concentration distribution curves (Figure 2b, Figure S4b) were also computed using the stability constants. In order to compare the stability of the complexes, pCu values were also calculated at pH 5 and at 20 μM concentrations of both the ligand and the metal ion using the overall stability constants (Table 2). pM values at pH 6.0 and 7.4 were also estimated for better interpretation of the obtained results, which will be further used in Section 2.6 (Table 2). pCu is the negative decadic logarithm of the unbound metal ion concentration under chosen conditions and provides a solid basis for comparison of the stability of the complexes. The higher pCu value shows a stronger metal binding ability of the ligand. These data reveal similar stability of the estradiol-(T)SC complexes compared to the corresponding estrone-(T)SC derivatives. The complexes of the *N*-dimethylated TSCs possess higher stability than the non-substituted or *N*-monomethyl derivatives as was also found for a series of α-*N*-pyridyl TSCs, including Triapine and its derivatives with methyl groups at different positions [29].

### 2.3. Synthesis, Characterization of Cu(II) Complexes of Estradiol–(T)SCs and Solid State Structural Studies of [Cu(HL)Cl_2_] × H_2_O × 2CH_3_OH

As the solution equilibrium studies indicated the formation of the Cu(II) complexes with high stability in solution, we attempted to isolate them. Therefore, the Cu(II) complexes were prepared by the reaction of CuCl_2_·2H_2_O with estradiol-(T)SC in a boiling mixture of methanol (MeOH)/H_2_O (at neutral pH), a procedure used previously for the synthesis of Cu(II) complexes with related TSCs [8,22]. The composition and purity of Cu(II) complexes were confirmed by ESI–MS, elemental analysis, UV–vis and EPR spectroscopy, and the isolated complexes were identified as the neutral [CuLH_−1_] with a water molecule as co-ligand. The positive ion ESI-MS spectra (see Figure S6) proved the 1:1 metal-to-ligand stoichiometry of the complex. Elemental analysis was performed for all four Cu(II) complexes confirming this stoichiometry. The UV–vis spectra were recorded in MeOH (Figure S7), which are fairly similar to each other except for the Cu(II)-estradiol-SC complex that has a (O^−^,N,O^−^) coordination mode instead of (O^−^,N,S^−^). Comparing these spectra to those of the free ligands, the complete complex formation could be concluded.

**Figure 3:**
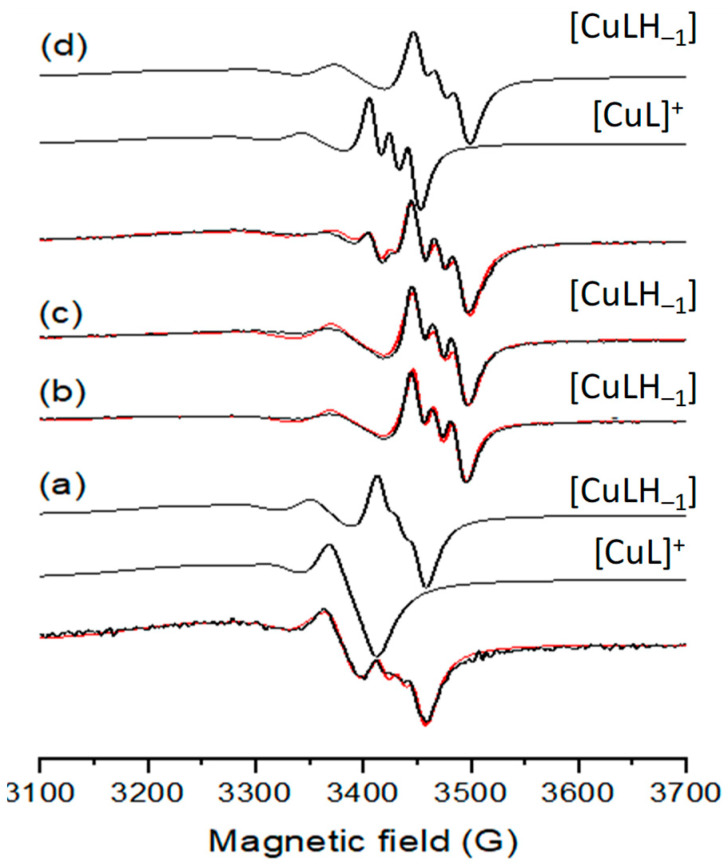
Room temperature solution EPR spectra (black) recorded for Cu(II) complexes dissolved in DMSO of (**a**) estradiol-SC, (**b**) estradiol-TSC, (**c**) Me-estradiol-TSC, (**d**) Me_2_-estradiol-TSC and simulated (red) spectra together with the obtained component spectra. Spectrum (**a**) was simulated with 50% [CuL]^+^ and 50% [CuLH_−1_], (**b**,**c**) with 100% [CuLH_−1_] and (**d**) with 18% [CuL]^+^ 82% [CuLH_−1_].

In order to confirm the coordination modes in the isolated Cu(II) complexes, EPR spectra were recorded at room temperature (isotropic spectra, Figure 3) and at 77 K (anisotropic spectra, Figure S8) for their DMSO solution. It should be noted that no free Cu(II) ions were detected. The isotropic and anisotropic EPR parameters (*g* and *A* values) were obtained by simulation of the EPR spectra and were collected in Table 3; Table 4. The isotropic values were compared to those of the Cu(II) complexes of estrone-(T)SC derivatives [8,22,23]. The estradiol-(T)SC complexes revealed similar isotropic EPR parameters; therefore, the same (O^−^,N,S^−^) and (O^−^,N,O^−^) coordination modes are suggested for estradiol-TSCs and estradiol-SC, respectively. It is worth mentioning that somewhat lower *g*_0_ values were obtained for the Cu(II) complexes of the TSCs than for the SC, showing a slightly higher ligand field in the TSC complexes.

Evaluation of the recorded room temperature and frozen solution EPR spectra (Figure 3 and Figure S8) reveals the presence of the [CuLH_−1_] species as major components. However, the fraction of [CuL]^+^ was found to be 18% for complex ofMe_2_-estradiol-TSC, and 50% for complex of estradiol-SC.

In order to obtain information about the major species at physiological pH, frozen solution EPR spectra in different biological media such as (2-hydroxyethyl)-1-piperazineethanesulfonic acid (HEPES) at pH 7.4, Eagle’s Minimum Essential Medium (EMEM), RPMI 1640 and human blood serum (HBS) were recorded (Figures S9 and S10) for selected compounds, namely for the complexes of Me_2_-estradiol-TSC and estradiol-SC. The determined anisotropic EPR parameters are shown in Table S1. Obtained EPR data were compared with those collected for the complexes dissolved in 20% (*v*/*v*) MeOH/DMSO and also with pure CuCl_2_ dissolved in the different biological medium (Figure S11) recorded at 77 K. The well-known spectra of Cu(II)(aqua) could only be detected in HBS as a minor species, which means that in all media, the complexing ligands in the medium bound the free copper. In all media, for the complex of Me_2_-estradiol-TSC, the presence of mixed ligand complexes was detected. The spectra in HBS and EMEM show a much stronger ligand field (lower *g*_z_ and higher *A*_z_ values) than was obtained for the original complex of Me_2_-estradiol-TSC. This is due to the coordination of another ligand at the vacant coordination sites around Cu(II) in this complex, which originates from the medium. In the case of the complex of estradiol-SC, the spectra in EMEM and RPMI 1640 showed the presence of CuCl_2_+EMEM spectra instead of the original complex; therefore, estradiol-SC was released from the coordination sphere of copper due to the complexing agents. Although, in HEPES and HBS, the recorded spectra were different from the spectra of both the CuCl_2_ and Cu(II)-estradiol-SC complex spectra. This suggests that in addition to the estradiol-SC, a second ligand from HEPES or HBS can coordinate to the Cu(II) ion. These findings also confirm the lower stability of the SC complex in comparison to the complex of the TSC.

**Figure 4:**
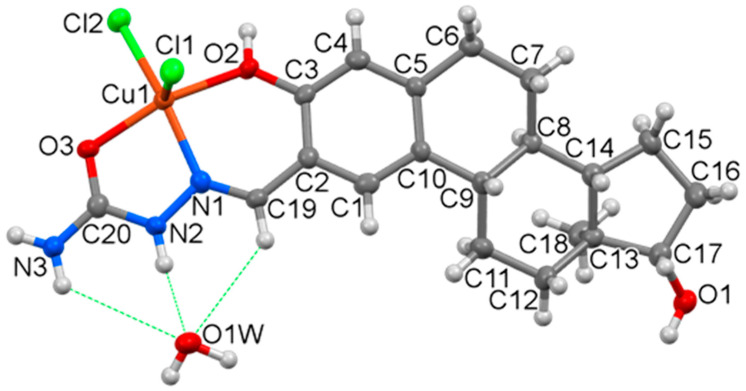
Molecular structure and atom labeling of the Cu(II) complex of estradiol-SC as the [Cu(HL)Cl_2_] × H_2_O × 2CH_3_OH complex molecule. Displacement parameters are drawn at the 50% probability level, and the methanol solvate molecules are omitted for clarity.

Furthermore, the coordination mode in the Cu(II)-estradiol-SC complex was determined by X-ray crystallography (Figure 4). Needle-like single crystals of Cu(II)-estradiol-SC complex were grown in MeOH (with some drops of HCl) by slow evaporation of the solvent. The complex [Cu(HL)Cl_2_] × H_2_O × 2CH_3_OH was crystallized in the orthorhombic crystal system in the *P*2_1_2_1_2_1_ space group. The unit cell contains four complex molecules. One complex molecule can be found in the asymmetric unit together with two methanol and one water molecule. The water is bound by three hydrogen bonds to the semicarbazide part of the ligand. The complex forms a square pyramidal system where estradiol-SC coordinates to the Cu(II) ion via the (O,N,O) donor set, and two coordinated chloride ions neutralize the charge of the central ion (Figure 4, Tables S2–S5). It should be noted that in this complex, the overall charge of the ligand is zero. The positions of the labile protons were determined on the basis of Fourier difference maps and in accordance with the hydrogen bond system of the compound. The configuration of the ligand is the same as for natural estradiol.

**Figure 5:**
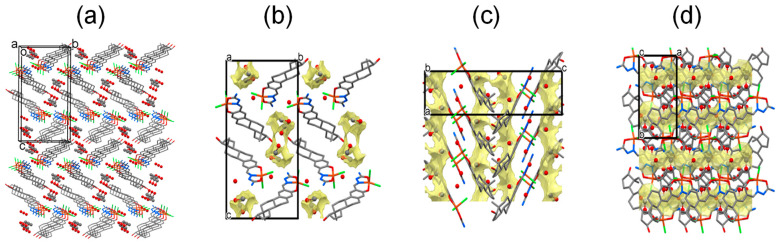
(**a**) Crystal packing of [Cu(HL)Cl_2_] × H_2_O × 2CH_3_OH complex of estradiol-SC drawn by stick representation and solvate molecules by ball and stick representation (hydrogen atoms are omitted for clarity). (**b**) View from the *a* crystallographic axis direction, methanol channels are marked with pale yellow, (**c**) view from the *b* crystallographic axis direction, (**d**) view from the *c* crystallographic axis direction.

The complex molecules are arranged parallel to each other in columns running along the *a* crystallographic axis (crystal packing is shown in Figure 5). MeOH solvate molecules are placed in channels running along the *a* crystallographic axis.

### 2.4. Redox Properties of the Cu(II) Complexes of Estradiol-(T)SCs

**Figure 6:**
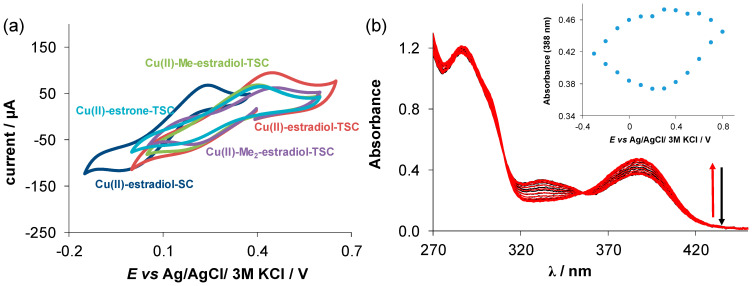
(**a**) Cyclic voltammograms of the Cu(II)-(T)SC (1:1) chemical systems in 90% (*v/v*) DMSO/H_2_O at 10 mV/s scan rate (*c*_ligand_ =1 mM, *c*_Cu(II)_ = 1 mM, pH = 7.40 (10 mM HEPES), *T* = 25 °C, *I* = 0.1 M (nBu_4_NPF_6_)) *E*’ vs. Ag/AgCl/3M KCl (V): Cu(II)–estradiol-SC = +0.10; Cu(II)-estrone-TSC = +0.28; Cu(II)-estradiol-TSC = +0.31; Cu(II)Me–estradiol-TSC = +0.29; Cu(II)-Me_2_-estradiol-TSC = +0.33; (**b**) UV–vis absorption spectra recorded for the Cu(II)-estradiol-SC system at the various potential values using the spectroelectrochemical cell. Inserted figure shows the absorbance values at 388 nm plotted against the potential. (*c_Cu_*_(II)_ = 100 µM; *ℓ* = 1 cm; *I* = 0.1 M (NBu_4_PF_6_); *T* = 25.0 °C, scan rate: 10 mV/s).

The redox properties of metal complexes offer unusual routes for new mechanisms of anticancer therapy. It can introduce artificial reductive and/or oxidative stress into cancer cells. Redox reactions in the reducing environment of cancer cells can activate metal complexes, and bioactive compounds/complexes can modulate the redox state of cancer cells [30]. For a deeper understanding of the redox behavior of Cu(II)-estradiol-(T)SC complexes, cyclic voltammetry measurements (Figure 6) were taken as a starting point. The electrochemical assays were performed in a 90% (*v*/*v*) DMSO/aqueous buffer (pH 7.4, 10 mM HEPES) solvent mixture with tetrabutylammonium hexafluorophosphate (nBu_4_NPF_6_) as a background electrolyte.

The voltammograms showed a redox activity in both cathodic and anodic regions (Figure 6a), where one-electron transfer quasi-reversible process was observed. This process corresponds to the reduction of Cu(II) to Cu(I). The formal potentials of the complexes were in the +0.10 to +0.33 V (vs. Ag/AgCl/3M KCl) potential range, giving the following order: estradiol-SC < estrone-TSC, Me-estradiol-TSC, estradiol-TSC, Me_2_-estradiol-TSC, indicating the slightly stronger oxidizing power of the TSC complexes. It should be noted that the large peak-to-peak separation in the corresponding voltammogram can be attributed to the significant structural differences between the complexes formed with Cu(II) and Cu(I) [7].

To further explore the chemical changes occurring during this, one electron transfer process in situ UV–vis spectroelectrochemical measurements together with cyclic voltammetry were carried out in a special thin layer spectroelectrochemical cell with a microstructured honeycomb working electrode. The UV–vis spectra measured upon cathodic reduction revealed decreasing absorbance at 388 nm, as well as a decrease in the absorbance bands at 286 and 330 nm (Figure 6b). Upon the reversal scan, the product that was formed upon the reduction of Cu(II) to Cu(I) was re-oxidized to the initial state (Figure 6b insert). This finding unambiguously indicates that Cu(I) species are most probably stable without dissociation of the metal complex. The same behavior was observed for all tested TSCs.

In addition to the spectroelectrochemical and cyclic voltammetric experiments, the direct redox reaction of the Cu(II) complexes with physiological reductants, such as glutathione (GSH) and ascorbic acid (AA), was also investigated in order to check their reactivity. It was previously reported that GSH was able to reduce Cu(II) complexes of α-*N*-pyridyl TSCs resulting in the formation of Cu(I) species, while re-oxidation with oxygen could generate reactive oxygen species (ROS) [29]. In this work, the direct reactions were followed by using UV–vis spectrophotometry in a tandem cuvette under strictly anaerobic conditions in 30% (*v*/*v*) DMSO/H_2_O solvent mixture at pH 7.4. The spectral changes were monitored in the wavelength range of 250–480 nm using a high excess of the reducing agent (50 equiv.). In this wavelength range, the spectral changes are characteristic only of the absorption of the Cu(II)-TSC complex and the ligand.

**Figure 7:**
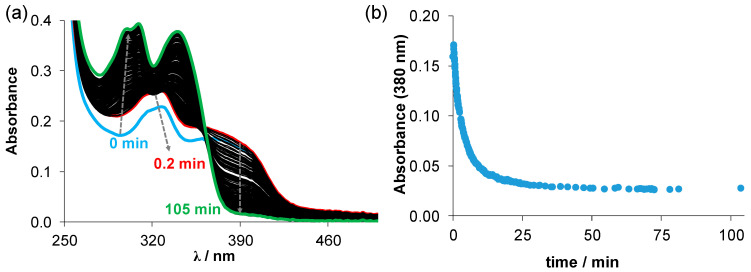
(**a**) Time-dependent changes of the UV–vis spectra recorded for the Cu-Me-estradiol-TSC complex (25 µM) in the presence of 50 equiv. GSH (1.25 mM) at pH 7.4 (10 mM HEPES) in 30% (*v/v*) DMSO/H_2_O under anaerobic conditions. (**b**) Absorbance values at 380 nm plotted against the time. (*T* = 25 °C; *I* = 0.1 M (KCl)).

An example is shown for the reaction of complex Cu(II)-Me-estradiol-TSC with GSH (Figure 7a), where the recorded spectra reveal that after mixing the reactants, a significant spectral change is observed (comparing spectra at 0 and 0.2 min), most probably as a result of the formation of a mixed ligand complex with GSH as it was reported for other TSCs [8,30]. Afterward, a significant decrease in the absorbance of the S→Cu CT band at λ_max_ 380 nm was observed, while the absorbance was increased at the λ_max_ of the free ligand (~308 and 340 nm). These changes indicate the liberation of the TSC ligand, while Cu(I) most probably forms a complex with GSH that is present in a high excess in the solution. Bubbling oxygen into the solution could regenerate the complex (Figure S12). This finding strongly suggests the reversibility of the redox process, which was also observed in the cyclic voltammetric experiments. The other Cu(II) complexes showed similar behavior, except for the Cu(II)-estradiol-SC complex, where a rapid reduction of Cu(II) was noticed. Practically, the first spectrum recorded after mixing the reactants suggested the disappearance of the Cu(II) complex and the presence of the unbound semicarbazone. It should be noted that the reaction was not fully reversible upon bubbling O_2_ in the sample.

From the measured absorbance−time curves, observed rate constants (*k_obs_*) were calculated (Table 2). The *k_obs_* values somewhat differ; namely, the following trend was found: estradiol-TSC < Me-estradiol-TSC < Me_2_-estradiol-TSC, which interestingly follows the stability order of the complexes.

Additionally, the reaction with ascorbic acid was also tested, but the process was very slow in the case of estradiol-TSCs, suggesting that the studied Cu(II) complexes cannot be reduced efficiently by this weaker reducing agent. On the contrary, remarkable spectral changes were detected for the Cu(II)–estradiol-SC system (Figure S13). After mixing the reactants, the formation of a mixed-ligand complex is assumed, similarly to what was found for the Cu(II)-estradiol-TSC complexes upon their reaction with GSH above. A shift in the absorbance bands is seen due to the possible monodentate coordination of the ascorbate to the Cu(II)-estradiol-SC complex. Afterward, a similar explanation for this redox reaction process can be used as was explained for the redox reaction of GSH with estradiol-TSCs.

Taking these results into account, it can be concluded that all tested compounds are redox-active, which is most probably connected to their antioxidant capacities, which were also investigated and presented in the next chapter.

### 2.5. Free Radical Scavenging Activity of Estradiol-(T)SC

**Figure 8:**
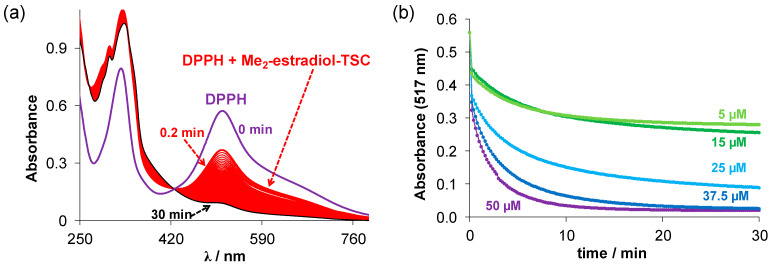
(**a**) Time-dependent changes of the UV–vis spectra of the DPPH• (*c* = 50 μM) in the presence of Me_2_-estradiol-TSC (50 µM) in ethanol. (*T* = 25 °C); (**b**) absorbance changes of DPPH at 517 nm upon reaction with Me_2_-estradiol-TSC at different concentrations in ethanol plotted against time. (*c_DPPH_* = 50 µM; *c_ligand_* = 50, 37.5; 25; 15; 5 µM; *ℓ* = 1 cm*; T* = 25 °C).

Estradiol-(T)SCs were tested for free radical scavenging activity through the 2,2-diphenyl-1-picrylhydrazyl (DPPH) assay. In addition, salicylaldehyde TSC (STSC) and estradiol were screened as structural models of estradiol-(T)SCs. Estrone-TSC was used to monitor the effect of the exchange of 17-ketone to 17-OH on the D-ring of the sterane scaffold. The direct reaction between DPPH and the investigated compounds at various concentrations was studied spectrophotometrically. UV–vis spectra of the mixtures were tracked over time till the redox equilibrium was reached. Representative UV–vis spectra for Me_2_-estradiol-TSC are shown in Figure 8a. Changes in absorbance at the λ_max_ of DPPH (517 nm) in time at the different DPPH-to-Me_2_-estradiol-TSC ratios are compared in Figure 8b. Free radical scavenging activities are usually indicated as IC_50_ values (the concentration at which the compound reduces 50% of the DPPH radicals present in the reaction mixture), and these values can be compared to that of the reference compound 6-hydroxy-2,5,7,8-tetramethylchroman-2-carboxylic acid (Trolox) [31]. The results of DPPH assay (IC_50_ and Trolox equivalent antioxidant capacity (TEAC) values) are collected in Table 5.

The lower IC_50_ and the higher TEAC values mean a stronger antioxidant effect. Based on the obtained data, Me_2_-estradiol-TSC showed the potential to act as an antioxidant agent among other (thio)semicarbazones. It should be noted that the presence of Cu(II) ions does not increase the antioxidant activity and even decreases it. Considering that, in this reaction, DPPH is reduced by the partner, the copper complex is assumed to possess an oxidizing (*vide supra* their reaction with GSH) rather than a reducing effect.

### 2.6. In Vitro DNA Cleavage

**Figure 9:**
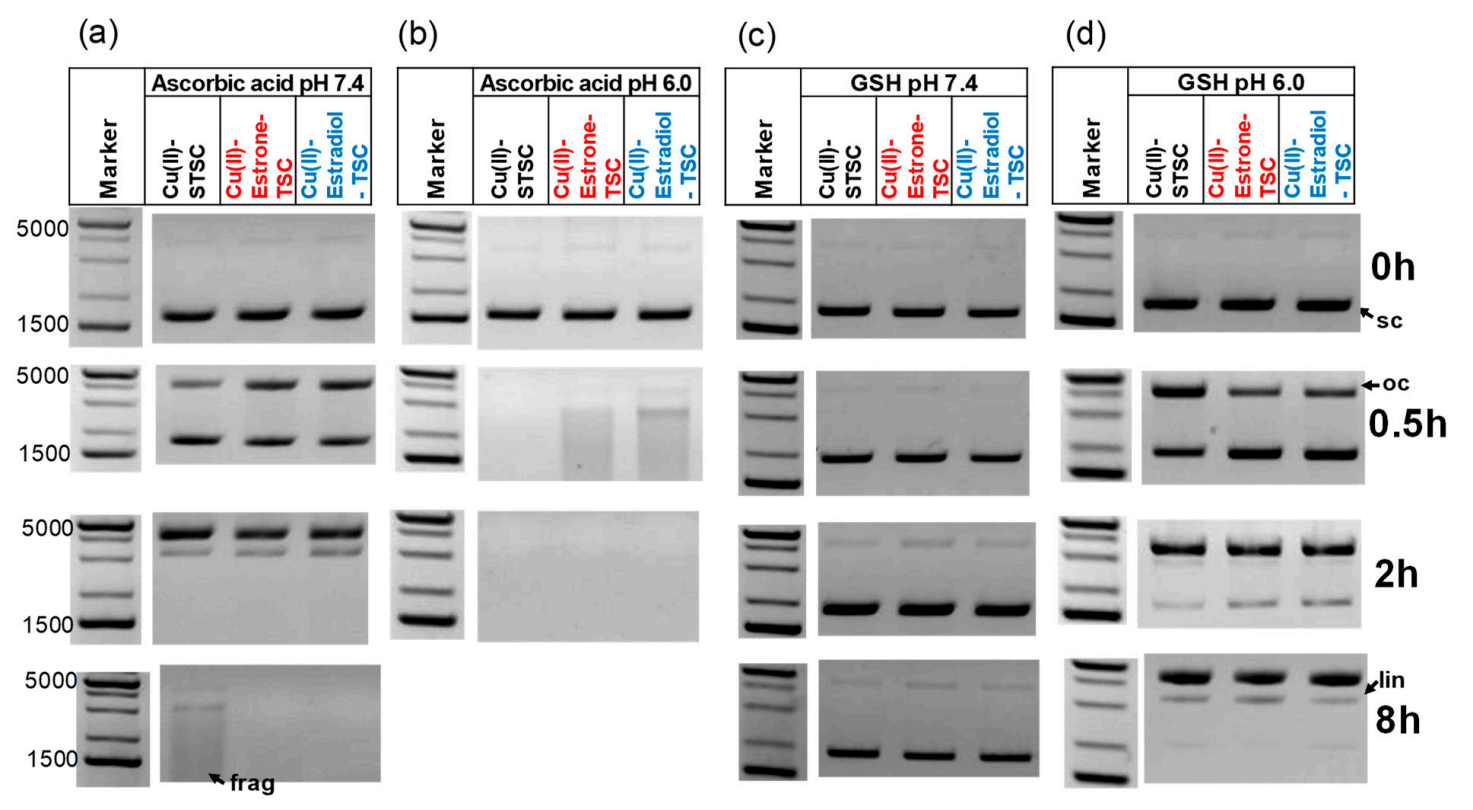
Representative DNA cleavage activity of the Cu(II)-TSC complexes analyzed by 1% (*w*/*v*) agarose gel electrophoresis in the presence of 1 mM ascorbic acid (**a**) at pH 7.4 and (**b**) at pH 6; or in the presence of 1 mM GSH (**c**) at pH 7.4 and (**d**) at pH 6.0. The reaction mixtures contained 100 ng pUC119ΔH+N-N6 plasmid DNA. (*c*_L_ = 20 μM; *c*_Cu_(II) = 18 μM; (10 mM HEPES); incubation time at 37 °C: 0, 0.5, 2 and 8 h). Abbreviations indicate various forms of the plasmid DNA: oc—open circular, lin—linear, sc—supercoiled frag—fragmented. 1 kb Plus DNA. Ladder was used as a marker.

Since redox active Cu(II) complexes may result in DNA damage, the induced DNA-cleavage was studied by gel electrophoresis. It was found that none of the investigated ligands and their Cu(II) complexes had a relevant DNA damaging effect over the 24 h investigation period in the absence of any reducing agent (Figure S14). In the presence of ascorbic acid (~50 equiv.), the complexes exhibited nuclease activity (Figure 9 and Figure S15), which was higher than the effect of free Cu(II) released from the complexes based on their pCu value (Table 2, Figure S16) but lower compared to the Cu(II) ascorbic acid system containing equal concentration (18 μM) of Cu(II). At pH 7.4, the complexes completely fragmented the DNA within 8 h (Figure 9a), and by lowering the pH, the reactivity increased. At pH 6.0, 30 min were enough to complete DNA fragmentation (Figure 9b). In contrast, GSH did not induce DNA cleavage of the complexes at pH 7.4 (Figure 9c and Figure S17). At this pH, the Cu(II)-GSH system is almost inactive (Figure S17). However, significant acceleration of the latter reaction was observed by lowering the pH to 6.0 (Figure 9d and Figure S17). The DNA-damaging effect related to the complexes at pH 6.0 was comparable to that of the Cu(II)-GSH system at the early stage of the reaction. While GSH reduces the Cu(II) complexes rapidly at pH 7.4 (see Section 2.4.), causing the complete decomposition of the complex after ~30 min (Figure 7), the DNA damage of the complexes at pH = 6.0 slowed down in comparison to the Cu(II)-GSH control measurement. This finding may indicate that the reaction occurs without dissociation of the TSC complexes, in agreement with the finding published recently by Faller et al. [30].

### 2.7. Antibacterial Activity of the Ligands and the Effect of Complexation with Cu(II)

The antibacterial activity of estradiols-(T)SCs was studied on the Gram-positive *Staphylococcus aureus*, *Enterococcus faecalis* and the Gram-negative *Escherichia coli* and *Klebsiella pneumoniae* bacterial strains. Estrone-TSC and CuCl_2_ were also tested for comparison. Moreover, the activity was determined for the compounds in the presence of 1 equiv. CuCl_2_. The minimum inhibitory concentration (MIC) value is the lowest concentration of a compound that inhibits the growth of the bacteria. The obtained MIC values are presented in Table 6. Neither ligands nor Cu(II) complexes had activity on the Gram-negative bacterial strains *E. coli* and *K. pneumoniae* (MIC >100 μM). Estradiol-SC and estradiol-TSC showed some activity on the Gram-positive *S. aureus* bacterial strain (50 and 25 μM, respectively). Complexation with Cu(II) did not induce an antibacterial effect in the case of the semicarbazone, while the Cu(II) complexes formed with the thiosemicarbazones displayed a marked antibacterial effect. Cu(II) complexes of estradiol-TSC had a potent activity on Gram-positive bacteria, especially on the *S. aureus* strain (MIC: 3.125 μM for Cu(II)-estradiol-TSC, 6.25 μM for Cu(II)-Me-estradiol-TSC and 0.78 μM for Cu(II)-Me_2_-estradiol-TSC complexes). Furthermore, a moderate activity of the Cu(II) complexes of the TSCs was found on the Gram-positive *E. faecalis* strain.

### 2.8. Cytotoxicity of Cu(II) Complexes on Various Cancer Cells

The effect of Cu(II) complexes and the individual ligands (the estradiol-derivatives: estradiol-TSC, Me-estradiol-TSC, Me_2_-estradiol-TSC, and estrone-TSC for comparison) on the viability of cancer cells was tested on MCF-7, MCF-7 KCR, DU-145, and A549 cells first by MTT assays, which reflect the metabolic performance of living cells following treatments. No toxic effect was detected in the vehicle control (DMSO treated) and in the CuCl_2_-treated samples (Figures S18–S21). According to our results, neither the estradiol-SC nor the Cu(II)-estradiol-SC complex exhibit any harmful effect on MCF-7 or DU-145 cells and were only slightly toxic to A549 and MCF-7 KCR cell lines. Nevertheless, there was no difference in the cytotoxicity of the ligand and its copper complex (Figures S18–S21). On the other hand, although the tested TSCs were generally not toxic to cancerous cells or resulted in only a minor decrease in cell viability, their Cu(II) complexes were highly effective and caused massive viability loss compared to the control samples and the individual ligands. A significant difference was observed between the toxicity of estradiol-TSC and Cu(II)-estradiol-TSC, as well as between estrone-TSC and Cu(II)-estrone-TSC applied in each concentration on MCF-7 cells, where these Cu(II) complexes were more effective in each case (Figure S18). In DU-145 and MCF-7 KCR cells, the tested Cu(II) complexes of the TSCs significantly reduced cell viability more than the individual ligands. Based on the MTT results, the IC_50_ values of every treatment were calculated (Table 7). Among all the Cu(II) complexes and their ligands, Cu(II)-estrone-TSC shows the lowest IC_50_ concentration (4.28 µM) on MCF-7, (12.75 µM) on MCF-7 KCR cells and proved to be highly toxic to DU-145 (11.37 µM) and A549 (4.03 µM) cells. Similarly, the performance of Cu(II)-estradiol-TSC was also remarkable, mostly on MCF-7 (IC_50_ = 8.40 µM), MCF-7 KCR (12.95 µM) and on A549 (IC_50_ = 10.03 µM); nevertheless, on DU-145 and A549, Cu(II)-Me_2_-estradiol-TSC was the most effective complex. None of the respective ligands caused a significant reduction in the viability of the cancer cells, except for a slightly toxic effect on A549 cells. These results prove the significant enhancement of the anticancer performance of the tested estrogen derivatives when these are applied as Cu(II) complexes.

**Figure 10:**
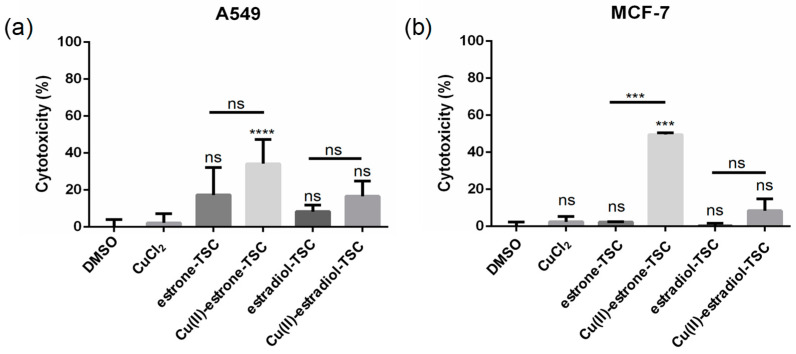
LDH assay to measure the reduction in plasma membrane integrity: cytotoxicity (%) obtained on (**a**) A549 and (**b**) MCF-7 cells upon the treatment with estrone-TSC and estradiol-TSC and their Cu(II) complexes (20 μM) in addition to the DMSO blank (90% DMSO and 10% phosphate buffered saline (PBS)) and CuCl_2_ (20 μM), as indicated in the figure using 24 h incubation time. (ns: not-significant; ***: *p* < 0.005; ****: *p* < 0.001).

Based on the MTT cell viability tests, for further investigations, the estrone-TSC, Cu(II)-estrone-TSC, estradiol-TSC, and Cu(II)-estradiol-TSC complexes were selected. To verify the cytotoxic effect of the chosen compounds, lactate dehydrogenase (LDH) assay was performed, where the activity of LDH released from damaged cells was assessed. In MCF-7 KCR and DU-145 cells, no differences in the released LDH activity were observed upon the treatments compared to the DMSO-treated control samples (Figure S22). However, in MCF-7 and in A549 cells, Cu(II)-estrone-TSC caused significant damage to the plasma membrane resulting in increased LDH activity and, thus, cytotoxicity compared to the control cells (Figure 10). These results further validated the strong anticancer potential of the Cu(II)-estrone-TSC complex.

**Figure 11:**
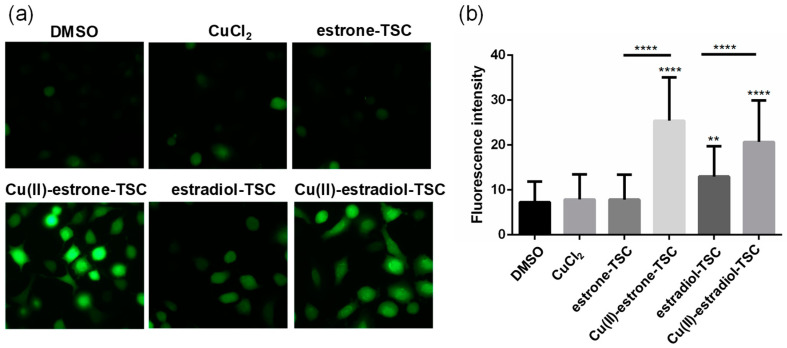
(**a**) Representative fluorescence microscopic images show the DCFDA staining to determine ROS levels in A549 cells upon the treatment with estrone-TSC and estradiol-TSC and their Cu(II) complexes (20 μM) in addition to the DMSO blank (90% DMSO and 10% PBS) and CuCl_2_ (20 μM) as indicated in the figure using 6 h incubation time. (**b**) Level of ROS generation measured by DCFDA staining on A549 cells. (**: *p* < 0.01; ****: *p* < 0.001).

Frequently, behind a reduced metabolic performance and a strong plasma membrane, damage of cells leading to cytotoxicity results in the formation of large amounts of ROS. To test whether oxidative stress and ROS generation are the causes behind the anticancer effects of the selected Cu(II) complexes, dichlorodihydrofluorescein diacetate (DCFDA) staining was performed (Figure 11 (for A549), S23 and S24 (for MCF-7, DU-145 and MCF-7 KCR)). The intracellular amount of ROS increased significantly upon treatments with the Cu(II)-estrone-TSC complex in every tested cell line compared to the DMSO-treated control samples. Moreover, in A549 (Figure 11b), MCF-7 and DU-145 cells, significantly higher fluorescence intensity was measured upon treatment with the Cu(II)-estrone-TSC complex than in estrone-TSC-treated samples, proving that this copper complex is more potent in triggering ROS production than the ligand itself. Cu(II)-estradiol-TSC significantly increased the intracellular level of reactive oxygen species in A549 (Figure 11b), DU-145 and MCF-7 KCR cells compared to the control; moreover, the fluorescence intensity of Cu(II)-estradiol-TSC exposed cells was significantly higher than those of the ligand-treated ones, indicating a larger ROS formation as a result of the treatment with the Cu(II) complex than the estradiol-TSC.

Since ROS formation might lead to damage of the DNA, we performed *γ*H2AX staining on these cancer cells following exposures to estrone-TSC, estradiol-TSC, complexes Cu(II)-estrone-TSC and Cu(II)-estradiol-TSC, DMSO, and CuCl_2_ solution to detect and quantify the amount of DNA double-strand breaks. None of these treatments caused a higher degree of DNA damage than what was observed in untreated control cells, indicating that the extent of ROS formation was probably not sufficient to be detrimental to DNA (Figure S25).

## 3. Materials and Methods

### 3.1. Chemicals

KCl, KOH, HCl, GSH, EDTA, DMSO were obtained from VWR (Hungary), and ascorbic acid, NaH_2_PO_4_, KH_2_PO_4_, HEPES were purchased from Sigma-Aldrich and used without further purification. Milli-Q water was used for sample preparation. CuCl_2_ stock solution was made by the dissolution of anhydrous CuCl_2_ in water, and its exact concentration was determined by complexometry through the EDTA complex. The stock solution of the ligands was prepared on a weight-in-volume basis dissolved in DMSO.

Reagents and materials used for the synthesis were purchased from commercial suppliers (TCI, Tokyo, Japan; Alfa Aesar, Haverhill, MA, USA and Sigma-Aldrich Corporation, St. Louis, MO, USA) and used as received. All solvents were dried and purified according to standard procedures. Thin layer chromatography was carried out on Kieselgel-G (Si 254 F, Merck KGaA, Darmstadt, Germany) plates (0.25 mm thick). The spots were detected by spraying with phosphomolybdic acid (5%) in aqueous phosphoric acid (50%) or visualized by UV light 254 nm. The products were purified by column chromatography on Merck silica gel 60, 40–63 μm (Merck KGaA, Darmstadt, Germany). Melting points (Mps) were measured on an SRS Optimelt digital device (Stanford Research Systems Inc, Sunnyvale, CA, USA). NMR spectra were recorded at 298 K with a Bruker DRX 500 instrument (Bruker, Billerica, MA, USA). Chemical shifts are reported in ppm (δ scale) and coupling constants (J) in Hz. The ^1^H resonance signals are indicated as a singlet (s), a broad singlet (bs), a doublet (d), a double doublet (dd), a triplet (t) or a multiplet (m). The ^13^C NMR spectra are ^1^H-decoupled. The J-MOD pulse sequence was applied to determine multiplicities.

### 3.2. Synthesis of TSC Derivatives (General Synthetic Method)

2-Formyl-estradiol (150 mg, 0.5 mmol) was suspended in abs. ethanol (EtOH) (5 mL), and then, semicarbazide hydrochloride (1.0 equiv.) and sodium acetate (NaOAc) (1.0 equiv.) or the appropriate thiosemicarbazide reagent (1.0 equiv.) were added. The mixture was stirred at 80 °C until complete conversion (TLC), and then the solvent was evaporated in vacuo. The crude product was purified by column chromatography with MeOH / ethyl acetate (EtOAc) (10:90% (*v/v*)) for estradiol-SC or MeOH/CH_2_Cl_2_ (2:98% (*v*/*v*)) for estradiol-TSC, Me-estradiol-TSC and Me_2_-estradiol-TSC as eluent.

#### 3.2.1. 2-[(3,17 Dihydroxy-estra-1,3,5(10)-triene-2-yl)methylene]Hydrazine-1-carboxamide (Estradiol-SC)

According to the General Synthetic Method, semicarbazide hydrochloride (55 mg) and NaOAc (41 mg) were used. Compound estradiol-SC was obtained as a white solid after purification (155 mg, 87%), Mp = 238 °C; ^1^H NMR (DMSO-*d_6_*, 500 MHz): δ 0.68 (3 H, s, 18-CH_3_), 1.06−1.44 (7 H, m), 1.59 (1 H, m), 1.78 (1 H, m), 1.88 (2 H, m), 2.07 (1 H, m), 2.39 (1 H, m), 2.73 (2 H, m), 3.53 (1 H, m, 17α-H), 4.41 (1 H, d, J 4.8, 17-OH), 6.28 (2 H, bs, NH_2_), 6.54 (1 H, s, 4-H), 7.51 (1 H, s, 1-H), 8.09 (1 H, s, HC=N), 9.66 (1 H, bs, NH), 10.01 (1 H, s, 3-OH); ^13^C NMR (DMSO-*d_6_*, 125 MHz): δ 11.1 (18-CH_3_), 22.7 (CH_2_), 25.9 (CH_2_), 26.6 (CH_2_), 29.0 (CH_2_), 29.8 (CH_2_), 36.5 (CH_2_), 38.5 (CH), 42.7 (13-C), 43.4 (CH), 49.5 (CH), 80.0 (17-CH), 115.5 (4-CH), 117.7 (2-C), 123.5 (1-CH), 131.2 (10-C), 138.8 (5-C), 138.9 (HC=N), 153.6 (3-C), 156.3 (C=O) (Figures S26 and S27). ESI-MS (methanol, positive): *m/z* 358.2123 [M + 1]^+^, 358.2130 calcd. for C_20_H_28_N_3_O_3_, 380.1945 [M + Na]^+^, 380.1945 calcd. for C_20_H_27_N_3_O_3_Na (Figure S28). λ_max_ (nm) in methanol: 284, 330 nm.

#### 3.2.2. 2-((3,17 Dihydroxy-estra-1,3,5(10)-triene-2-yl)methylene)Hydrazine-1-carbothioamide (Estradiol-TSC)

According to the General Synthetic Method, thiosemicarbazide (45 mg) was used. Compound estradiol-TSC was obtained as a white solid after purification (164 mg, 88%), Mp = 229−231 °C; ^1^H NMR (DMSO-*d_6_*, 500 MHz): δ 0.68 (3 H, s, 18-CH_3_), 1.07–1.44 (7 H, m), 1.59 (1 H, m), 1.74−1.82 (1 H, m), 1.83–1.94 (2 H, m), 2.07 (1 H, m), 2.44 (1 H, m), 2.73 (2 H, m), 3.53 (1 H, m, 17α-H), 4.41 (1 H, d, J 4.8, 17-OH), 6.55 (1 H, s, 4-H), 7.66 (1 H, s, 1-H), 7.84 (1 H, bs, one H of NH_2_), 7.95 (1 H, bs, the other H of NH_2_), 8.33 (1 H, s, (HC=N)), 9.45 (1 H, bs, NH), 11.20 (1 H, s, 3-OH); ^13^C NMR (DMSO-*d_6_*, 125 MHz): δ 11.1 (18-CH_3_), 22.7 (CH_2_), 25.8 (CH_2_), 26.6 (CH_2_), 29.1 (CH_2_), 29.8 (CH_2_), 36.5 (CH_2_), 38.5 (CH), 42.7 (13-C), 43.5 (CH), 49.5 (CH), 80.0 (17-CH), 115.6 (4-CH), 117.5 (2-C), 123.6 (1-CH), 131.4 (10-C), 140.0 (5-C), 140.9 (HC=N), 154.1 (3-C), 177.4 (C=S) (Figures S25 and S26). ESI-MS (methanol, positive): *m/z* 374.1892 [M + 1]^+^, 374.1902 calcd. for C_20_H_28_N_3_O_2_S, 396.1710 [M + Na]^+^, 396.1722 calcd. for C_20_H_27_N_3_O_2_SNa (Figure S27). λ_max_ (nm) in methanol: 310, 344 nm.

#### 3.2.3. 2-((3,17 β-Dihydroxy-estra-1,3,5(10)-triene-2-yl)Methylene)-N-methylhydrazine-1-carbothioamide (Me-estradiol-TSC)

According to the General Synthetic Method, 4-methyl-3-thiosemicarbazide (53 mg) was used. Compound Me-estradiol-TSC was obtained as a yellowish solid after purification (138 mg, 71%), Mp (decomp. > 180 °C); ^1^H NMR (DMSO-*d_6_*, 500 MHz): δ 0.68 (3 H, s, 18-CH_3_), 1.07–1.44 (7 H, m), 1.59 (1 H, m), 1.75–1.82 (1 H, m), 1.88 (2 H, m), 2.08 (1 H, m), 2.44 (1 H, m), 2.74 (2 H, m), 3.03 (3 H, d, J 4.5), 3.54 (1 H, m, 17α-H), 4.42 (1 H, d, J 4.8, 17-OH), 6.55 (1 H, s, 4-H), 7.65 (1 H, s, 1-H), 8.28 (1 H, q, J 4.5, 4.5, 4.7, NH-Me), 8.33 (1 H, s, HC=N), 9.46 (1 H, bs, NH), 11.23 (1 H, s, 3-OH); ^13^C NMR (DMSO-*d_6_*, 125 MHz): δ 11.2 (18-CH_3_), 22.7 (CH_2_), 25.9 (CH_2_), 26.6 (CH_2_), 29.1 (CH_2_), 29.8 (CH_2_), 30.8 (NH-Me), 36.5 (CH_2_), 38.5 (CH), 42.7 (13-C), 43.5 (CH), 49.5 (CH), 80.0 (17-CH), 115.6 (4-CH), 117.5 (2-C), 123.4 (1-CH), 131.3 (10-C), 140.0 (5-C), 140.6 (HC=N), 154.1 (3-C), 177.4 (C=S) (Figures S26 and S27). ESI-MS (methanol, positive): *m/z* 388.2052 [M + 1]^+^, 388.2058 calcd. for C_21_H_29_N_3_O_2_S (Figure S28). λ_max_ (nm) in methanol: 312, 344 nm.

#### 3.2.4. 2-((3,17 β-Dihydroxy-estra-1,3,5(10)-triene-2-yl)methylene)-N,N-dimethylhydrazine-1-carbothioamide (Me_2_-estradiol-TSC)

According to the General Synthetic Method, 4,4-dimethyl-3-thiosemicarbazide (60 mg) was used. Compound Me_2_-estradiol-TSC was obtained as a beige solid after purification (177 mg, 88%), Mp (decomp. > 220 °C); ^1^H NMR (DMSO-*d_6_*, 500 MHz): δ 0.68 (3 H, s, 18-CH_3_), 1.06–1.44 (7 H, m), 1.59 (1 H, m), 1.80 (1 H, m), 1.83–1.95 (2 H, m), 2.11 (1 H, m), 2.31 (1 H, m), 2.77 (2 H, m), 3.29 (6 H, s), 3.54 (1 H, m, 17α-H), 4.42 (1 H, d, J 4.9, 17-OH), 6.58 (1 H, s, 4-H), 7.20 (1 H, s, 1-H), 8.43 (1 H, s, HC=N), 11.10 (1 H, bs, NH), 11.39 (1 H, bs, 3-OH); ^13^C NMR (DMSO-*d_6_*, 125 MHz): δ 11.1 (18-CH_3_), 22.6 (CH_2_), 25.8 (CH_2_), 26.6 (CH_2_), 29.0 (CH_2_), 29.8 (CH_2_), 36.4 (CH_2_), 38.4 (CH), 40.8 (N-Me), 42.7 (13-C), 43.2 (CH), 49.5 (CH), 79.9 (17-CH), 115.9 (4-CH), 116.0 (2-C), 126.6 (1-CH), 130.9 (10-C), 139.7 (5-C), 147.0 (HC=N), 154.8 (3-C), 179.3 (C=S) (Figures S26 and S27). ESI-MS (methanol, positive): *m/z* 402.2210 [M + 1]^+^, 402.2215 calcd. for C_22_H_32_N_3_O_2_S, 424.2031 [M + Na]^+^, 424.2035 calcd. for C_22_H_31_N_3_O_2_SNa (Figure S28). λ_max_ (nm) in methanol: 292, 340 nm.

### 3.3. Synthesis and Characterization Cu(II)-TSC Complexes

The ligand estradiol-SC (0.12 mmol), estradiol-TSC (0.12 mmol), Me-estradiol-TSC (0.12 mmol) or Me_2_-estradiol-TSC (0.12 mmol) were dissolved in MeOH (10 mL), and then CuCl_2_ aqueous solution (0.98 mL, 0.12 mmol) was added. The pH was adjusted to pH ~ 7.4 by the addition of HEPES (10 mM, 5 mL). Green precipitate was formed. The precipitate was decanted, washed four times with water (5 mL each)and dried overnight at 50 °C. The Cu(II) complexes were characterized by ESI-MS, EPR spectroscopy and UV–vis spectrophotometry (Figure 3, Figures S6–S8).

All CW-EPR spectra were recorded with a BRUKER EleXsys E500 spectrometer (microwave frequency 9.5 GHz, microwave power 13 mW, modulation amplitude 5 G, modulation frequency 100 kHz). The powder of the Cu(II) complexes was dissolved in DMSO (the concentration was 3 mM) and measured in capillaries at room temperature. Frozen solution EPR spectra were measured for samples of 0.2 mL in quartz EPR tubes (0.05 mL MeOH was added to each sample to avoid line broadening upon freezing) and measured in a dewar containing liquid nitrogen (77 K). The frozen solution EPR spectra of CuCl_2_ in EMEM, RPMI 1640 cell culture media and in human blood serum were recorded at a concentration of 2 mM and in HEPES (0.6 mM, pH 7.4). The Cu(II)-estradiol-SC and Cu-(II)-Me_2_-estradiol-TSC complexes were dissolved in HEPES, EMEM, RPMI 1640 media and in serum in 1.7 mM concentration and measured in frozen solution (77 K).

EPR spectra were simulated by the EPR program [32]. Rhombic or axial *g*- and *A*-tensors (*I_Cu_* = 3/2) and one nitrogen *a_N_*-tensor (*I_N_* = 1) were taken into account. Nitrogen splitting was well resolved on most of the measured spectra. For the description of the linewidth, the orientation-dependent *α*, *β* and *γ* parameters were used to set up each component spectra, where α, β and γ defined the linewidths through the equation σMI = α + βMI + γMI^2^, where MI denotes the magnetic quantum number of the paramagnetic metal ions. Since natural CuCl_2_ was used for the measurements, both the isotropic and anisotropic spectra were calculated as the sum of the spectra of ^63^Cu and ^65^Cu weighted by their natural abundances. Further details of the simulation process were reported previously [32].

Complex of estradiol-SC as [CuLH_−1_]: yield: 62%; Anal. Calcd for C_20_H_25_CuN_3_O_3_ × 1.75H_2_O: C, 53.32; H, 6.38; N, 9.33. Found: C, 53.32; H, 6.06; N, 9.14. ESI-MS (methanol, positive): *m*/*z* 419.1261 [M + 1]^+^, 419.1270 calcd. for C_20_H_25_N_3_O_3_Cu (Figure S6); λ_max_ (nm) in methanol: 265, 303 and 383 nm.

Complex of estradiol-TSC as [CuLH_−1_]: yield: 68%; Anal. Calcd for C_20_H_25_CuN_3_O_2_S × 1.3H_2_O × 0.4CH_3_OH: C, 51.99; H, 6.25; N, 8.92; S, 6.8. Found: C, 52.24; H, 6.4; N, 8.45; S, 6.4. ESI-MS (methanol, positive): *m*/*z* 435.1038 [M *+* 1]^+^, 435.1042 calcd. for for C_20_H_26_CuN_3_O_2_S (Figure S6); λ_max_ (nm) in methanol: 318 and 390 nm.

Complex of Me-estradiol-TSC as [CuLH_−1_]: yield: 73%; Anal. Calcd for C_21_H_27_CuN_3_O_2_S × 1.3H_2_O: C, 53.38; H, 6.31; N, 8.89; S, 6.79. Found: C, 53.60; H, 6.3; N, 8.56; S, 6.51. ESI-MS (methanol, positive): *m/z* 449.1193 [M *+* 1]^+^, 449.1198 calcd. for C_21_H_28_CuN_3_O_2_S (Figure S6); λ_max_ (nm) in methanol: 322 and 400 nm.

Complex of Me_2_-estradiol-TSC as [CuLH_−1_]: yield: 85%; Anal. Calcd for C_22_H_29_CuN_3_O_2_S × 1.5 H_2_O: C, 53.91; H, 6.58; N, 8.57; S, 6.54. Found: C, 53.55; H, 6.44; N, 8.69; S, 6.81. ESI-MS (methanol, positive): *m*/*z* 463.1345 [M *+* 1]^+^, 463.1355 calcd. for C_22_H_30_CuN_3_O_2_S (Figure S6); λ_max_ (nm) in methanol: 320 and 401 nm.

### 3.4. X-ray Data Collection, Structure Solution and Refinement for the Estradiol-SC Complex

Needle-like brown crystal of [Cu(HL)Cl_2_] × H_2_O × 2CH_3_OH complex of estradiol-SC was mounted on a loop and measured by single crystal X-ray diffraction. Intensity data were collected on a Rigaku R-Axis Rapid diffractometer (graphite monochromator; Mo-Kα radiation, λ = 0.71073 Å) at 103(2) K. A numerical absorption correction was applied to the data using NUMABS [33] and CrystalClear [34] software. The structure was solved by direct methods using SIR [35] software and was refined using the SHELX [36] program package under WinGX [37] software. The structure was visualized using Mercury [38] software. Selected bond lengths and angles were calculated by PLATON8 software. The absolute structure parameter is 0.08(2) [39]. (Friedel coverage: 0.746, Friedel fraction max.: 0.996, Friedel fraction full: 0.996). The weighting scheme applied was w = 1/[σ^2^(F_o_^2^) + (0.02941.3299P)^2^ + 1.3299P], where P = (F_o_^2^ + 2F_c_^2^)/3. Hydrogen atomic positions were calculated from assumed geometries. Water hydrogens were restrained by DFIX to 0.85 Ångström O-H distance. Hydrogen atoms were included in structure factor calculations, but they were not refined. The isotropic displacement parameters of the hydrogen atoms were approximated from the U(eq) value of the atom they were bonded to. Crystal data and details of the structure determination and refinement are listed in Table S2. Bond length and angles, respectively, are listed in Tables S3 and S4. Hydrogen bond interactions are listed in Table S5. The crystallographic data files for the complexes have been deposited with the Cambridge Crystallographic Database as CCDC 2222678.

### 3.5. Solution Chemistry: UV–Visible Spectrophotometric Titrations, Kinetic Studies and Spectrofluorometric Measurements

An Orion 710A pH-meter equipped with a Metrohm combined electrode (type 6.0234.100) and a Metrohm 665 Dosimat burette were used for the UV–vis titrations at 25.0 ± 0.1 °C. Ionic strength of 0.10 M (KCl) was used to keep the activity coefficients constant. The titrations were performed with carbonate-free KOH solution of known concentration (0.10 M) with 30% (*v*/*v*) DMSO content. The electrode system was calibrated to the pH = −log[H^+^] scale by means of blank titrations (HCl vs. KOH) according to the method suggested by Irving et al. [40]. The average water ionization constant (p*K*_w_) is 14.54 ± 0.05 in the 30% (*v/v*) DMSO/H_2_O mixture. Argon was always passed over the solutions during the titrations. An Agilent Cary 8454 diode array spectrophotometer was used to record the UV–vis spectra at an interval of 200–800 nm. The path length was 2 cm. Spectrophotometric titrations were performed using a 0.1 M KOH titrant solution with 30% (*v/v*) DMSO on samples with 30% (*v/v*) DMSO containing HCl, KCl and the ligand at 20 μM concentration in the pH range 2.0–12.5 in the absence or in the presence of 1 or 0.5 equiv. Cu(II) ions. Proton dissociation constants (*K*_a_) of the TSC ligands, the overall stability constants (*β*) of the Cu(II) complexes and the UV–vis spectra of the individual species were calculated by the computer program PSEQUAD [41] as was done in our previous works [17,22].

The redox reaction of the Cu(II) complexes with GSH and ascorbic acid was studied at 25.0 ± 0.1 °C, at pH 7.40 (10 mM HEPES with 0.1 M KCl) on an Agilent Cary 8454 diode array spectrophotometer using a special, tightly closed tandem cuvette (Hellma Tandem Cell, 238-QS). The complex and the reducing agent were separated until the reaction was triggered. Both isolated pockets of the tandem cuvette were completely deoxygenated by bubbling argon for 10 min before mixing the reactants. Spectra were recorded before and then immediately after the mixing, and changes were followed till no further absorbance change was observed. The stock solutions of the reducing agents and the complexes were freshly prepared every day.

During the calculations, the absorbance (A)−time (t) curves were fitted and analyzed at the λ_max_ of the complex. (*A*_0_ − *A_final_*) × *e ^(−a×t)^* + *A_final_* equation was used where *A*_0_, *A_final_* and a parameters were refined and accepted at the minimal value of the weighted sum of squared residuals (difference between the measured and calculated absorbance values) at the given wavelength. Then observed rate constants (*k_obs_*) of the redox reaction were obtained from the data points of the simulated absorbance–time curves as the slope of the ln(*A/A*_0_) versus t plots.

The fluorescence three-dimensional spectra for the ligands were recorded on a Hitachi-4500 spectrofluorometer using a 1 cm quartz cell. Samples contained 10 µM TSC ligand in pure water at pH 7.40 (adjusted by PBS buffer) at 25.0 ± 0.1 °C.

### 3.6. Electrochemical and Spectroelectrochemical Studies

Cyclic voltammograms of TSCs derivatives and their Cu(II) complexes at 25.0 ± 0.1 °C were recorded. nBu_4_NPF_6_ was used as a supporting electrolyte, and measurements were performed at pH 7.4. The complexes were obtained by mixing the ligand dissolved in DMSO with aqueous solution of CuCl_2_ in 1:1 ratio. The final concentration of stock was 1 mM. Measurements were performed on a conventional three-electrode system under argon atmosphere using an Autolab PGSTAT 204 potentiostat/galvanostat monitored with Metrohm’s Nova software [42]. Samples were purged for 10 min with argon before recording the cyclic voltammograms. Platinum electrode was used as the working and auxiliary electrode and Ag/AgCl/3 M KCl as the reference electrode. The electrochemical system was calibrated with an aqueous solution of K_3_[Fe(CN)_6_] (*E*_1/2_ = +0.458 V vs. NHE) [43]. Redox potentials were obtained at 10 mV/s scan rate in the range of −1.3 to +0.8 V.

In situ UV–vis spectroelectrochemical measurements were performed on a spectrometer (Avantes, Model AvaLight-DHc light source equipped with an AvaSpec-UL2048XL-EVO in the spectroelectrochemical cell kit (AKSTCKIT3) with the Pt-microstructured honeycomb working electrode, purchased from Pine Research Instrumentation (Lyon, France). The cell was positioned in the CUV–UV cuvette holder connected to the diode-array UV–vis spectrometer by optical fibers. The spectra were processed using the AvaSoft 8.1.1 software package.

### 3.7. DPPH Free Radical Scavenging Assay

The DPPH (Sigma-Aldrich) free radical scavenging capacity was studied at 25.0 ± 0.1 °C on an Agilent Cary 3500 spectrophotometer with tightly closed tandem cuvette according to our approach with some modifications [17]. The stock solution of DPPH (100 µM) was prepared in ethanol, and 0.5 mL of this solution was added to one pocket of the cuvette and 0.5 mL of the tested compound in different concentrations in ethanol was added to another. The final concentration of the studied compounds was in the range 5–50 µM, where for DPPH concentration, it was always 50 µM. A standard compound Trolox (Sigma-Aldrich) was used as a positive control. The percentage of the antioxidant activity (AA), IC_50_ values (the concentration of compound at which it reduced 50% of DPPH) and Trolox equivalent antioxidant capacity (TEAC) were calculated [44].

### 3.8. In Vitro DNA Cleavage

DNA cleavage ability of the free ligands and the metal complexes was studied using a modified pUC19 plasmid DNA (pUC119ΔH+N-N6) in 30% (*v/v*) DMSO/H_2_O solvent mixture 10 mM HEPES at pH 6.0 or 7.4. Precipitation was not detected in the presence of HEPES, most probably due to its weak coordination [45]. The sequence modifications served other purposes and did not alter the performance of the plasmid DNA in the nonspecific DNA cleavage experiment. A detailed description of the treatment of the components of the reaction mixtures is provided in the supplementary material.

In a typical reaction mixture, 10 µL aliquots containing 100 ng plasmid DNA, 20 µM ligand alone or in the presence of 18 µM CuCl_2_ with or without 1 mM ascorbic acid or glutathione were incubated at 37 °C up to 24 h. Cu(II) was added in 0.9:1 ratio to avoid free-metal ion in the solution due to pipetting error. Cu(II) was first added to the ligand solutions at pH 6.5, then the pH was increased to 7.4 or decreased to 6.0 by the addition of 100 mM HEPES at various pH values. Since HEPES does not have significant buffering capacity at pH 6.0, the required amount of 100 mM HEPES at various pH values (pH 5.3, 6.5, 7.4) was calculated and then mixed together to obtain the final desired 6.0 pH in the presence of the otherwise acidic glutathione or ascorbic acid solutions. The samples, which were not examined immediately after incubation, were flash frozen in liquid N_2_ and stored at –80 °C until the gel electrophoresis experiment. The reaction mixtures were separated on 1% (*w*/*v*) agarose gels, containing 500 ng/mL ethidium bromide (EtBr) at 100 V for 45 min (Cleaver Scientific MultiSUB Wide Midi). GeneRuler™ 1 kb Plus DNA Ladder (Thermo Scientific) served as a reference. Images were recorded by a Uvitec BTS 20MS gel documentation system.

### 3.9. Bacterial Cell Culture and MIC Determination

*Klebsiella pneumoniae* (ATCC 49619), Escherichia coli (ATCC 25922), *Staphylococcus aureus* (ATCC 25928) and *Enterococcus faecalis* (ATCC 29212) strains were used as Gram-negative and Gram-positive bacterial cultures. The stock solutions of the tested compounds (dissolved in 90% (*v/v*) DMSO/H_2_O at 10 mM concentration) were diluted in 100 μL of Mueller Hinton Broth, and then two-fold serial dilutions were performed [46]. As a starting point, 100 μM concentration of the compounds was used. Then, 10^−4^ dilution of an overnight bacterial culture in 100 μL of medium was added to each well, with the exception of the medium control wells. Minimum inhibitory concentration (MIC) values of compounds were determined according to our previous work [17].

### 3.10. Cell Culture

To test the biological effects of the Cu(II) complexes and the ligands on human cancer cells, DU-145 prostate, A549 lung, and MCF-7 breast adenocarcinoma cells, as well as the multidrug-resistant MCF-7 KCR breast cancer cells were utilized. All the cell lines were maintained in RPMI-1640 media (Biosera, Nuaille, France) complemented with 10% fetal bovine serum (FBS), 2 mM L-glutamine, 0.01% streptomycin and 0.005% penicillin, (Biosera, Nuaille, France) and were cultured under standard conditions in a 37 °C incubator at 5% CO_2_ and 95% humidity. The cell lines were originally obtained from ATCC.

### 3.11. MTT Cytotoxicity Assay

The effect of the complexes and of their ligands alone on the viability (on the metabolic activity) of human cancer cells was assessed by MTT assay. The tested compounds were dissolved in 90% (*v/v*) DMSO/H_2_O at 10 mM concentration. Equimolar solutions of the ligand CuCl_2_ salt were used for the cytotoxicity assays. The metal salts without ligands were also tested. For this, 1 × 10^4^ cells/well were seeded into 96-well plates and left to grow overnight. On the following day, cells were exposed to increasing concentrations of each compound (0; 2.5; 5; 10; 15; 20 µM). After a 24 h incubation, cells were washed with PBS buffer and then incubated with 0.5 mg/mL MTT (Sigma-Aldrich, St Louis, Missouri, MO, USA) for 1 h at 37 °C. The formazan crystals were solubilized in DMSO (Molar Chemicals, Halásztelek, Hungary), and the absorbance of samples was determined at 570 nm using a Synergy HTX plate reader (BioTek, Winooski, Vermont, VT, USA). Absorption corresponding to the untreated control samples was considered as 100%. The viability measurements were repeated three times using 3 independent biological replicates.

### 3.12. Lactate Dehydrogenase (LDH) Assay

LDH assay was performed on DU-145, MCF-7, MCF-7 KCR and A549 cells to detect the cytotoxicity (reduction in plasma membrane integrity) induced by estrone-TSC, estradiol-TSC, complexes Cu(II)-estrone-TSC and Cu(II)-estradiol-TSC. For this, 1 × 10^4^ cells were seeded into 96-well plate and left to grow. After a day, the cells were treated with 20 µM of either estrone-TSC, Cu(II)-estrone-TSC, estradiol-TSC, Cu(II)-estradiol-TSC or equivalent amounts of DMSO or CuCl_2_ solution. After 24 h incubation, 10 µL of supernatant was replaced with a new plate and 100 µL of LDH working solution (PromoKine, PromoCell GmbH, Heidelberg, Germany) was added to the wells according to the manufacturer’s instructions. After incubation for 30 min, the absorbance of the samples was measured at 450 nm with a Synergy HTX plate reader (BioTek, Winooski, VT, USA). The measurements were repeated three times using 3 independent biological replicates.

### 3.13. Dichlorodihydrofluorescein Diacetate (DCFDA) Staining

DCFDA staining assay was performed to measure the total intracellular ROS level upon treatments. For this, 1 × 10^5^ cells/well were seeded onto coverslips (VWR International, Radnor, Pennsylvania, PA, USA) placed in 24-well plates and left to grow. After 6 h, the cells were treated with 20 µM of estrone-TSC, Cu(II)-estrone-TSC, estradiol-TSC, Cu(II)-estradiol-TSC or with equivalent amounts of DMSO or CuCl_2_ solution for 6 h. After treatments, cells were washed with PBS then incubated for 1 h with 10 μM DCFDA solution. The fluorescence intensity of the samples was assessed by an OLYMPUS BX51 microscope with Olympus DP70 camera and was quantified using ImageJ software.

### 3.14. γH2AX Immunostaining Assay

In order to determine the degree of DNA damage induced by the treatments, DNA double-strand breaks were visualized by *γ*H2AX immunostaining. For this, 1 × 10^5^ cells/well were seeded onto coverslips in 24-well plates. On the following day, the samples were treated for 3 h with 20 µM of estrone-TSC, estradiol-TSC, complexes Cu(II)-estrone-TSC and Cu(II)-estradiol-TSC or with equivalent amounts of DMSO or CuCl_2_ solution. Then, cells were fixed with 4% formaldehyde (Molar Chemicals, Halásztelek, Hungary), permeabilized with 0.3% Triton-X-100 (Calbiochem, Merck Millipore, Darmstadt, Germany) and were blocked using 5% bovine serum albumin (BSA) (Sigma-Aldrich, St. Louis, MO, USA). Then, the samples were incubated with anti-γH2AX antibody (Thermo Fisher Scientific, Waltham, MA, USA) in 1:300 dilution followed by Alexa 488 fluorophore-conjugated goat anti-mouse secondary antibody (Abcam, Cambridge, UK) in 1:600 dilution. The fluorescence intensity of the stained samples was detected by an Olympus FV10i confocal microscope. For the positive control samples, DNA double-strand breaks were induced by exposing cells to a 2 Gy dose of ionizing radiation for 1 min using an X-ray system (RS320, Xstrahl Limited, Xstrahl Surrey, UK) at the Extreme Light Infrastructure Attosecond Light Pulse Source (ELI-ALPS) Research Institute.

## 4. Conclusions

Estradiol-based (thio)semicarbazones were synthesized through a condensation reaction of 2-formyl-estradiol with suitable (thio)semicarbazides, and their Cu(II) complexes were also prepared. These compounds were obtained in order to investigate the effect of the exchange of the estrone moiety to estradiol on the proton dissociation processes of the ligands, the stability, redox properties and anticancer activity of the compounds prepared. Moreover, the effect of different substitutions, such as methyl and dimethyl groups at the *N*-terminal atom, was monitored as well.

The proton dissociation processes were characterized by UV–vis spectrophotometric titrations in 30% (*v*/*v*) DMSO/H_2_O. p*K*_a_ values of the phenol groups of the salicyaldehyde (thio)semicarbazones were slightly higher than those of the estrone-TSC analogs. Based on these p*K*_a_ values, all studied ligands are neutral at physiological pH. The overall stability constants of the Cu(II) complexes were also determined by spectrophotometric titrations. Only mono-ligand complexes [CuL]^+^ and [CuLH_−1_] were found exclusively even at ligand excess in the acidic and neutral pH range. The ligands coordinate via the (O^−^,N,S^−^) or (O^−^,N,O^−^) donor set in the case of TSCs and the SC, respectively, which was confirmed by EPR spectroscopy. The coordination of the neutral ligand in the complex [Cu(HL)Cl_2_] was found in solid phase by X-ray crystallography for the Cu(II)-estradiol-SC complex. The estradiol-based complexes showed comparable stability in solution as Cu(II) complexes with estrone moiety. Combination of spectroelectrochemical and cyclic voltammetric techniques showed similar behavior of all investigated Cu(II)-TSC complexes, namely quasi-reversible processes for Cu(II) → Cu(I), Cu(I) → Cu(II) one-electron transfer were observed. Additionally, a redox reaction of the Cu(II) complexes with physiological reductant agents GSH and AA was followed by the UV–vis method and showed that complexes could be reduced efficiently by GSH, but only Cu(II)-estradiol-SC by ascorbate. The antioxidant activity of the investigated compounds was determined, and Me_2_-estradiol-TSC revealed antioxidant efficacy comparable to the gold-standard Trolox. In the presence of Cu(II), the antioxidant activity of the compounds was decreased.

Anticancer activity of the ligand and their complexes was tested on MCF-7, MCF-7 KCR, DU-145, and A549 cells as a first screening prior to further detailed investigations. Estradiol-(T)SCs did not show cytotoxicity, or only a minor effect was observed on these cells. Nevertheless, their Cu(II) complexes revealed high cytotoxic activity against the tested cancer cells. The highest activity was shown on MCF-7 and MCF-7 KCR cell lines by the Cu(II)-estrone-TSC complex (4.28 µM and 12.75 µM) and somewhat lower activity by the Cu(II)-estradiol-TSC complex (8.40 µM and 12.95 µM). On the other hand, the Cu(II)-Me_2_-estradiol-TSC complex was the most cytotoxic on DU-145 and A549 cancer cells. Additionally, a DCFDA test was performed with selected compounds, namely estradiol-TSC and estrone-TSC and their Cu(II) complexes, where the complexes displayed larger ROS formation in comparison to their corresponding ligands. The ROS production did not induce DNA damage or cleavage according to the *γ*H2AX immunostaining, while in vitro opposite effect can be observed in the presence of GSH and ascorbic acid by plasmid DNA cell-free electrophoresis assays. Furthermore, antibacterial activity was investigated on the Gram-negative *E. coli* and *K. pneumoniae* strains. Moderate antibacterial activity was obtained for the estradiol-SC and estradiol-TSC on the Gram-positive *S. aureus* bacterial strain, while the interaction of the ligands with Cu(II) ions resulted in a significant increase in the antibacterial effect.

## Figures and Tables

**Table 1:** p*K*_a_ values of the studied compounds determined by UV–vis spectrophotometric titrations in 30% (*v/v*) DMSO/H_2_O (*T* = 25 °C, *I* = 0.10 M (KCl)).

	p*K*_a_
estradiol-SC	10.02 ± 0.03
estradiol-TSC	9.23 ± 0.03
Me-estradiol-TSC	9.28 ± 0.03
Me_2_-estradiol-TSC	9.20 ± 0.03
estrone-SC	9.50 ^1^
estrone-TSC	9.00 ^2^
Me-estrone-TSC	9.21 ^3^
Me_2_-estrone-TSC	9.02 ± 0.03 ^4^

^1^ Data are taken from Ref. [23]; ^2^ data are taken from Ref. [8]; ^3^ data are taken from Ref. [22]; ^4^ determined in this work.

**Table 2:** Overall stability constants (*β*), p*K*_a_ of the Cu(II) complexes estradiol-(T)SCs and estrone-(T)SC for comparison, determined by UV–vis titrations, and calculated pCu values at pH 5.0, 6.0, 7.4 using *c_Cu_*(II) = 20 µM, *c_ligand_* = 20 µM. Observed rate constants (*k_obs_*) obtained for the direct redox reaction of the Cu(II) complexes (25 µM) with GSH (1.25 mM) at pH 7.4 under anaerobic conditions. (30% (*v/v*) DMSO/H_2_O; *T* = 25.0 °C; *I* = 0.1 M (KCl)).

	log*β* [CuL]^+^	log*β* [CuLH_−1_]	p*K_a_* [CuL]^+^	pCu_5.0_	pCu_6.0_ ^5^	pCu_7.4_ ^6^	*k_obs_* (min^−1^)
estradiol-SC	11.63 ± 0.03	5.16 ± 0.03	6.48	5.7	6.3	7.8	fast reaction
estradiol-TSC	13.79 ± 0.03	9.84 ± 0.03	3.95	7.8	9.3	12.1	0.07 ± 0.03
Me-estradiol-TSC	13.68 ± 0.03	9.59 ± 0.03	4.08	7.6	9.1	11.8	0.22 ± 0.02
Me_2_-estradiol-TSC	13.96 ± 0.03	10.11 ± 0.03	3.85	8.0	9.6	12.4	0.31 ± 0.06
estrone-SC	11.26 ^1^	4.82 ^1^	6.44 ^1^	5.8	6.3	7.5 ^1^	fast reaction ^1^
estrone-TSC	13.18 ^2^	9.44 ^2^	3.74 ^2^	7.7	9.2	11.9 ^2^	0.13 ^3^
Me-estrone-TSC	13.64 ^3^	9.66 ^3^	3.98 ^3^	7.7	9.2	11.9 ^3^	0.05 ^4^
Me_2_-estrone-TSC	13.36 ± 0.08 ^4^	9.44 ± 0.07 ^4^	3.92 ^4^	8.0	9.7	12.5 ^4^	0.05 ^4^

^1^ Data are taken from Ref. [23]; ^2^ data are taken from Ref. [8]; ^3^ data are taken from Ref. [22]; ^4^ determined in this work; ^5^ estimated pCu values at 6.0; ^6^ Estimated pCu values at 7.4.

**Table 3:** Isotropic EPR parameters obtained by the simulation of room temperature EPR spectra recorded in DMSO of Cu(II) complexes formed with estradiol-(T)SCs. The unit of the coupling values is 10^−4^ cm^−1^. The experimental errors were ±0.001 for *g*_0_ and ±1 G for couplings and linewidth parameters.

Complexes	Components	*g*_0_	*A*_0_	*a*_0_^N^	*α* (G)	*β* (G)	*γ* (G)	Component Ratio
Cu(II)-estradiol-SC	[CuLH_−1_]	2.099	56.6	15.4	36.0	−20.0	3.7	50%
	[CuL]^+^	2.118	48.0	14.3	54.0	−25.0	1.8	50%
Cu(II)-estradiol-TSC	[CuLH_−1_]	2.090	70.2	17.5	35.7	−21.3	3.0	100%
Cu(II)-Me-estradiol-TSC	[CuLH_−1_]	2.088	68.7	18.0	36.9	−23.9	5.4	100%
Cu(II)-Me_2_-estradiol-TSC	[CuLH_−1_]	2.086	67.1	17.5	39.8	−22.3	3.2	82%
	[CuL]^+^	2.102	56.3	17.0	35.2	−21.5	3.5	18%

**Table 4:** Anisotropic EPR parameters obtained for the Cu(II) complexes of estradiol-(T)SC ligands by the simulation of frozen solution (77 K) EPR spectra recorded in 20% (*v/v*) MeOH/DMSO. The unit of the coupling values is 10^−4^ cm^−1^ unit. The experimental errors were ±0.002 for *g*_x_ and *g*_y_, ±0.001 for *g*_z_, ±2 G for *A*_x_ and *A*_y_, and ±1 G for *A*_z_ and nitrogen couplings. Isotropic values of the *g*-tensor are calculated via equation: *g*_0_ = (*g*_x_ + *g*_y_ + *g*_z_)/3.

	Estradiol-SC	Estradiol-TSC	Me-Estradiol-TSC	Me_2_-Estradiol-TSC	Oligomers ^1^
*g*_x_	2.054	2.030	2.028	2.030	2.088
*g*_y_	2.063	2.056	2.054	2.056	–
*g*_z_	2.299	2.209	2.206	2.222	–
*A*_x_	19.22	23.7	25.7	24.2	44
*A*_y_	4.8	17.8	17.9	15.7	–
*A*_z_	166.4	180.0	178.1	174.2	–
*a*_x_^N^	12.1	14.6	14.4	4.4	–
*a*_y_^N^	15.6	10.1	9.9	9.9	–
*a*_z_^N^	12.9	8.6	8.4	14.4	–
*g*_0,calc_	2.139	2.098	2.096	2.103	–

^1^ Broad singlet spectra were treated with isotropic parameters.

**Table 5:** DPPH free radical scavenging activity of estradiol-(T)SC, estrone-TSC, STSC, estradiol and Trolox expressed as IC_50_ and Trolox equivalent antioxidant capacity (TEAC) values, and % scavenging effect at 1:1 DPPH-to-compound ratio after 30 min. (*T* = 25 °C; in ethanol, *c_DPPH_* = 50 µM; *c_compound_* = 5−50 µM).

	IC_50_ (µM)	% Scavenging Effect ^1^	TEAC
estradiol-SC	>50 ^2^	6.5	-
estradiol-TSC	14.6	72.0	0.8
Me-estradiol-TSC	15.4	31.0	0.8
Me_2_-estradiol-TSC	13.6	100	0.9
estrone-TSC	17.8	49.8	0.7
STSC	19.0	23.4	0.6
estradiol	>50 ^2^	0.5	-
estradiol-SC+1 equiv. Cu(II)	>50 ^2^	8.5	-
estradiol-TSC+1 equiv. Cu(II)	15.7	90.8	0.8
Me-estradiol-TSC+1 equiv. Cu(II)	20.3	33.6	0.6
Me_2_-estradiol-TSC+1 equiv. Cu(II)	16.4	40.4	0.7
estrone-TSC+1 equiv. Cu(II)	34.8	11.1	0.3
STSC+1 equiv. Cu(II)	>50 ^2^	29.4	-
Trolox	12	100	1

^1^ At DPPH:compound = 1:1 ratio after 30 min. ^2^ Inhibition of 50% was not reached under the conditions used.

**Table 6:** MIC values determined for the ligands in the absence and presence of 1 equiv. Cu(II) on Gram-positive bacteria strains.

	MIC (µM)	
	*E. faecalis* ATCC 29212	*S. aureus* ATCC 25928
estradiol-SC	>100	50
estradiol-TSC	>100	25
Me-estradiol-TSC	>100	>100
Me_2_-estradiol-TSC	>100	>100
estrone-TSC	>100	>100
estradiol-SC+1 equiv. Cu(II)	>100	50
estradiol-TSC+1 equiv. Cu(II)	12.5	3.125
Me-estradiol-TSC+1 equiv. Cu(II)	50	6.25
Me_2_-estradiol-TSC+1 equiv. Cu(II)	12.5	0.78
estrone-TSC+1 equiv. Cu(II)	12.5	6.25
CuCl_2_	>100	>100

**Table 7:** IC_50_ values (µM) of the complexes and the ligands determined on various cancer cell lines. (24 h incubation time).

	IC_50_ Values (µM)			
	MCF-7	MCF-7 KCR	DU-145	A549
DMSO	>20	>20	>20	>20
CuCl_2_	>20	>20	>20	>20
estrone-TSC	>20	>20	>20	16.71 ± 1.03
Cu(II)-estrone-TSC	4.28 ± 1.06	12.75 ± 1.10	11.37 ± 1.05	4.03 ± 1.01
estradiol-SC	>20	>20	>20	>20
Cu(II)-estradiol-SC	>20	>20	>20	>20
estradiol-TSC	>20	>20	>20	20.54 ± 1.07
Cu(II)-estradiol-TSC	8.40 ± 1.11	12.95 ± 1.09	>20	10.03 ± 1.04
Me-estradiol-TSC	>20	>20	>20	15.79 ± 1.03
Cu(II)-Me-estradiol-TSC	16.74 ± 1.66	>20	15.84 ± 1.08	3.71 ± 1.02
Me_2_-estradiol-TSC	>20	>20	>20	>20
Cu(II)-Me_2_-estradiol-TSC	10.05 ± 1.36	>20	7.85 ± 1.08	3.44 ± 1.02

## Data Availability

Not applicable.

## Supplementary Materials

The following supporting information can be downloaded at: https://www.mdpi.com/article/10.3390/molecules28010054/s1, Figure S1:UV-vis absorption spectra of estradiol-SC; Figure S2: Individual UV-vis molar absorption spectra of the different ligand species calculated for the estradiol-SC and estradiol-TSC system; Figure S3: Three-dimensional fluorescence spectra of TSCs; Figure S4: UV-vis absorption spectra of the Cu(II)–estradiol-SC; Figure S5: Individual UV-vis molar absorption spectra of the different complex species calculated for the Cu(II)−estradiol-SC and Cu(II)−estradiol-TSC system; Figure S6: ESI-MS spectra of the Cu(II) complexes; Figure S7: UV-vis absorption spectra of ligands and Cu(II) complexes; Figure S8: Calculated frozen solution EPR spectra of Cu(II) complexes; Figure S9: Frozen solution EPR spectra of the Cu(II)-Me_2_-estradiol-TSC complex dissolved in different biological media; Figure S10: Frozen solution EPR spectra of the complex Cu(II)-estradiol-SC dissolved in different biological medium; Figure S11: Frozen solution EPR spectra of the complex CuCl_2_ dissolved in different biological medium; Table S1: Anisotropic EPR parameters of components; Table S2: Crystal data and details of structure refinement; Table S3: Bond lengths; Table S4: Bond angles; Table S5: Analysis of potential hydrogen bonds and; Figure S12: Time dependence of effect of bubbling O_2_ through the sample following the reaction with 50 equiv. GSH and Cu(II)-Me-estradiol-TSC complex; Figure S13: Time-dependent changes of the UV–vis spectra recorded for the Cu. The following supporting information can be downloaded at: https://www.mdpi.com/article/10.3390/molecules28010054/s1, Figure S1:UV-vis absorption spectra of estradiol-SC; Figure S2: Individual UV-vis molar absorption spectra of the different ligand species calculated for the estradiol-SC and estradiol-TSC system; Figure S3: Three-dimensional fluorescence spectra of TSCs; Figure S4: UV-vis absorption spectra of the Cu(II)–estradiol-SC; Figure S5: Individual UV-vis molar absorption spectra of the different complex species calculated for the Cu(II)−estradiol-SC and Cu(II)−estradiol-TSC system; Figure S6: ESI-MS spectra of the Cu(II) complexes; Figure S7: UV-vis absorption spectra of ligands and Cu(II) complexes; Figure S8: Calculated frozen solution EPR spectra of Cu(II) complexes; Figure S9: Frozen solution EPR spectra of the Cu(II)-Me_2_-estradiol-TSC complex dissolved in different biological media; Figure S10: Frozen solution EPR spectra of the complex Cu(II)-estradiol-SC dissolved in different biological medium; Figure S11: Frozen solution EPR spectra of the complex CuCl_2_ dissolved in different biological medium; Table S1: Anisotropic EPR parameters of components; Table S2: Crystal data and details of structure refinement; Table S3: Bond lengths; Table S4: Bond angles; Table S5: Analysis of potential hydrogen bonds and; Figure S12: Time dependence of effect of bubbling O_2_ through the sample following the reaction with 50 equiv. GSH and Cu(II)-Me-estradiol-TSC complex; Figure S13: Time-dependent changes of the UV–vis spectra recorded for the Cu–estradiol-SC complex in the presence of 50 equiv. ascorbic acid; Figure S14: Nuclease activity of TSC derivatives and their Cu(II)-complexes; Figure S15: Nuclease activity of TSC derivatives and their Cu(II)-complexes; Figure S16: Nuclease activity of 1 mM ascorbic acid with increasing amounts of Cu(II); Figure S17: Nuclease activity of TSC derivatives and their Cu(II)-complexes; Figure S18: Viability measured on MCF-7 cells upon the treatment with estradiol-SC, estradiol-TSC, Me-estradiol-TSC, Me_2_-estradiol-TSC, estrone-TSC and their Cu(II) complexes at various concentrations; Figure S19: Viability measured on A549 cells upon the treatment with estradiol-SC, estradiol-TSC, Me-estradiol-TSC, Me_2_-estradiol-TSC, estrone-TSC and their Cu(II) complexes at various concentrations; Figure S20: Viability measured on DU-145 cells upon the treatment with estradiol-SC, estradiol-TSC, Me-estradiol-TSC, Me_2_-estradiol-TSC, estrone-TSC and their Cu(II) complexes at various concentrations; Figure S21: Viability measured on MCF-7-KCR cells upon the treatment with estradiol-SC, estradiol-TSC, Me-estradiol-TSC, Me_2_-estradiol-TSC, estrone-TSC and their Cu(II) complexes at various concentrations; Figure S22: LDH assay to measure the reduction in plasma membrane integrity; Figure S23: Fluorescence microscopic images of DCFDA staining assays; Figure S24: Level of ROS generation by DCFDA staining; Figure S25: Fluorescence microscopic images of the γH2AX immunostaining; Figure S26: ^1^H NMR spectra of the TSC; Figure S27: ^13^C NMR spectra of TSC; Figure S28: ESI-MS spectra of the TSCs.

## Sample Availability

Samples of the compounds are available from the authors.
