# Supplementary material for: Estradiol-Based Salicylaldehyde (Thio)Semicarbazones and Their Copper Complexes with Anticancer, Antibacterial and Antioxidant Activities
Source: Molecules. 2022 Dec 21;28(1):54. doi: 10.3390/molecules28010054 (PMC9822434; doi:10.3390/molecules28010054)
Supplement: Supplementary file 1 [file molecules-28-00054-s001.zip › molecules-2098355-supplementary.pdf]

Supporting Information for

Estradiol-based salicylaldehyde (thio)semicarbazones and their copper complexes with anticancer, antibacterial and antioxidant activities

Tatsiana V. Petrasheuskaya, Ferenc Kovács, Nóra Igaz, Andrea Rónavári, Bálint Hajdu, Laura Bereczki, Nóra V. May, Gabriella Spengler, Béla Gyurcsik, Mónika Kiricsi, Éva Frank, Éva A. Enyedy*

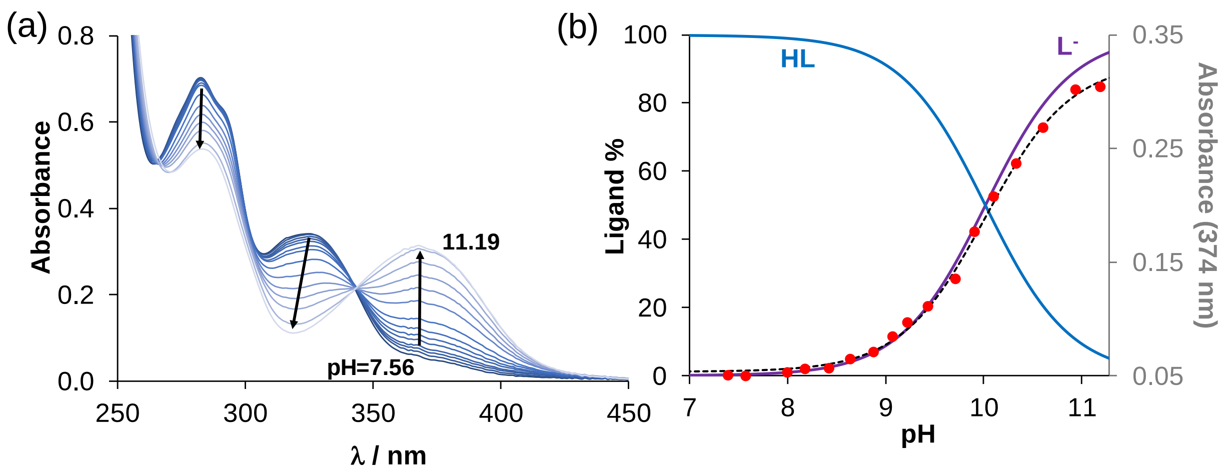

Figure S1. (**a**) UV-vis absorption spectra of estradiol-SC recorded at various pH values in 30% (*v*/*v*) DMSO/H_2O solvent mixture. (c_{ligand} = 20 μM; *T* = 25.0 °C; *I* = 0.1 M (KCl); ℓ = 2 cm) (**b**) Concentration distribution curves for this ligand with absorbance values measured at 374 nm (●) with the fitted curve (dashed line).

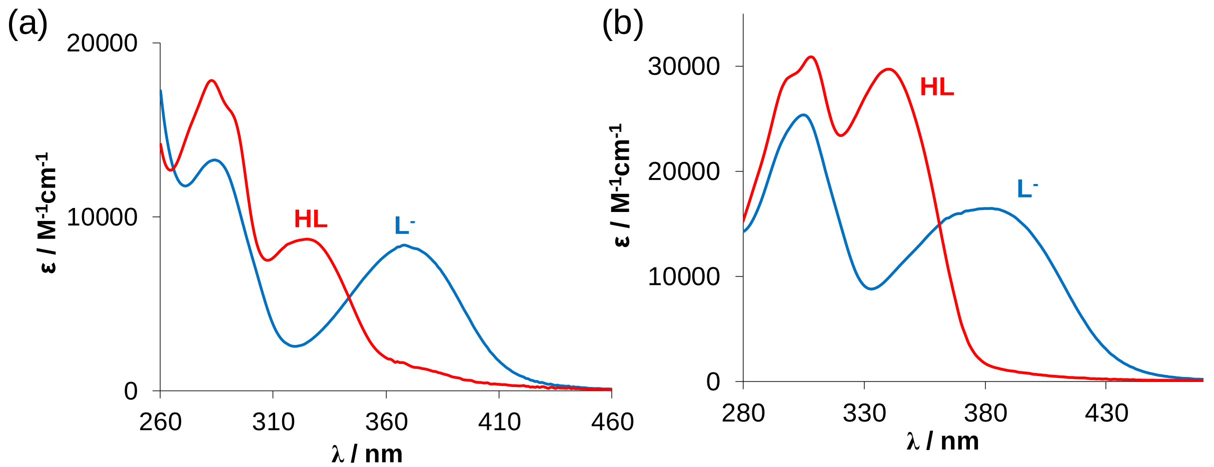

Figure S2. Individual UV-vis molar absorption spectra of the different ligand species calculated for (**a**) estradiol-SC and (**b**) estradiol-TSC in 30% (v/v) DMSO/H_2O solvent mixture. (c_{ligand} = 20 μM; *T* = 25.0 °C; *I* = 0.1 M (KCl))

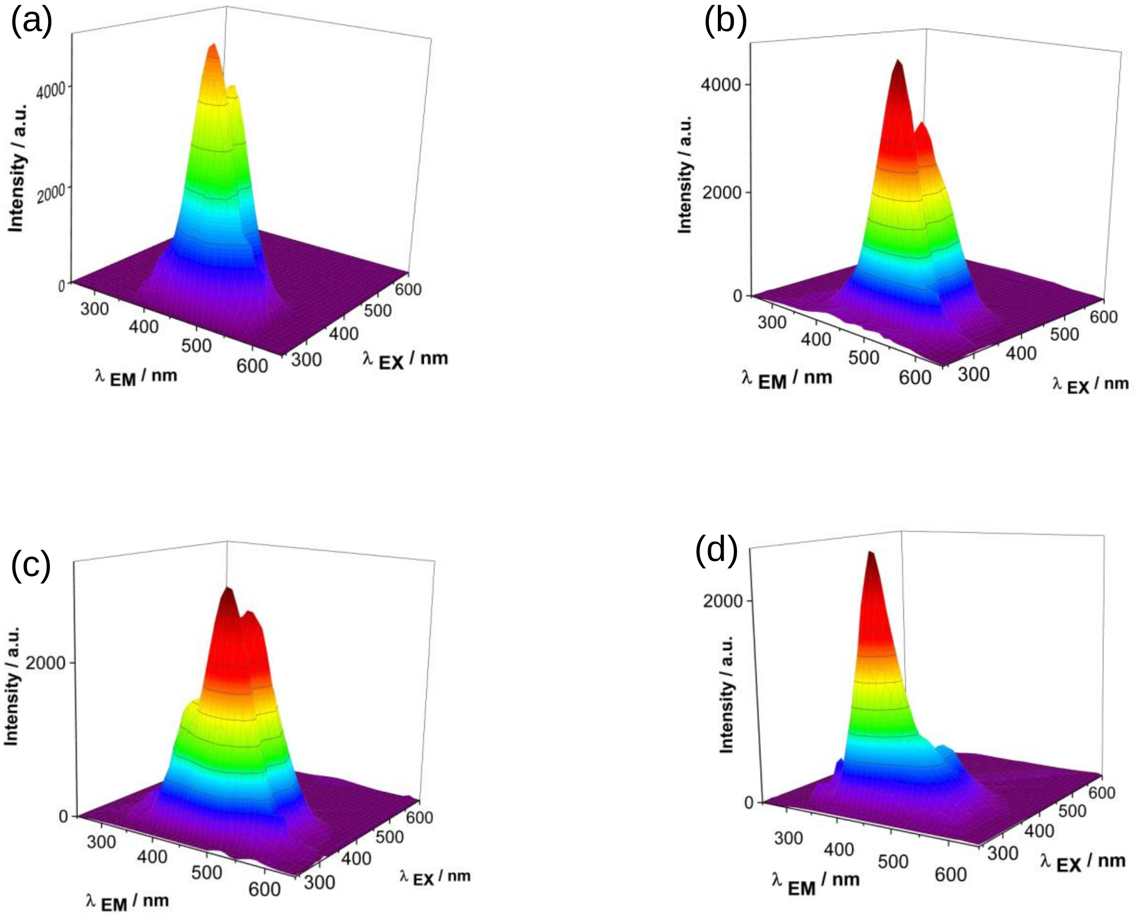

Figure S3. Three-dimensional fluorescence spectra of (**a**) estradiol-SC, (**b**) estradiol-TSC, (**c**) Me-estradiol-TSC and (**d**) Me_2-estradiol-TSC in H_2O at pH 7.4. ($c_{compound}$ = 10 μM; I = 0.1 M (KCl); T = 25.0 °C).

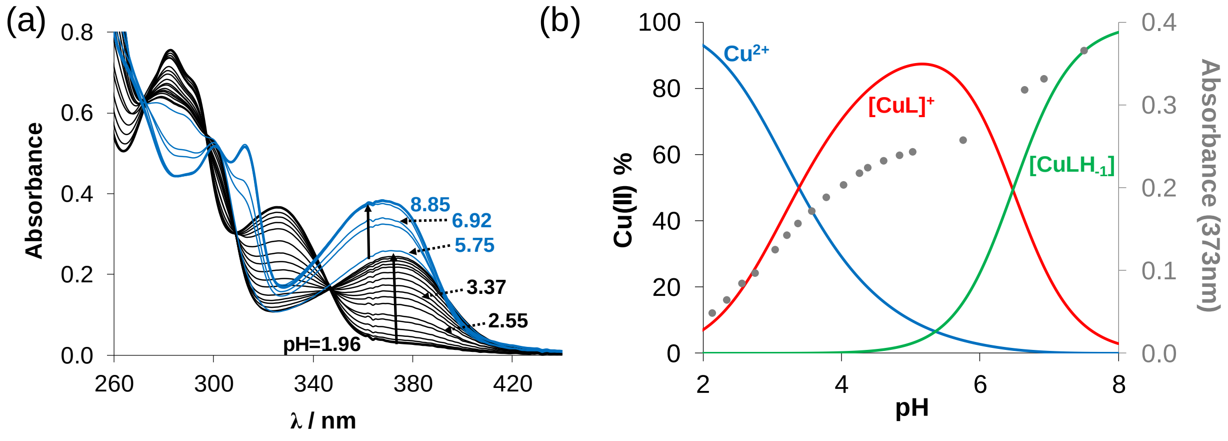

Figure S4. (**a**) UV-vis absorption spectra of the Cu(II) – estradiol-SC (1:1) system in the pH range 1.96–8.85 in 30% (v/v) DMSO/H_2O solvent mixture. (**b**) Concentration distribution curves for the same system plotted together with the absorbance changes at 373 nm (●). (c_{ligand} = 20 μM; $c_{Cu(II)}$ = 20 μM; T = 25.0 °C; I = 0.1 M (KCl); ℓ = 2 cm).

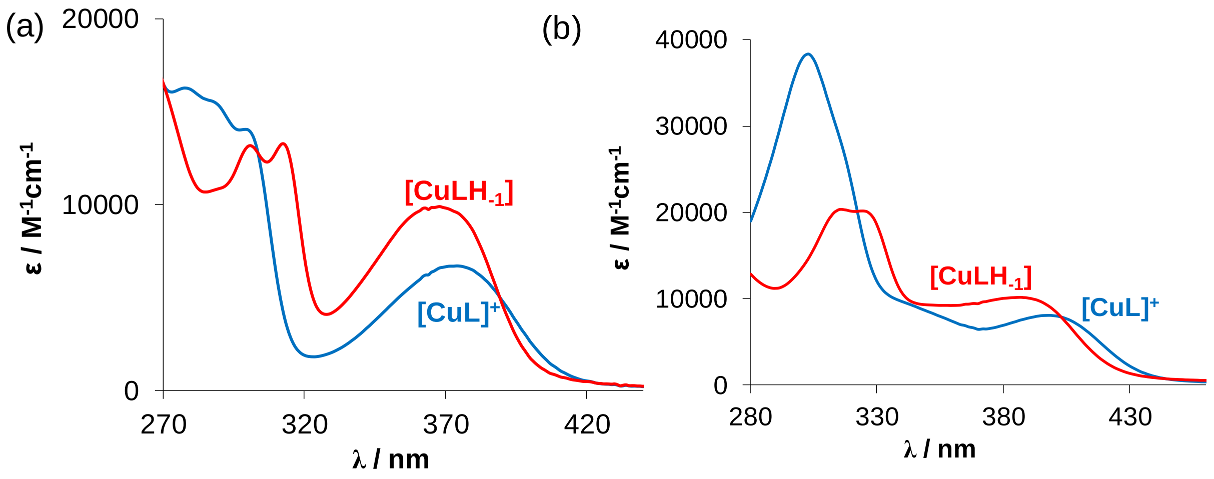

Figure S5. Individual UV-vis molar absorption spectra of the different complex species calculated for the (**a**) Cu(II) – estradiol-SC and (**b**) Cu(II) – estradiol-TSC system in 30% (*v*/*v*) DMSO/H_2O solvent mixture. (c_{ligand} = 20 μM; $c_{Cu(II)}$ = 20 μM; T = 25.0 °C; I = 0.1 M (KCl)).

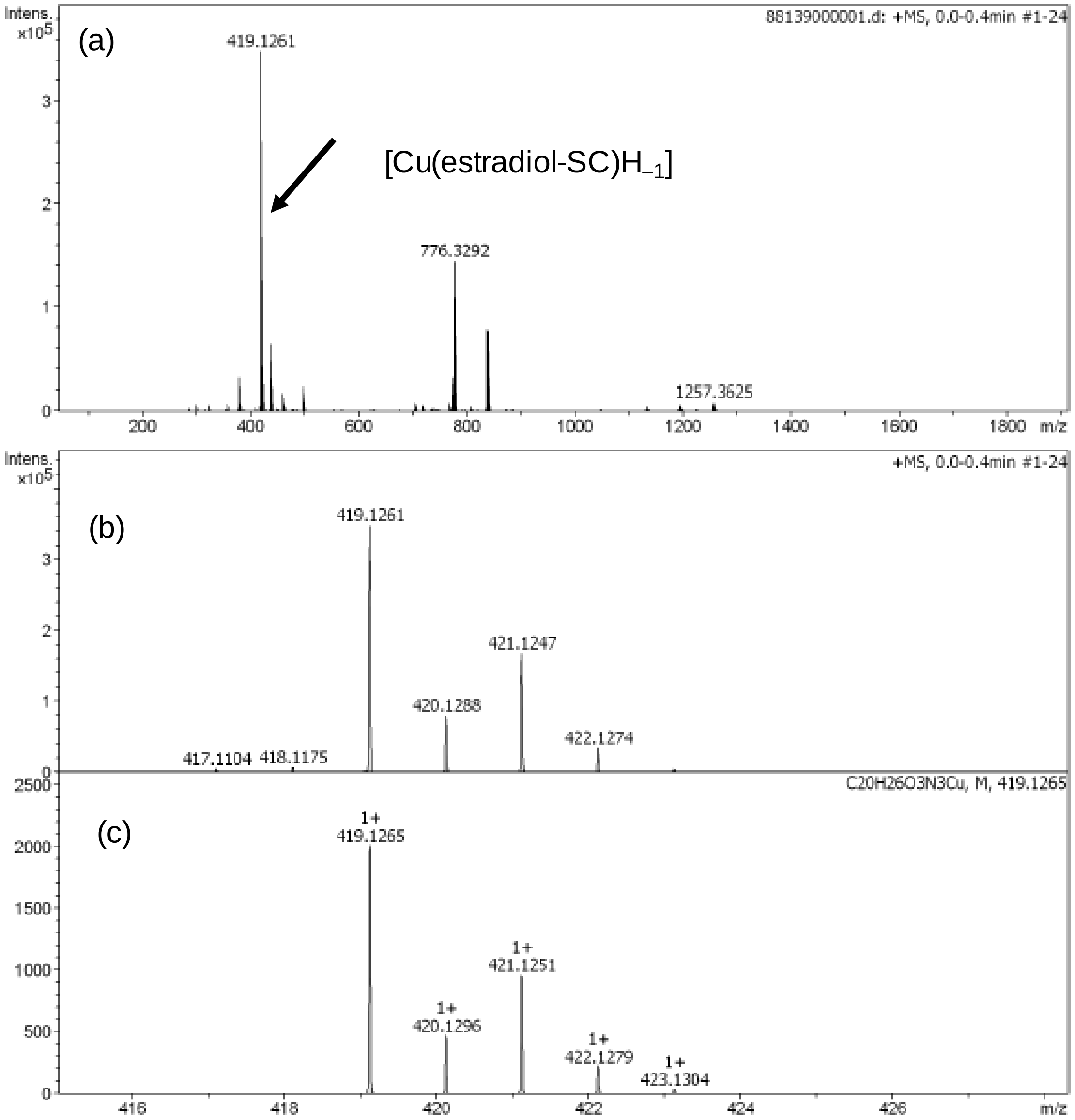

(a)
419.1261
[Cu(estradiol-SC)H₋₁]
776.3292
1257.3625
88139000001.d: +MS, 0.0-0.4min #1-24
Intens. x10^5
m/z
(b)
419.1261
420.1288
421.1247
422.1274
417.1104
418.1175
+MS, 0.0-0.4min #1-24
(c)
1+ 419.1265
1+ 420.1296
1+ 421.1251
1+ 422.1279
1+ 423.1304
C20H26O3N3Cu, M, 419.1265

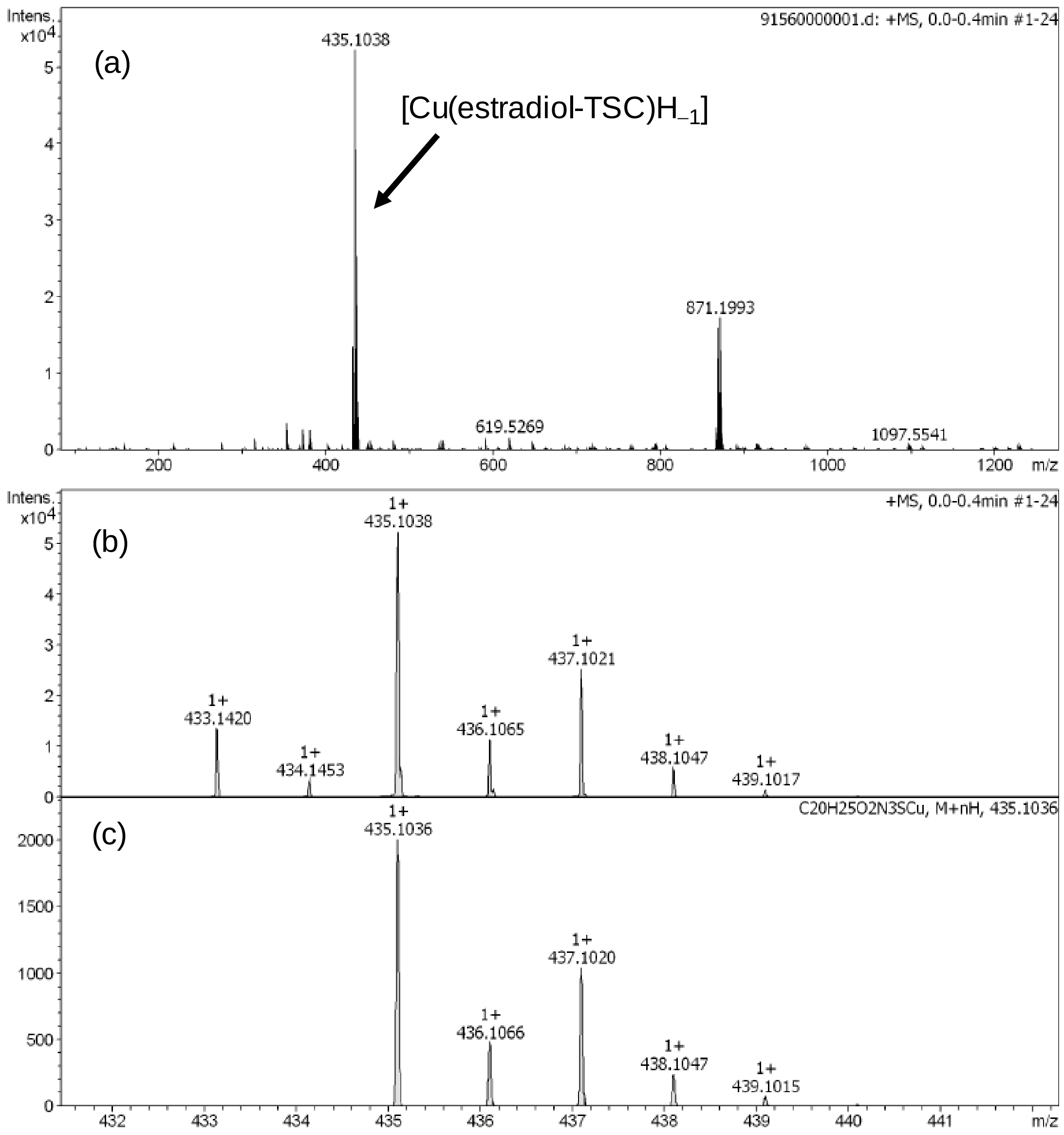

(a)
[Cu(estradiol-TSC)H–1]
435.1038
871.1993
619.5269
1097.5541
91560000001.d: +MS, 0.0-0.4min #1-24
(b)
+MS, 0.0-0.4min #1-24
1+ 433.1420
1+ 434.1453
1+ 435.1038
1+ 436.1065
1+ 437.1021
1+ 438.1047
1+ 439.1017
(c)
C20H25O2N3SCu, M+nH, 435.1036
1+ 435.1036
1+ 436.1066
1+ 437.1020
1+ 438.1047
1+ 439.1015
Intens. x10^4
m/z

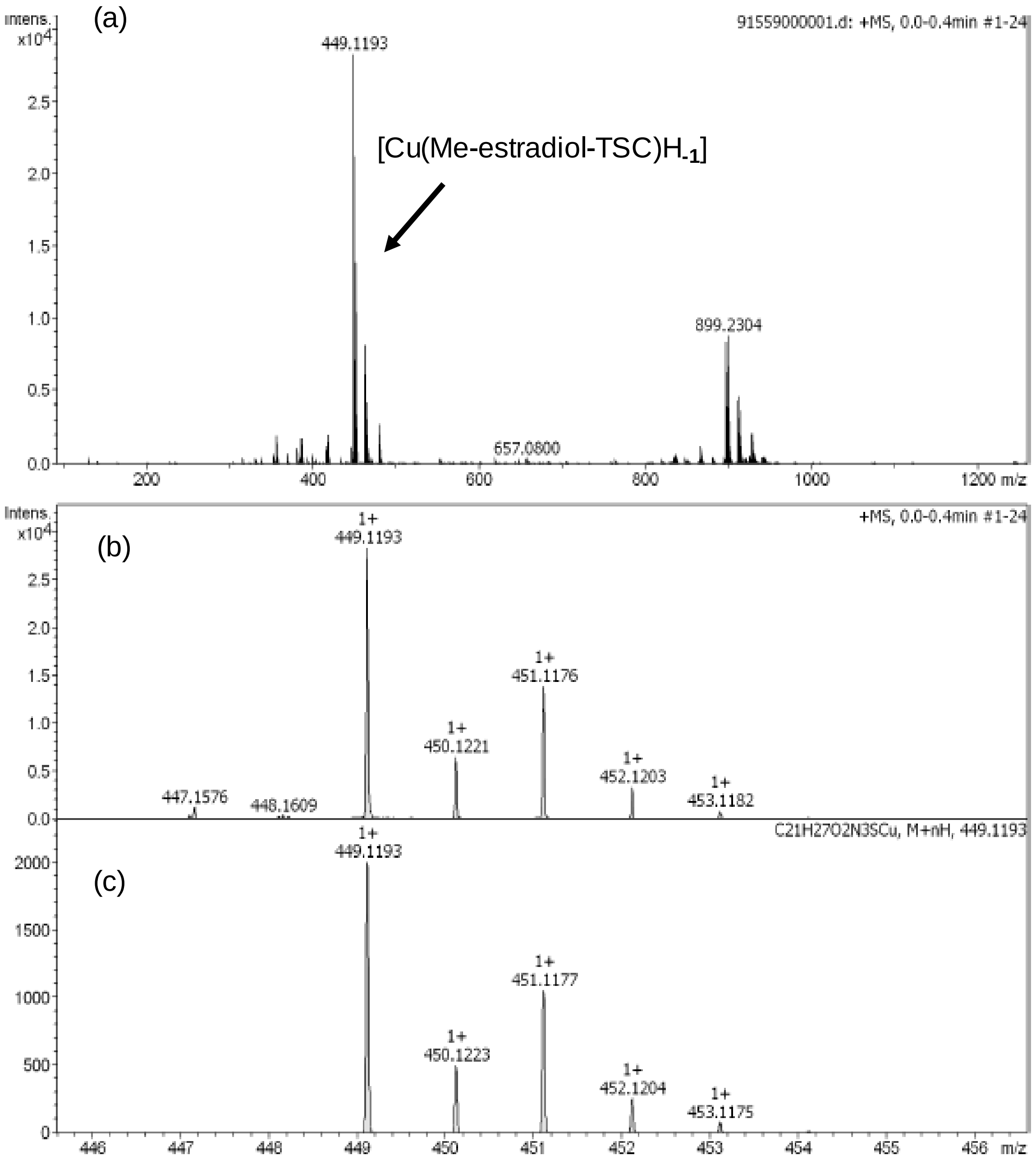
(a)
Intens.
x10^4
91559000001.d: +MS, 0.0-0.4min #1-24
449.1193
[Cu(Me-estradiol-TSC)H-1]
899.2304
657.0800
m/z
(b)
+MS, 0.0-0.4min #1-24
1+
449.1193
1+
451.1176
1+
450.1221
1+
452.1203
1+
453.1182
447.1576
448.1609
(c)
C21H27O2N3SCu, M+nH, 449.1193
1+
449.1193
1+
451.1177
1+
450.1223
1+
452.1204
1+
453.1175
m/z

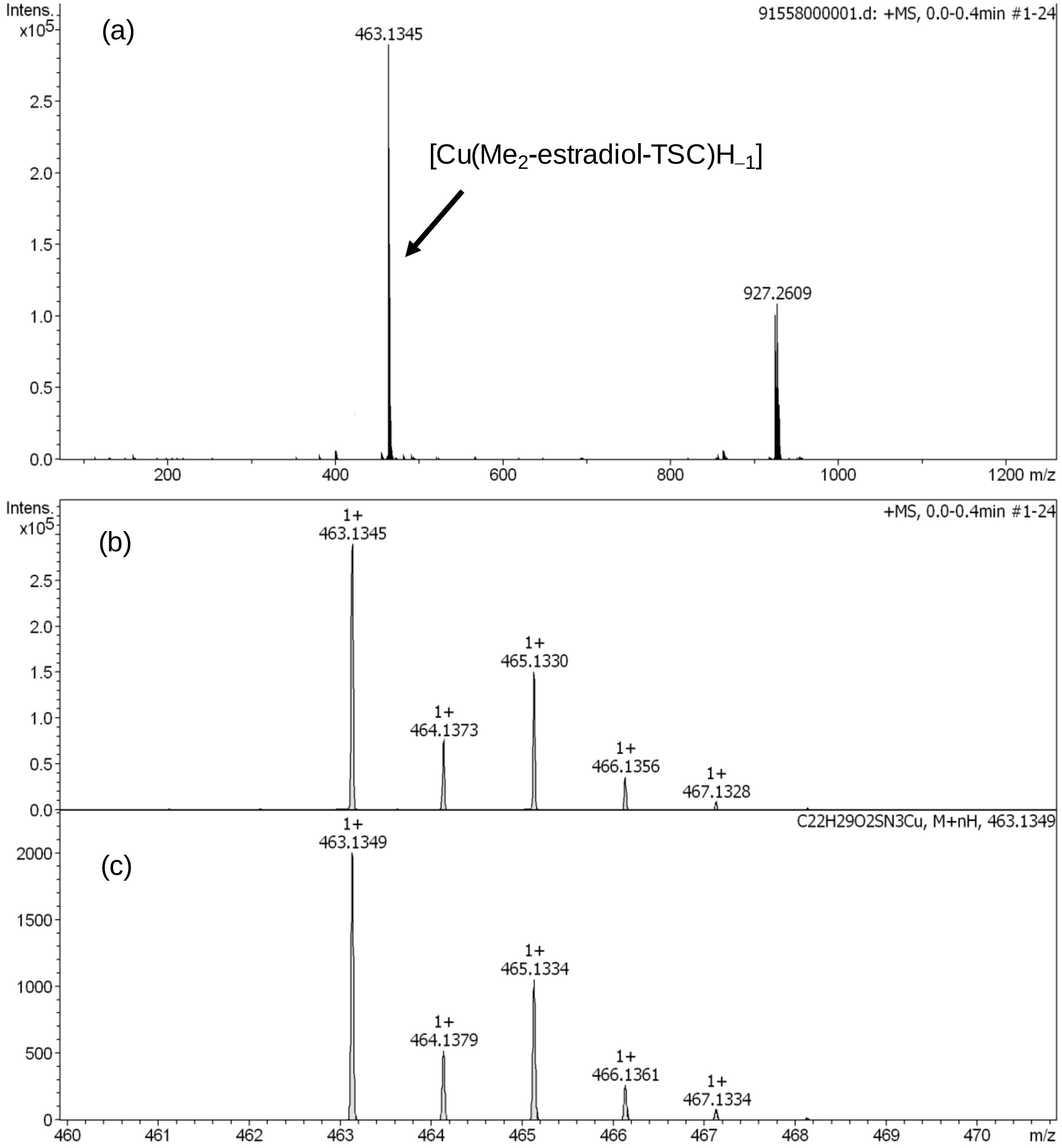

Figure S6. ESI-MS spectra of the indicated Cu(II) complexes of the title thiosemicarbazones. (**a**) Measured, (**b**) zoomed range, (**c**) simulated MS spectra. Samples were prepared in methanol.

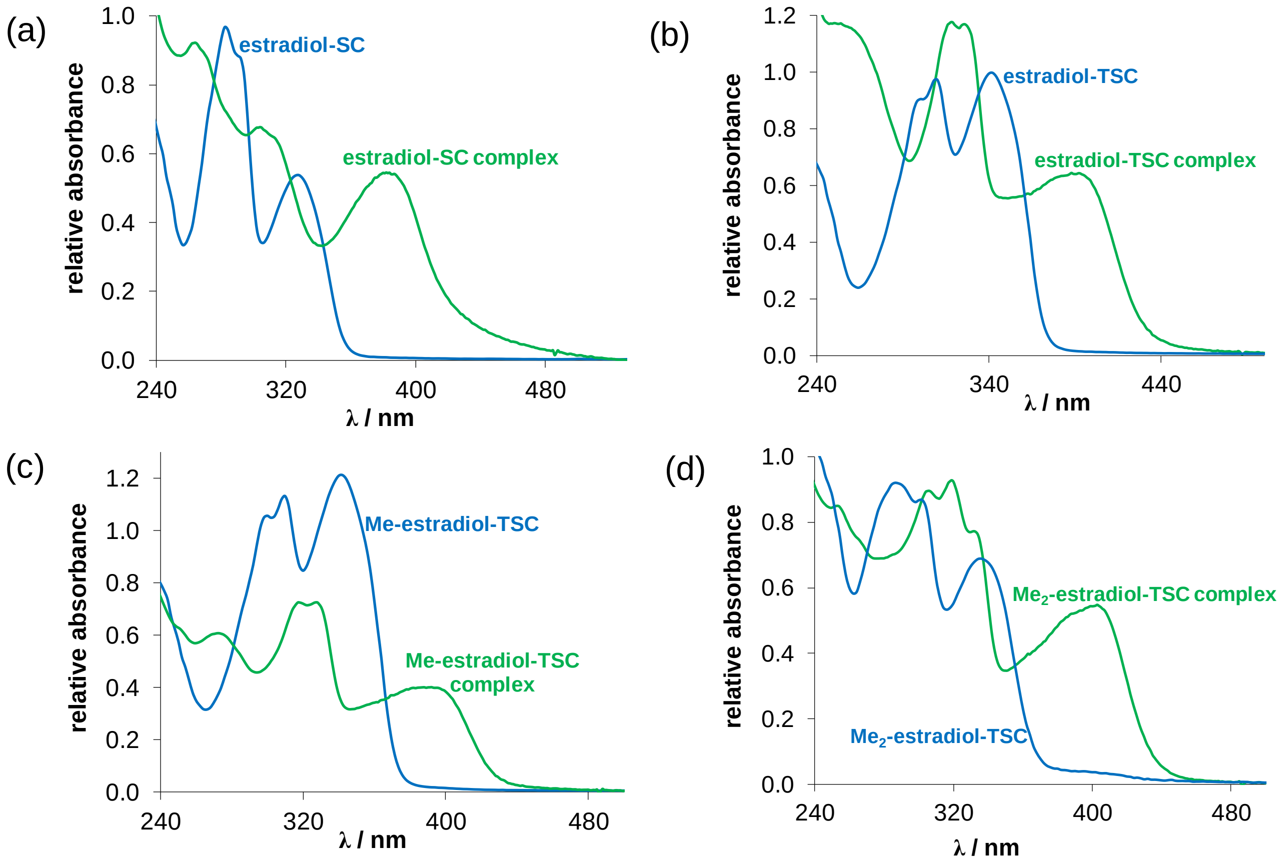

Figure S7. UV-vis absorption spectra recorded in methanol for the isolated ligands and Cu(II) complexes: (**a**) estradiol-SC and Cu(II)-estradiol-SC, (**b**) estradiol-TSC and Cu(II)-estradiol-TSC, (**c**) Me-estradiol-TSC and Cu(II)-Me-estradiol-TSC, (**d**) Me_2-estradiol-TSC and Cu(II)-Me_2-estradiol-TSC.

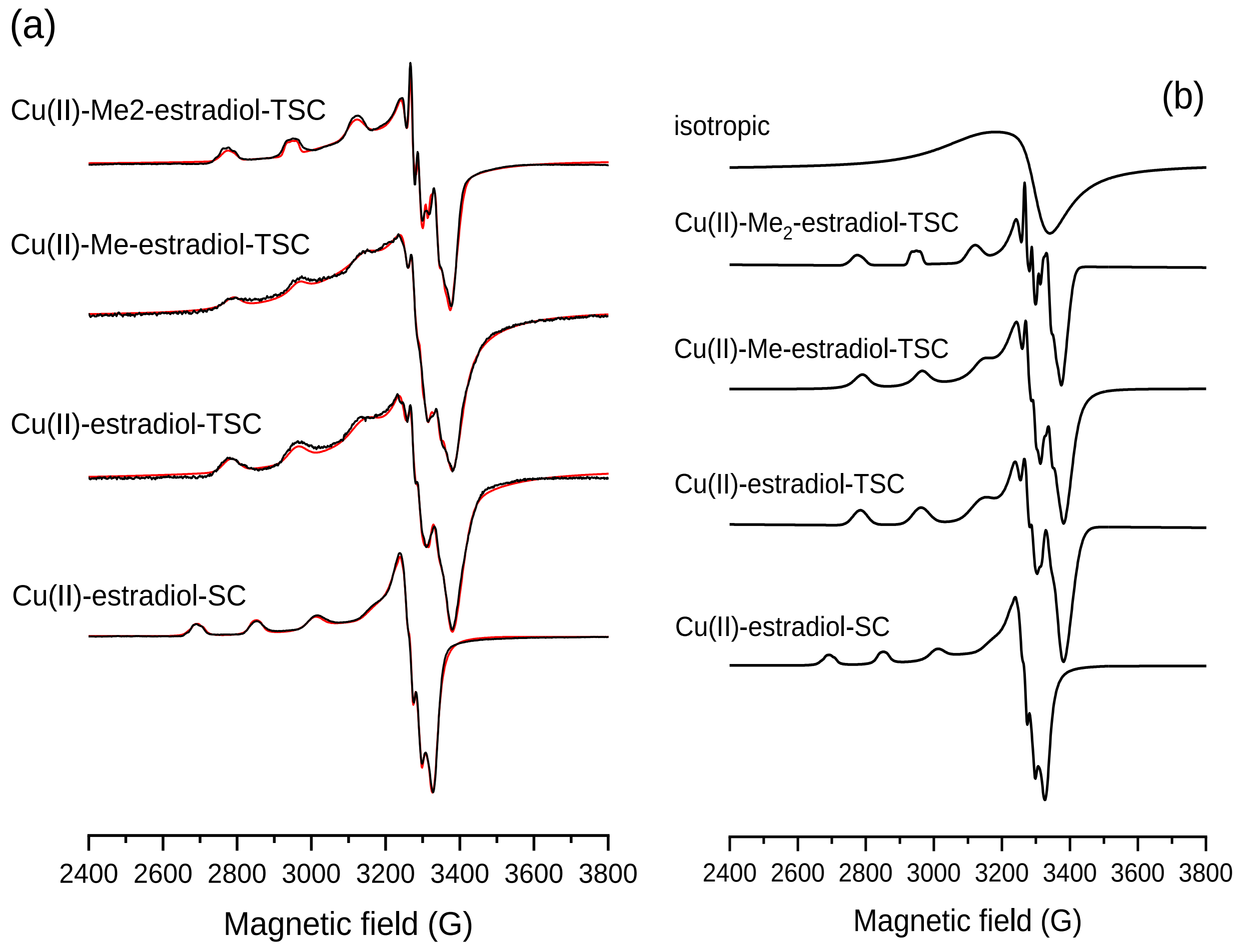

Figure S8. Frozen solution EPR spectra of Cu(II) complexes of estradiol-SC, estradiol-TSC, Me-estradiol-TSC and Me_2-estradiol-TSC dissolved in 20% (*v*/*v*) MeOH/DMSO. (**a**) Measured spectra in black and the simulation curves in red. (**b**) Calculated component spectra. The isotropic component originated from oligomerisation process.

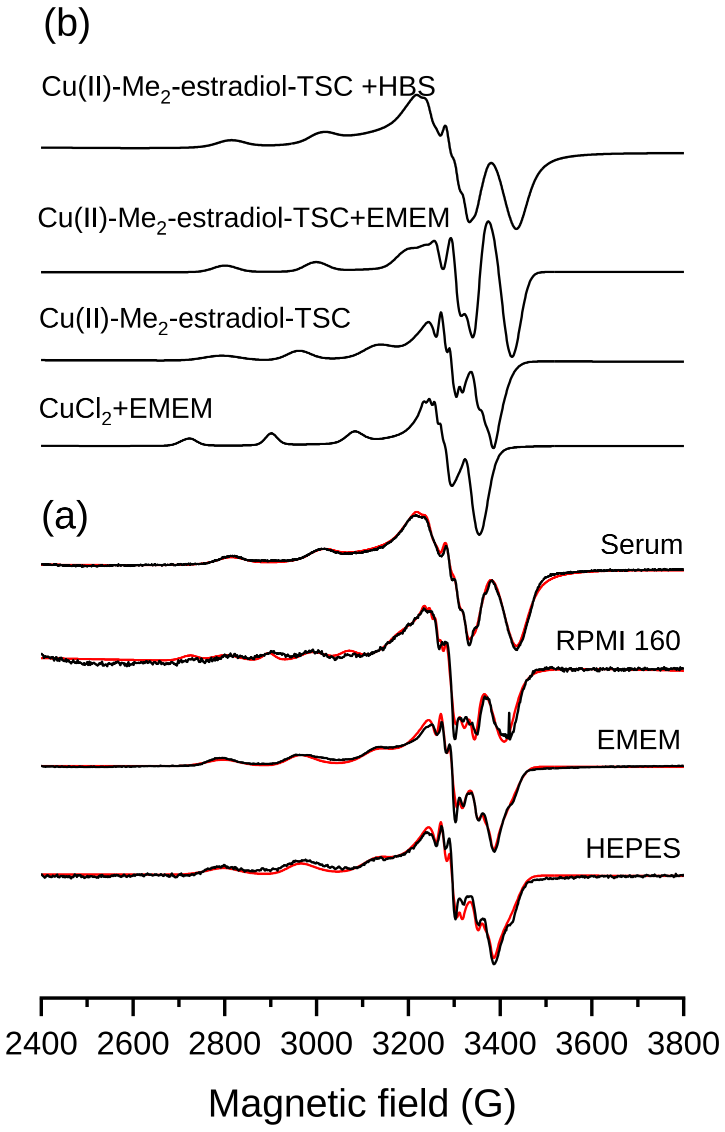

Figure S9. Frozen solution EPR spectra of the complex Cu(II)-Me_2-estradiol-TSC dissolved in different biological medium HEPES, EMEM, RPMI 1640 and HBS. (**a**) Measured spectra in black and the simulation curves in red. (**b**) Calculated component spectra. The measured spectra were simulated with 82% Cu(II)-Me_2-estradiol-TSC + 18% Cu(II)-Me_2-estradiol-TSC + EMEM in HEPES, 85% Cu(II)-Me_2-estradiol-TSC + 15% Cu(II)-Me_2-estradiol-TSC + EMEM in EMEM, 47% $CuCl_2$ + EMEM+ 53% Cu(II)-Me_2-estradiol-TSC + EMEM in RPMI 1640 and 8% Cu(II)-Me_2-estradiol-TSC + 92% Cu(II)-Me_2-estradiol-TSC + HBS in human blood serum.

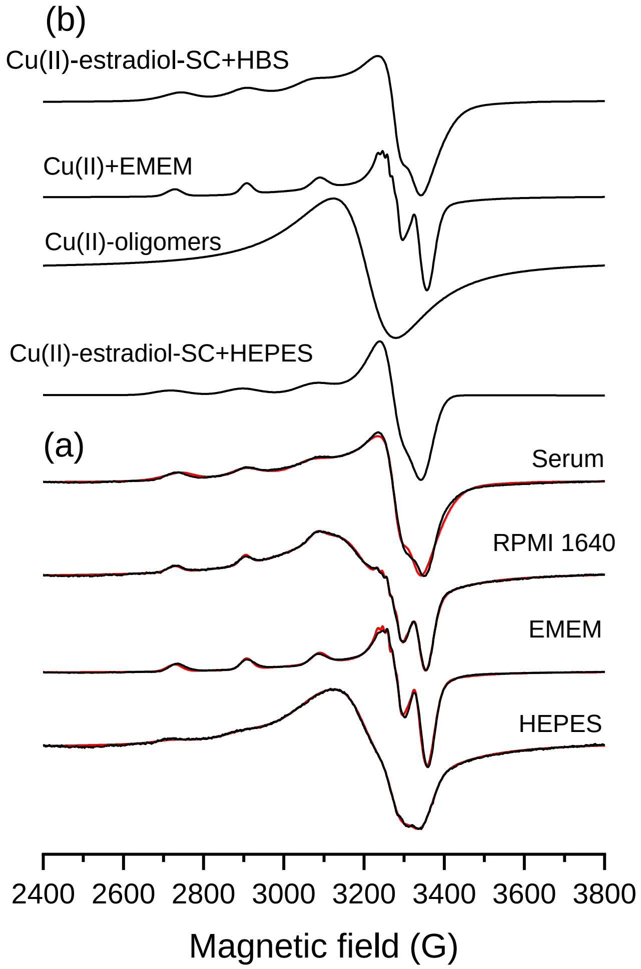

Figure S10. Frozen solution EPR spectra of the Cu(II)-estradiol-SC complex dissolved in different biological media: HEPES, EMEM, RPMI 1640 and human blood serum (HBS). (**a**) Measured spectra in black and the simulation curves in red. (**b**) Calculated component spectra. The measured spectra were simulated with 12% Cu(II)-estradiol-SC + 88% Cu(II)-oligomers in HEPES, 54% Cu(II)+EMEM + 46% Cu(II)-oligomers in EMEM, 20% Cu(II)+EMEM+ 80% Cu(II)-oligomers in RPMI 1640 and 100% Cu(II)-estradiol-SC in HBS.

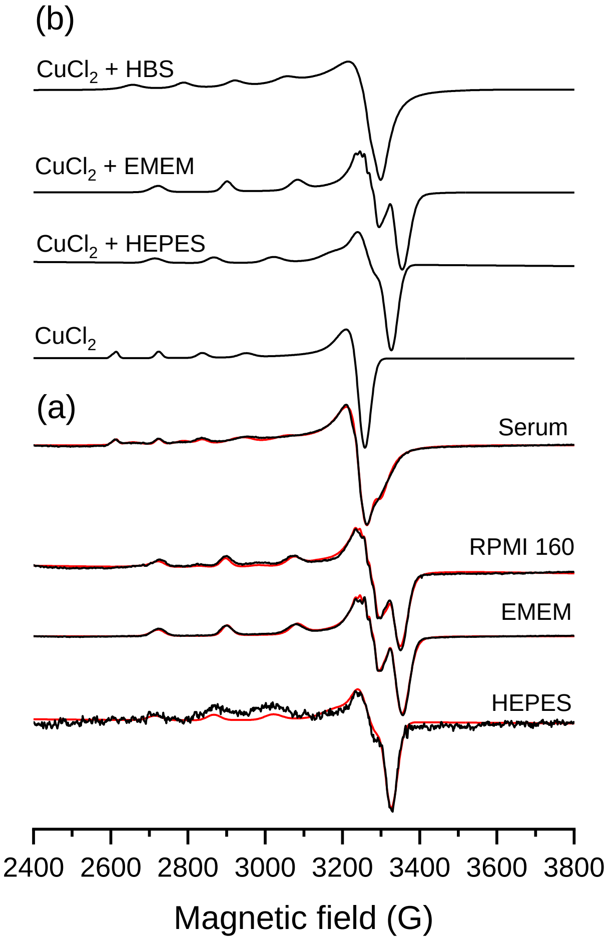

Figure S11. Frozen solution EPR spectra of the $CuCl_2$ dissolved in different biological media: HEPES, EMEM, RPMI 1640 and human blood serum (HBS). (**a**) Measured spectra in black and the simulation curves in red. (**b**) Calculated component spectra. The measured spectra were simulated with 100% Cu(II)+HEPES in HEPES, 100% Cu(II)+EMEM in EMEM, 70% Cu(II)+EMEM + 30% Cu(II) + HEPES in RPMI 1640 and 70% Cu(II) + HBS + 30% $CuCl_2$ in HBS.

Table S1. Anisotropic EPR parameters of components obtained by the simulation of frozen solution (77 K) EPR spectra recorded for $CuCl_2$ and Cu(II)-complexes of estradiol-SC, estradiol-TSC, Me-estradiol-TSC and Me_2-estradiol-TSC ligands dissolved in different biological media. The coupling values are in 10^{-4} cm^{-1} unit. The experimental errors were ±0.002 for g_x and g_y, ±0.001 for g_z, ±2 G for A_x and A_y, and ±1 G for A_z and nitrogen couplings.

	g_x	g_y	g_z	A_x	A_y	A_z	a_x^N	a_y^N	a_z^N	$g_{0,calc}$
$CuCl_2$	2.083	2.083	2.424	8.2	8.2	124.5				2.197
$CuCl_2$+HEPES	2.054	2.054	2.290	14.4	14.4	160.4				2.133
$CuCl_2$+EMEM	2.048	2.056	2.253	17.4	16.5	186.4	11.7	11.2	6.0	2.119
$CuCl_2$+HBS	2.065	2.065	2.361	16.5	16.5	142.3				2.164
Cu(II)-estradiol-SC	2.058	2.058	2.260	14.4	14.4	183.6				2.125
Cu(II)-estradiol-SC+HBS	2.062	2.062	2.257	34.7	34.7	163.9				2.127
Cu(II)-Me_2–estradiol-TSC	2.030	2.055	2.215	29.1	14.6	171.5	15	9.5	8.6	2.100
Cu(II)-Me_2–estradiol-TSC+EMEM	2.035	2.040	2.178	28.5	14.3	197.3	16.9	13.3	14.2	2.084
Cu(II)-Me_2–estradiol-TSC+HBS	2.030	2.055	2.166	17.9	38.4	192.1	17.1	9.6	15.2	2.084
Cu(II)-oligomers	2.10 [1]			20.0 [1]						

[1] Isotropic parameters.

Table S2. Crystal data and details of structure refinement.

	$[Cu(HL)Cl_2] \times H_2O \times 2CH_3OH$
Empirical formula	$C_{22} H_{38}Cl_2CuN_3O_6$
Formula weight	574.00
Temperature	103(2)
Radiation and wavelength	Mo-Kα, λ = 0.71073 Å
Crystal system	orthorhombic
Space group	*P* 21 21 21
Unit cell dimensions	*a* = 7.2028(3) Å
	b = 13.6795(6) Å
	c = 26.1919(12) Å
	α= 90°
	β= 90°
	γ= 90°
Volume	2580.7(2) $Å^3$
Z	4
Density (calculated)	1.480 Mg/m^3
Absorption coefficient, μ	1.095 mm^{-1}
F(000)	1208
Crystal colour	brown
Crystal description	needle
Crystal size	0.72 × 0.33 × 0.13 mm
Absorption correction	numerical
Max. and min. transmission	0.8931.000
θ–range for data collection	5.157≤θ ≤ 25.347°
Index ranges	-8 ≤*h* ≤ 8;-16≤*k* ≤16;-31 ≤ *l* ≤ 31
Reflections collected	71353
Completeness to 2θ	0.988
Absolute structure parameter	0.08(2)
Friedel coverage	0.746
Friedel fraction max.	0.996
Friedel fraction full	0.996
Independent reflections	4700 [*R*(int) =0.1398]
Reflections *I*>2σ (*I*)	4148
Refinement method	full-matrix least-squares on *F*2
Data / restraints / parameters	4700 /1 /318
Goodness-of-fit on *F*2	1.125
Final *R* indices [*I*>2σ (*I*)]	*R*1 =0.0503, *wR*2 =0.0780
R indices (all data)	*R*1 =0.0620, *wR*2 =0.0808
Max. and mean shift/esd	0.001;0.000
Largest diff. peak and hole	0.368;-0.345 e.$Å^{-3}$

Table S3. Bond lengths (Å) in $[Cu(HL)Cl_2]\times H_2O\times 2CH_3OH$.

Cu1-N1	1.966(4)	Cu1-O3	1.974(4)
Cu1-O2	2.032(3)	Cu1-Cl2	2.217(2)
Cu1-Cl1	2.470(1)	O3-C20	1.258(6)
O2-C3	1.377(6)	O1-C17	1.435(6)
N3-C20	1.322(7)	N2-C20	1.341(7)
N2-N1	1.379(6)	N1-C19	1.282(7)
C19-C2	1.445(7)	C2-C3	1.399(7)
C2-C1	1.403(7)	C1-C10	1.385(8)
C10-C5	1.404(7)	C10-C9	1.514(7)
C5-C4	1.407(7)	C5-C6	1.506(7)
C4-C3	1.378(8)	C6-C7	1.526(7)
C7-C8	1.512(7)	C8-C14	1.522(7)
C8-C9	1.546(7)	C9-C11	1.531(8)
C11-C12	1.535(8)	C12-C13	1.520(7)
C13-C18	1.524(7)	C13-C14	1.532(7)
C13-C17	1.537(7)	C14-C15	1.530(7)
C15-C16	1.531(8)	C16-C17	1.534(8)
O1M-C1M	1.400(8)	O2M-C2M	1.439(7)

Table S4. Bond angles (°) in $[Cu(HL)Cl_2]×H_2O×2CH_3OH$.

N1-Cu1-O3	81.0(2)	N1-Cu1-O2	87.1(2)
O3-Cu1-O2	160.6(1)	N1-Cu1-Cl2	157.3(2)
O3-Cu1-Cl2	93.3(1)	O2-Cu1-Cl2	91.7(1)
N1-Cu1-Cl1	97.3(1)	O3-Cu1-Cl1	101.0(1)
O2-Cu1-Cl1	95.7(1)	Cl2-Cu1-Cl1	105.38(6)
C20-O3-Cu1	113.1(3)	C3-O2-Cu1	128.9(3)
C20-N2-N1	115.5(4)	C19-N1-N2	118.4(4)
C19-N1-Cu1	130.9(4)	N2-N1-Cu1	110.6(3)
O3-C20-N3	122.9(5)	O3-C20-N2	119.5(5)
N3-C20-N2	117.5(5)	N1-C19-C2	124.1(5)
C3-C2-C1	117.9(5)	C3-C2-C19	126.0(5)
C1-C2-C19	116.1(5)	C10-C1-C2	123.3(5)
C1-C10-C5	117.4(5)	C1-C10-C9	121.1(5)
C5-C10-C9	121.6(5)	C10-C5-C4	120.0(5)
C10-C5-C6	122.2(5)	C4-C5-C6	117.8(5)
C3-C4-C5	121.1(5)	O2-C3-C4	120.9(5)
O2-C3-C2	119.2(5)	C4-C3-C2	119.9(5)
C5-C6-C7	113.9(5)	C8-C7-C6	111.7(5)
C7-C8-C14	113.1(4)	C7-C8-C9	109.3(4)
C14-C8-C9	108.6(4)	C10-C9-C11	114.0(4)
C10-C9-C8	110.2(4)	C11-C9-C8	111.5(4)
C9-C11-C12	112.8(5)	C13-C12-C11	111.9(5)
C12-C13-C18	111.3(5)	C12-C13-C14	108.2(4)
C18-C13-C14	113.9(4)	C12-C13-C17	115.8(5)
C18-C13-C17	109.1(4)	C14-C13-C17	98.0(4)
C8-C14-C15	118.8(5)	C8-C14-C13	112.6(4)
C15-C14-C13	104.7(4)	C14-C15-C16	102.6(4)
C15-C16-C17	106.6(4)	O1-C17-C16	108.8(5)
O1-C17-C13	116.4(5)	C16-C17-C13	105.0(4)

Table S5. Analysis of potential hydrogen bonds and schemes with d(D...A) < R(D)+R(A)+0.50, d(H...A) < R(H)+R(A)-0.12 Ang., D-H...A > 100.0 Deg.

Nr	Res Donor --- H....Acceptor	Symm. op.	H...A (Å)	D...A (Å)	D - H...A (°)
1	1 N3 --H3B ..Cl2	-1-x,1/2+y,1/2-z	2.51	3.331(4)	156
2	1 N3 --H3A ..Cl1	-1+x,y,z	2.45	3.301(4)	162
3	2 O1M --H1M ..O1	3/2-x,1-y,-1/2+z	1.94	2.750(6)	172
4	1 O2 --H2 ..O2M	1-x,-1/2+y,1/2-z	1.69(5)	2.611(6)	165(5)
5	1 N2 --H2A ..O1W	-1+x.y.z	1.83	2.685(6)	175
6	3 O2M --H2M ..O1	3/2-x.1-y.-1/2+z	2.01	2.791(6)	159
7	1 O1 --H1 ..O1M	1-x.1/2+y.1/2-z	1.81	2.617(6)	167
8	4 O1W --H1WA ..Cl2	-x.1/2+y.1/2-z	2.77	3.385(5)	130
9	4 O1W --H1WA ..O3	-x.1/2+y.1/2-z	2.12	2.898(5)	152
10	4 O1W --H1WB ..Cl1	1-x.1/2+y.1/2-z	2.23	3.146(5)	158
11	3 C2M --H2MC ..Cl2	1-x.1/2+y.1/2-z	2.76	3.502(8)	133

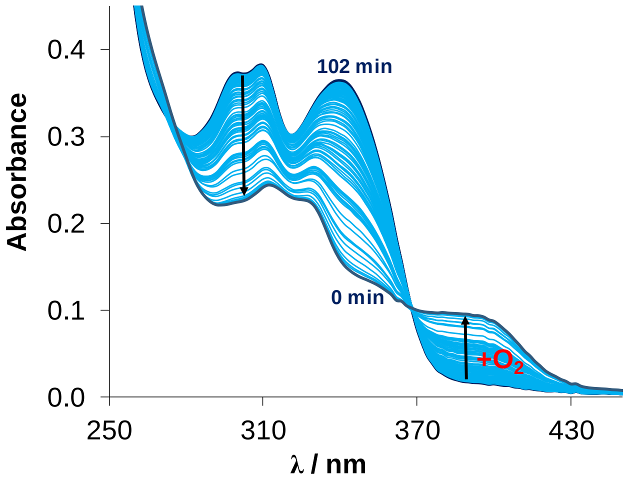

Figure S12. Time dependence of the effect of bubbling O_2 through the sample following the reaction with 50 equiv. GSH (1.25 mM) and Cu(II)-Me-estradiol-TSC complex (25 μM) at pH 7.4 in 30% (*v*/*v*) DMSO/H_2O. (T = 25 °C; I = 0.1 M (KCl); ℓ = 1 cm).

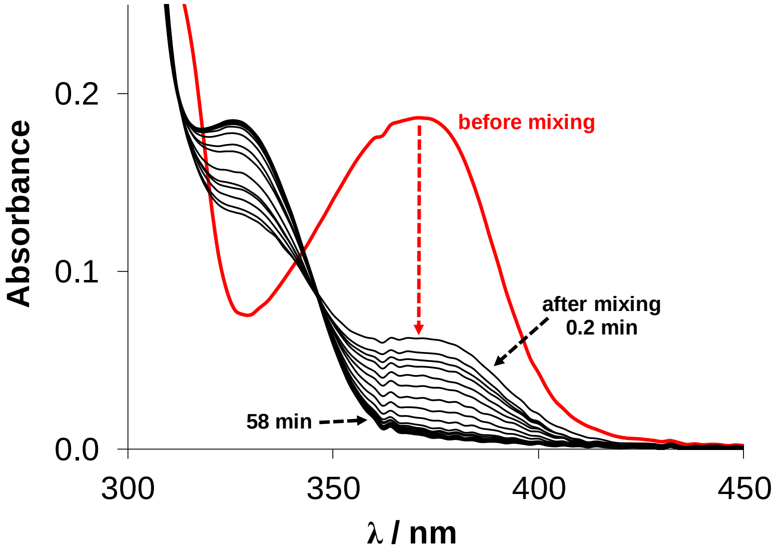

Figure S13. Time-dependent changes of the UV–vis spectra recorded for the Cu(II)– estradiol-SC complex (25 μM) in the presence of 50 equiv. ascorbic acid (1.25 mM) at pH 7.4 in 30% (*v*/*v*) DMSO/H_2O under anaerobic conditions. (T = 25 °C; I = 0.1 M (KCl); ℓ = 1 cm).

Treatment of components prior in vitro DNA cleavage assay

The components of the reaction buffer (100 mM HEPES at various pH, 50% (v/v) DMSO, sterile distilled H_2O) were treated with 2.5 mg/dm^3 Chelex-100 (Sigma-Aldrich) cation exchange resin for 30 min at 25 °C in order to remove trace metals contamination. In case of DMSO-free solutions this was followed by filtration through 0.2 μm pore size sterile, non-pyrogenic, endotoxin-free, non-cytotoxic PES filter

(Sarstedt) under laminal flow. In case of DMSO-containing solutions hydrophilic polytetrafluoroethylene membrane (PTFE) filters were used. 5 mM ascorbic acid and GSH solutions (dissolved in H_2O) were also treated in the same way and stored at –30 °C until further use. Ligands were dissolved in 100 % (v/v) DMSO solution to get 5 mM stock solutions. Portion of the stock solutions were further diluted 100 times with 5 mM HEPES 50% (v/v) DMSO pH 6.5 solution, then Chelex-100 treatment and filtration was applied as described above. These steps did not affect the UV–vis spectra of the ligands. The diluted ligand solutions were stored in 500 μL portions at –80 °C until further use. pUC119ΔH+N-N6 plasmid DNA was purified by NucleoBond Xtra Midi kit (Macherey-Nagel) and was treated with Chelex-100 resin and filtered as described above. This step was followed by buffer exchange of the DNA sample using Amicon 10K 0.5 mL filters (4 × 5 min, 14000 g, 25 °C) with Chelex-100 treated 10 mM HEPES at pH 7.4. The ratio of UV absorbance at 260 and 280 nm was 1.87 indicating that the DNA was free of proteins and RNA. No genomic DNA contamination was detected by gel electrophoresis and the plasmid DNA was in superhelical form with only a small fraction of the open circular form. Furthermore, Chelex-100 treatment and buffer exchange did not increase the circular to superhelical ratio.

In vitro DNA cleavage assay detailed results

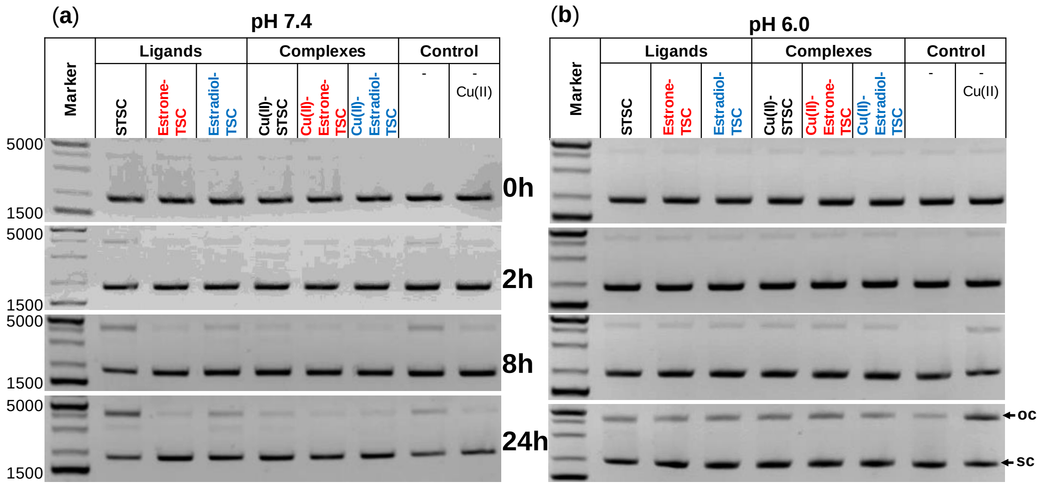

Figure S14. Nuclease activity of TSC derivatives and their Cu(II)-complexes analyzed by 1% (*w/v*) agarose gel electrophoresis. The reaction mixtures contained 10 ng/μL pUC119ΔH+N-N6 plasmid (100 ng total); ~20 μM ligands (in the presence or absence of 18 μM $CuCl_2$), 10 mM HEPES and were incubated up to 24 h at 37 °C. (**a**) pH = 7.4; (**b**) pH = 6.0. Arrows indicate various forms of the plasmid: oc -open circular, lin - linear, sc - supercoiled. Control reactions were set up with DNA or DNA in the presence of 18 μM $CuCl_2$. 1 kb Gene Ruler Plus served as DNA marker.

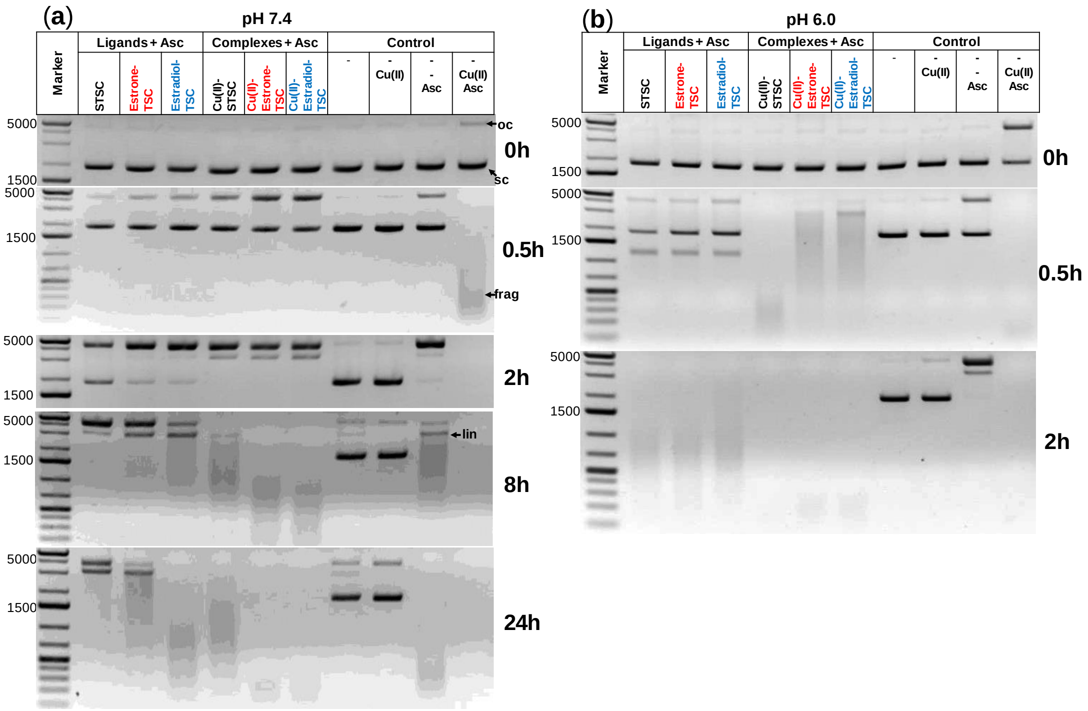

Figure S15. Nuclease activity of TSC derivatives and their Cu(II)-complexes analyzed by 1% (*w*/*v*) agarose gel electrophoresis. The reaction mixtures contained 10 ng/μL pUC119ΔH+N-N6 plasmid (100 ng total); ~20 μM ligands (in the presence or absence of 18 μM $CuCl_2$), 30% DMSO / 70% 10 mM HEPES and were incubated up to 24 h at 37 °C. (**a**) pH = 7.4; (**b**) pH = 6.0. Asc indicates the presence of 1 mM ascorbic acid in the reaction mixture. Arrows indicate various forms of the plasmid: oc - open circular, lin - linear, sc - supercoiled, frag - fragmented. Control reactions were set up with DNA or DNA in the presence of 18 μM $CuCl_2$. DNA in the presence of 1 mM ascorbic acid, DNA in the presence of both 1 mM ascorbic acid and 18 μM $CuCl_2$. 1 kb Gene Ruler Plus served as DNA marker.

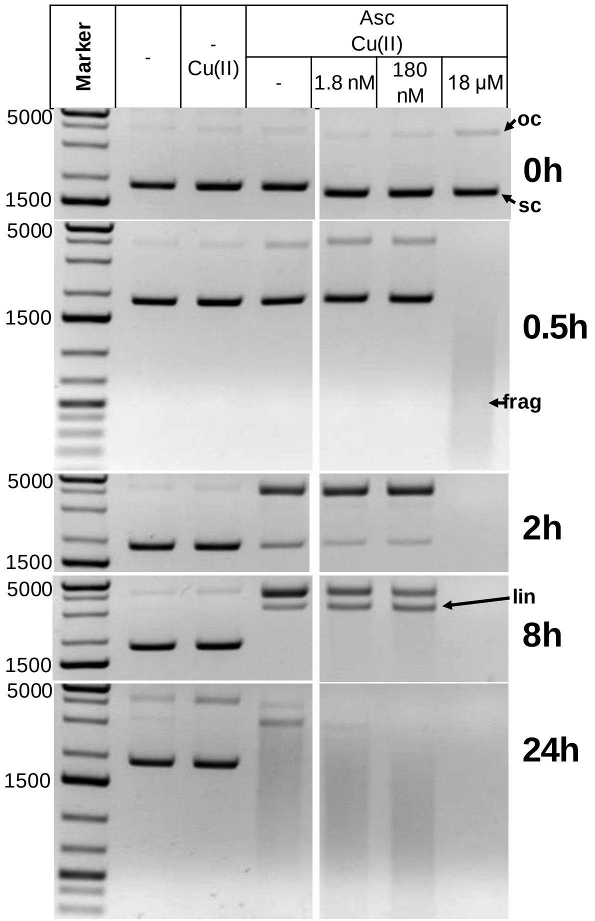

Figure S16. Nuclease activity of 1 mM ascorbic acid with increasing amounts of Cu(II) analyzed by 1% (*w/v*) agarose gel electrophoresis. The reaction mixtures contained 10 ng/μL pUC119ΔH+N-N6 plasmid (100 ng total) in the presence or absence of up to 18 μM $CuCl_2$, 30% DMSO / 70% 10 mM HEPES; pH = 7.4 and were incubated up to 24 h at 37 °C. Asc indicates the presence of 1 mM ascorbic acid in the reaction mixture. Arrows indicate various forms of the plasmid: oc - open circular, lin - linear, sc - supercoiled, frag - fragmented. Control reactions contained only DNA or DNA in the presence of 18 μM $CuCl_2$. 1 kb Gene Ruler Plus served as DNA marker.

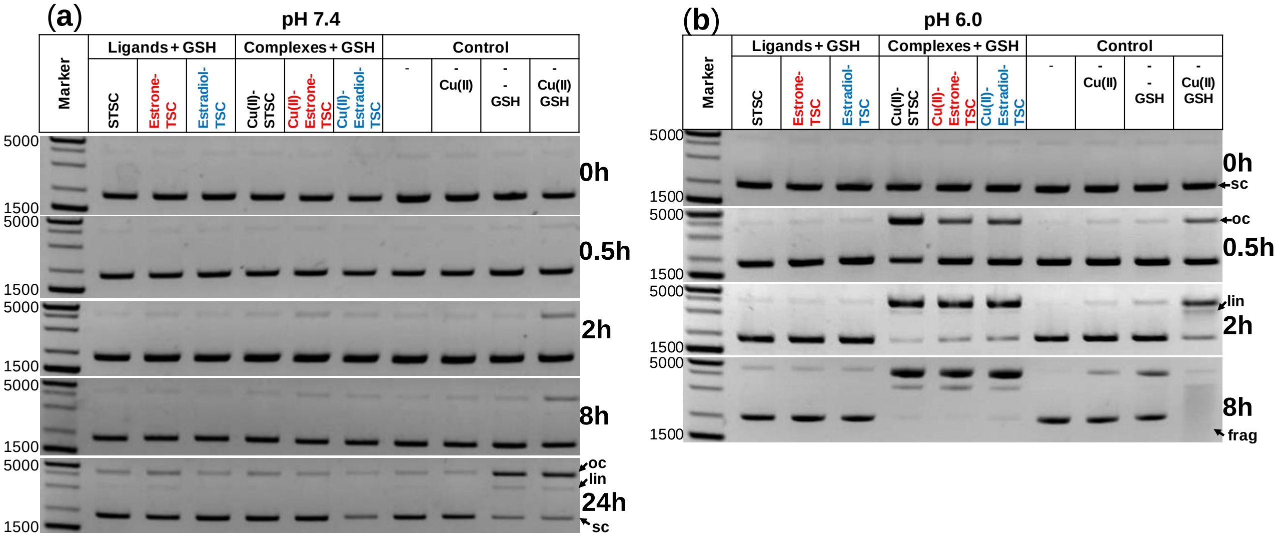

Figure S17. Nuclease activity of TSC derivatives and their Cu(II)-complexes analyzed by 1% (*w*/*v*) agarose gel electrophoresis. The reaction mixtures contained 10 ng/µL pUC119ΔH+N-N6 plasmid (100 ng total); ~20 µM ligands (in the presence or absence of 18 µM $CuCl_2$), 30% DMSO / 70% 10 mM HEPES and incubated up to 24 h at 37 °C. (**a**) pH = 7.4; (**b**) pH = 6.0. GSH indicated the presence of 1 mM GSH in the reaction mixture. Arrows indicate various forms of the plasmid: oc - open circular, lin - linear, sc - supercoiled, frag - fragmented. Control reactions were set up with only DNA or DNA in the presence of 18 µM $CuCl_2$, DNA in the presence of 1 mM GSH, DNA in the presence of both 1 mM GSH and 18 µM $CuCl_2$. 1 kb Gene Ruler Plus served as DNA marker.

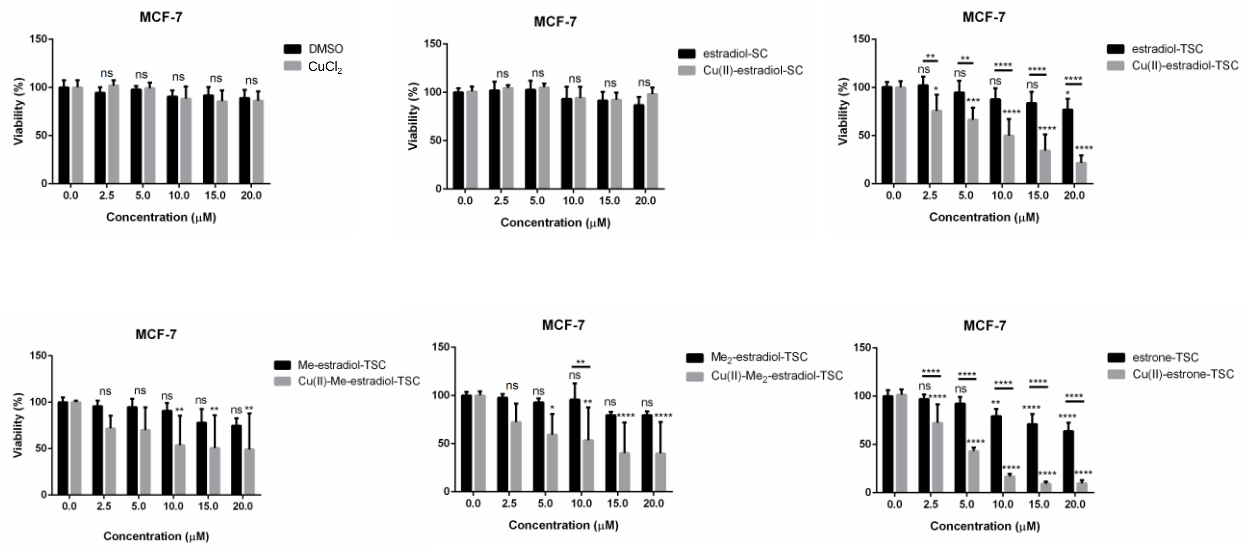

Figure S18. Viability (%) measured on MCF-7 cells upon the treatment with estradiol-SC, estradiol-TSC, Me-estradiol-TSC, Me_2-estradiol-TSC, estrone-TSC and their Cu(II) complexes at various concentrations (2.5, 5.0, 10.0, 15.0, 20.0 µM) in addition to the DMSO blank (90% DMSO-10% PBS buffer) and $CuCl_2$ as indicated in the figure using 24 h incubation time.

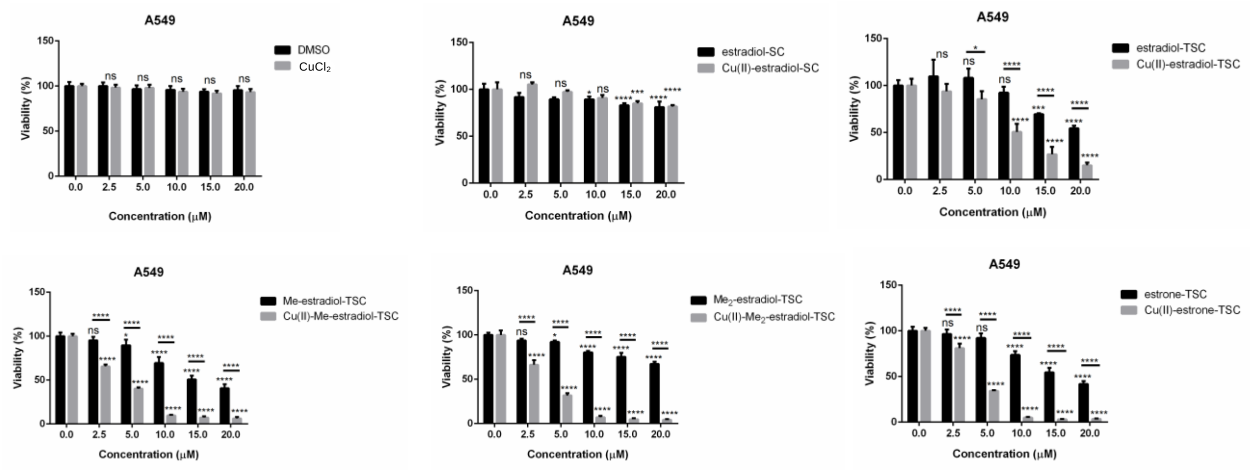

Figure S19. Viability (%) measured on A549 cells upon the treatment with estradiol-SC, estradiol-TSC, Me-estradiol-TSC, Me$_2$-estradiol-TSC, estrone-TSC and their Cu(II) complexes at various concentrations (2.5, 5.0, 10.0, 15.0, 20.0 μM) in addition to the DMSO blank (90% DMSO-10% PBS buffer) and $CuCl_2$ as indicated in the figure using 24 h incubation time.

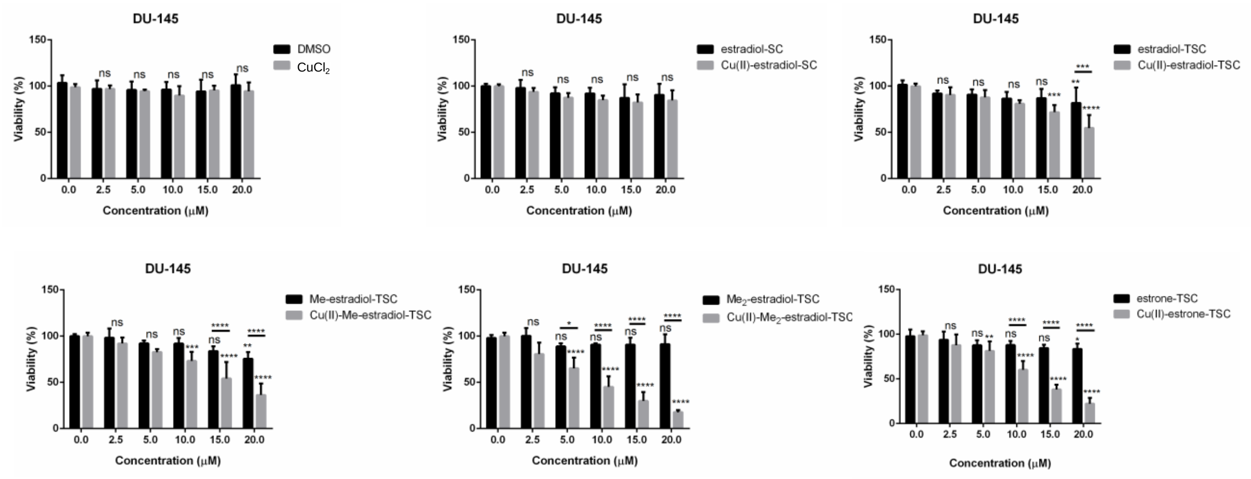

$CuCl_2$

Figure S20. Viability (%) measured on DU-145 cells upon the treatment with estradiol-SC, estradiol-TSC, Me-estradiol-TSC, Me$_2$-estradiol-TSC, estrone-TSC and their Cu(II) complexes at various concentrations (2.5, 5.0, 10.0, 15.0, 20.0 μM) in addition to the DMSO blank (90% DMSO-10% PBS buffer) and $CuCl_2$ as indicated in the figure using 24 h incubation time.

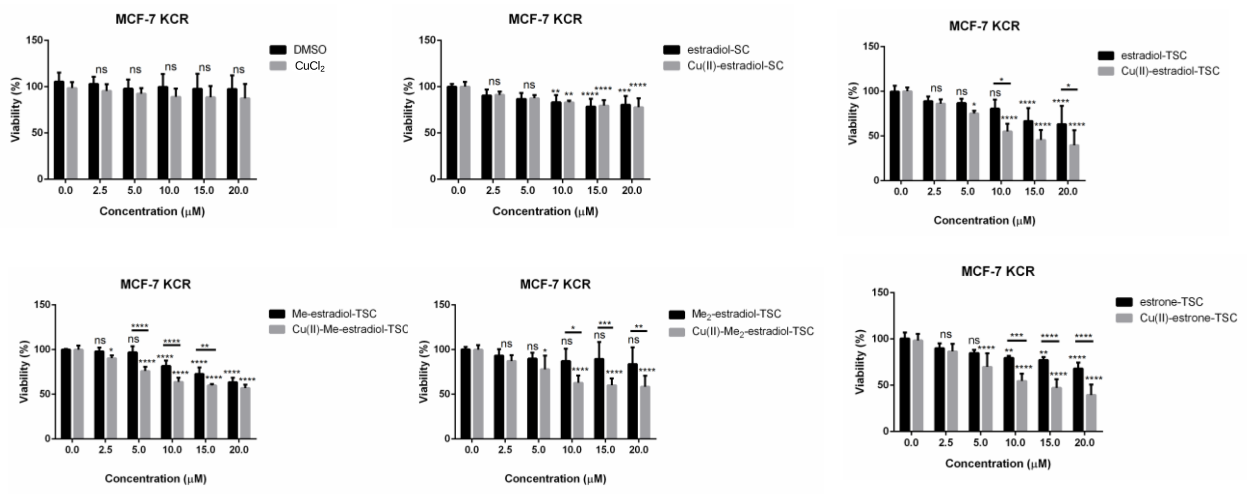

Figure S21. Viability (%) measured on MCF-7-KCR cells upon the treatment with estradiol-SC, estradiol-TSC, Me-estradiol-TSC, Me2-estradiol-TSC, estrone-TSC and their Cu(II) complexes at various concentrations (2.5, 5.0, 10.0, 15.0, 20.0 μM) in addition to the DMSO blank (90% DMSO-10% PBS buffer) and $CuCl_2$ as indicated in the figure using 24 h incubation time.

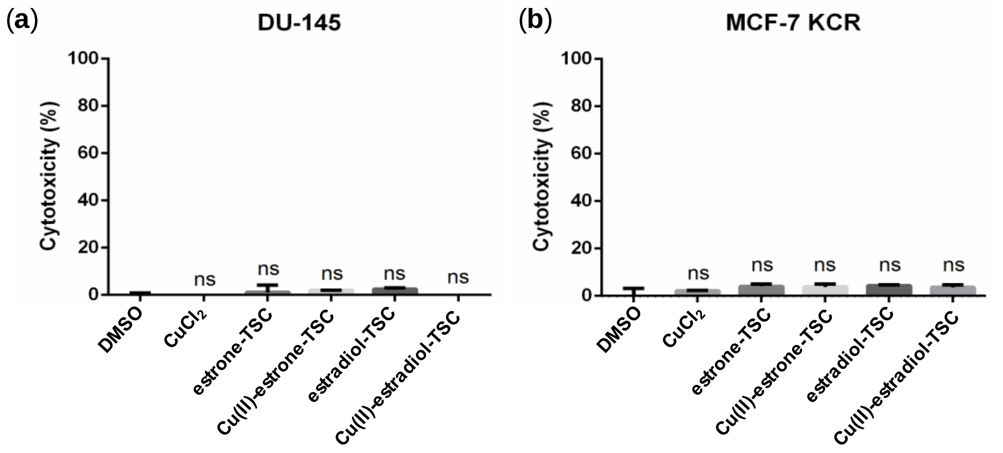

Figure S22. LDH assay to measure the reduction in plasma membrane integrity: cytotoxicity (%) obtained on (**a**) DU-145 and (**b**) MCF-7 KCR cells upon the treatment with estrone-TSC and estradiol-TSC and their Cu(II) complexes (20 μM) in addition to the DMSO blank (90% DMSO-10% PBS buffer) and $CuCl_2$ (20 μM) as indicated in the figure using 24 h incubation time.

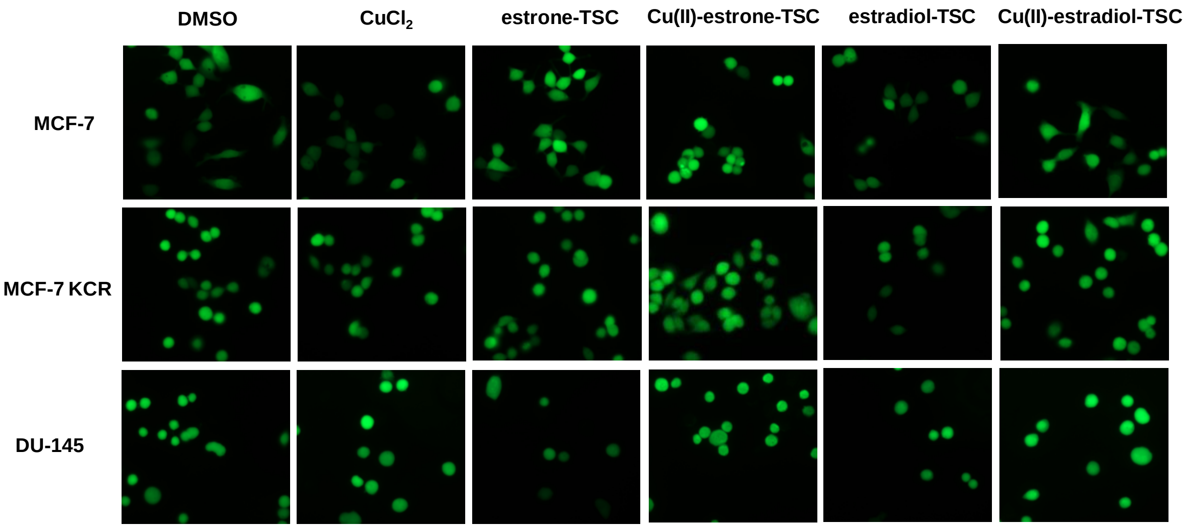

Figure S23. Fluorescence microscopic images show the DCFDA staining to determine ROS levels in MCF-7, MCF-7 KCR and DU-154 cells upon the treatment with estrone-TSC and estradiol-TSC and their Cu(II) complexes (20 μM) in addition to the DMSO blank (90% DMSO-10% PBS buffer) and $CuCl_2$ (20 μM) as indicated in the figure using 6 h incubation time.

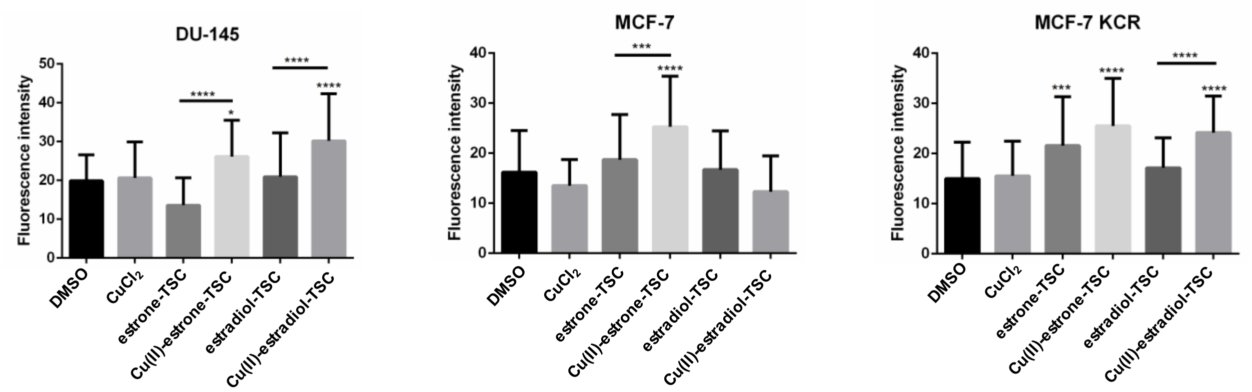

Figure S24. Level of ROS generation measured by DCFDA staining on MCF-7, MCF-7 KCR and DU-154 cells upon the treatment with estrone-TSC and estradiol-TSC and their Cu(II) complexes (20 μM) in addition to the DMSO blank (90% DMSO-10% PBS buffer) and $CuCl_2$ (20 μM) as indicated in the figure using 6 h incubation time.

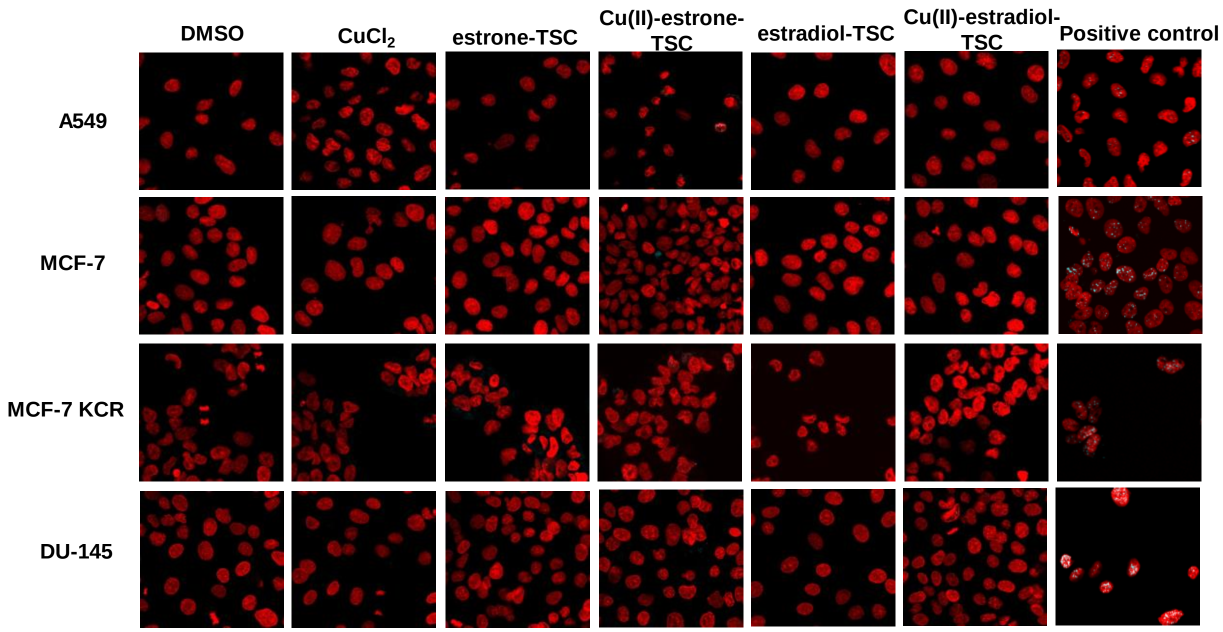

Figure S25. Fluorescence microscopic images show the γH2AX immunostaining to determine the degree of DNA damage in A549, MCF-7, MCF-7 KCR and DU-154 cells upon the treatment with estrone-TSC and estradiol-TSC and their Cu(II) complexes (20 μM) in addition to the DMSO blank (90% DMSO-10% PBS buffer) and $CuCl_2$ (20 μM) as indicated in the figure using 3 h incubation time. In the case of the positive control cells the DNA damage was induced directly by ionizing radiation (exposure time: 1 min; dose: 2 Gy).

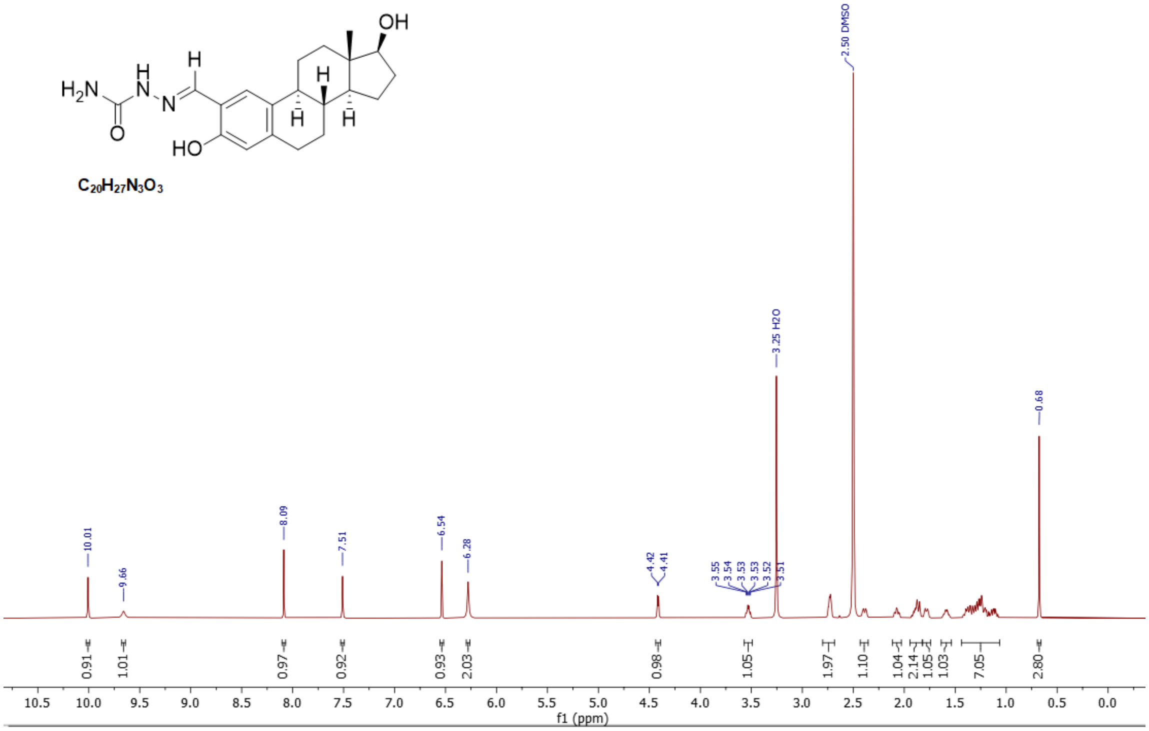
C20H27N3O3
H2N
H
N
N
H
O
HO
OH
2.50 DMSO
3.25 H2O
0.68
10.01
9.66
8.09
7.51
6.54
6.28
4.42
4.41
3.55
3.54
3.53
3.53
3.52
3.51
0.91
1.01
0.97
0.92
0.93
2.03
0.98
1.05
1.97
1.10
1.04
2.14
1.05
1.03
7.05
2.80
f1 (ppm)

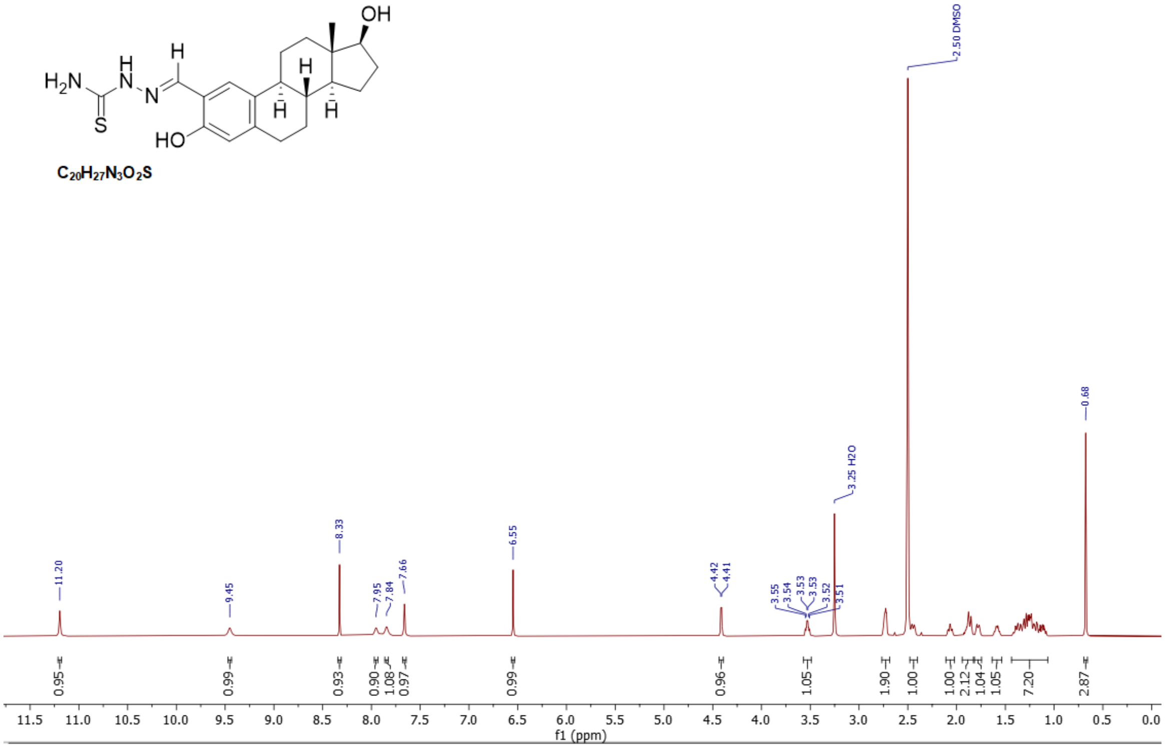
C20H27N3O2S
H2N
H
N
N
H
S
HO
OH
2.50 DMSO
3.25 H2O
0.68
11.20
9.45
8.33
7.95
7.84
7.66
6.55
4.42
4.41
3.55
3.54
3.53
3.53
3.52
3.51
0.95
0.99
0.93
0.90
1.08
0.97
0.99
0.96
1.05
1.90
1.00
1.00
2.12
1.04
1.05
7.20
2.87
f1 (ppm)

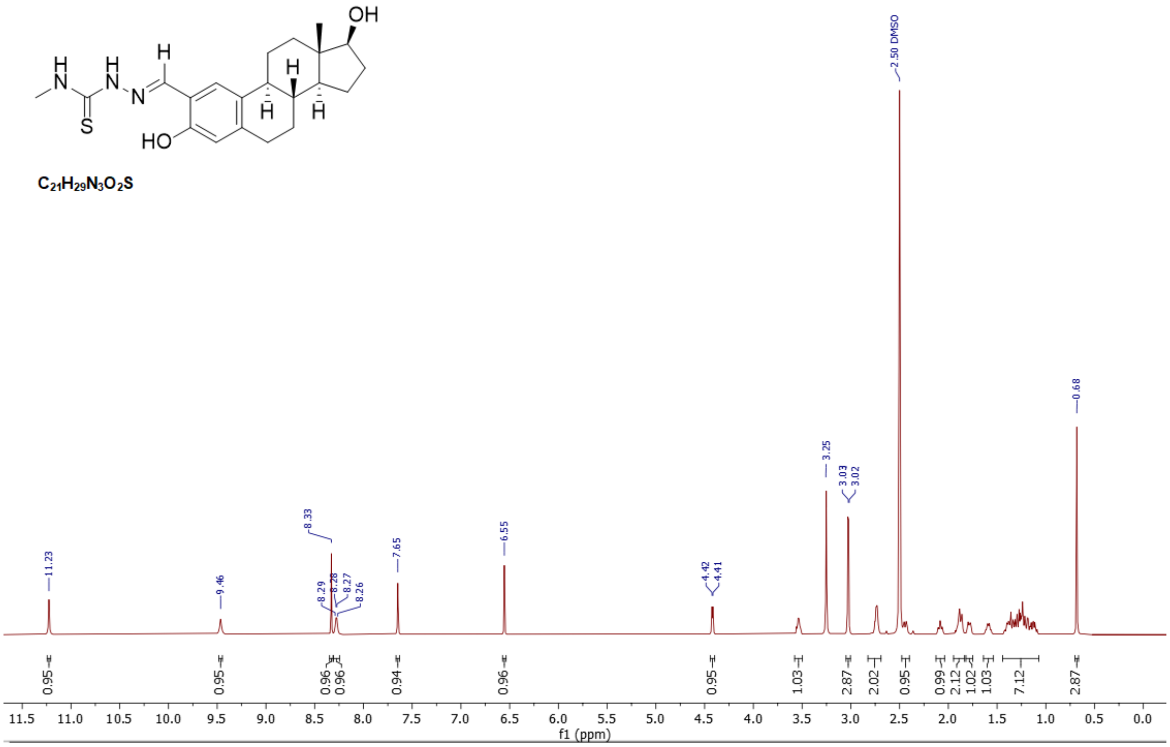

$C_{21}H_{29}N_3O_2S$

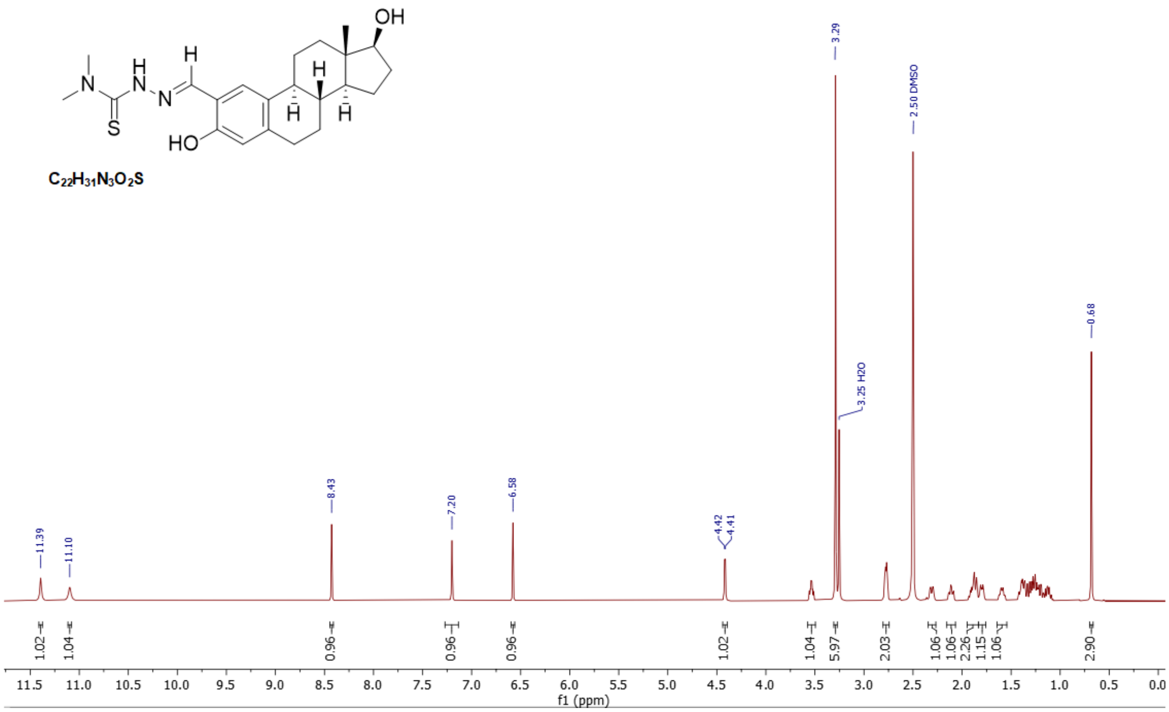

$C_{22}H_{31}N_3O_2S$

Figure S26. ^{1}HNMR spectra of the indicated (thio)semicarbazones in DMSO-d_6.

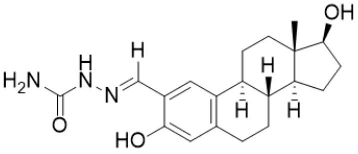

$C_{20}H_{27}N_3O_3$

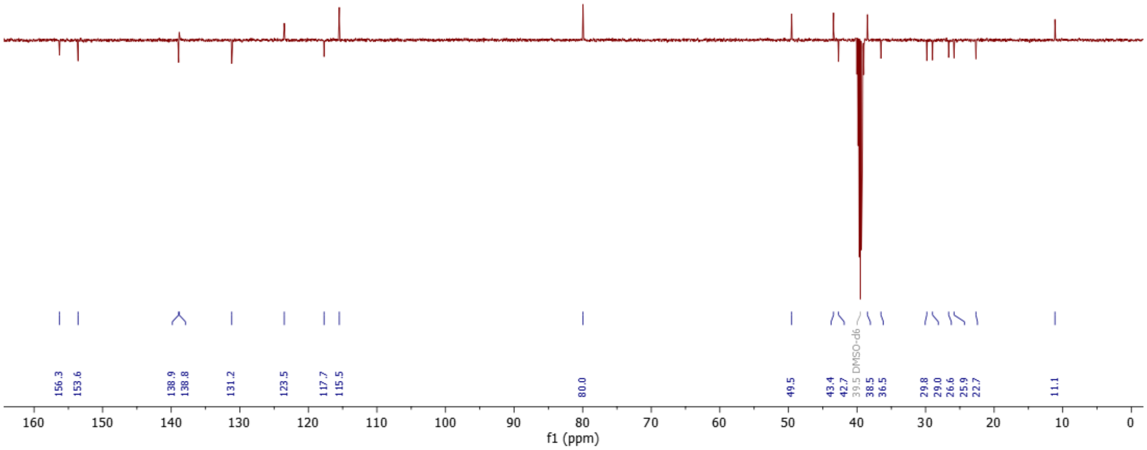

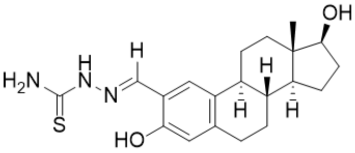

$C_{20}H_{27}N_3O_2S$

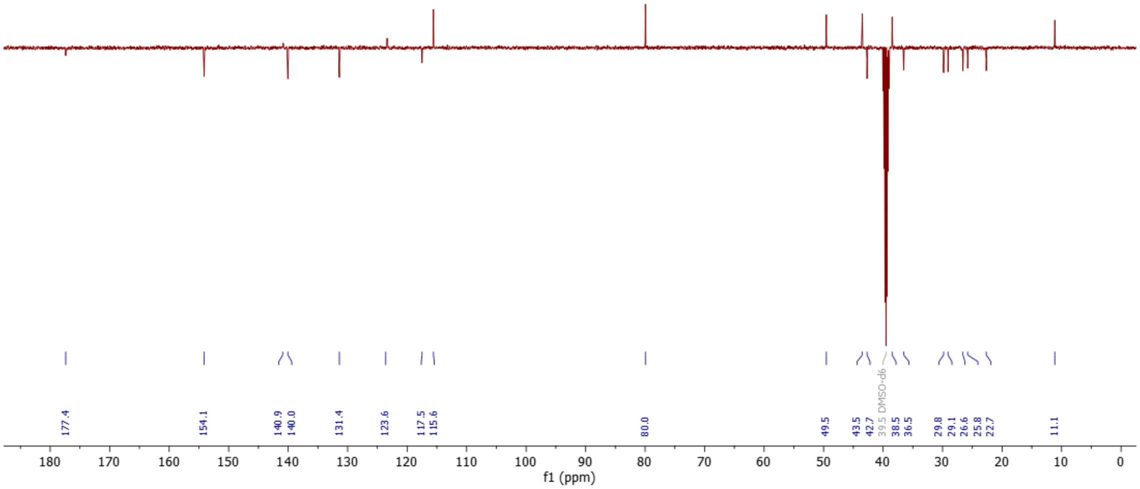

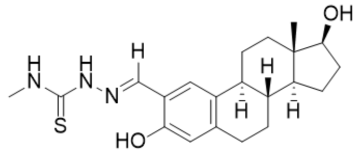

$C_{21}H_{29}N_3O_2S$

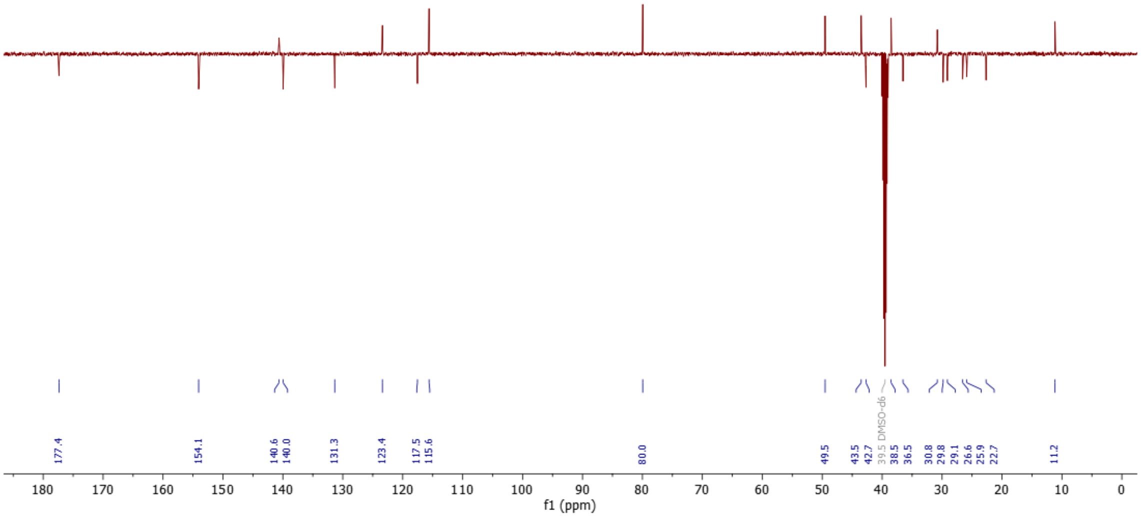

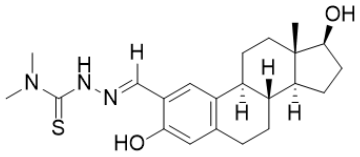

$C_{22}H_{31}N_3O_2S$

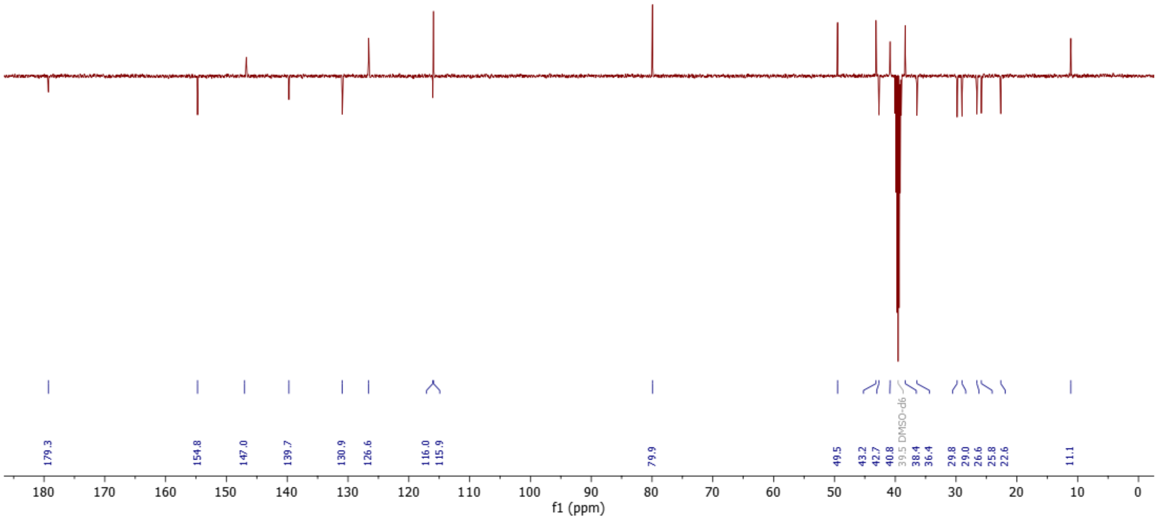

Figure. S27. ^{13}C NMR spectra of the indicated (thio)semicarbazones in DMSO-d_6.

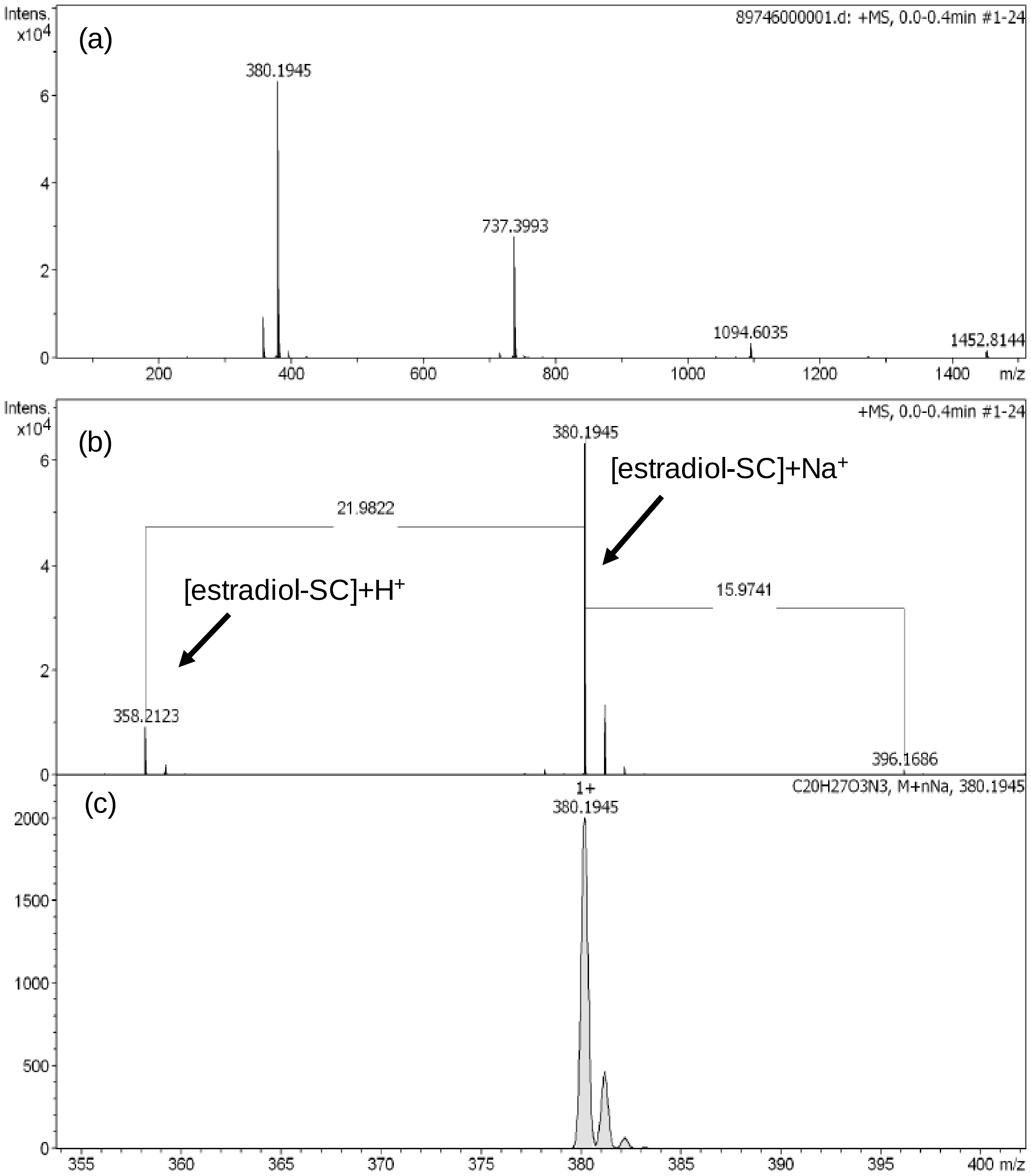
Intens.
x10^4
(a)
89746000001.d: +MS, 0.0-0.4min #1-24
380.1945
737.3993
1094.6035
1452.8144
m/z
(b)
+MS, 0.0-0.4min #1-24
380.1945
[estradiol-SC]+Na+
21.9822
[estradiol-SC]+H+
15.9741
358.2123
396.1686
(c)
C20H27O3N3, M+nNa, 380.1945
1+
380.1945
m/z

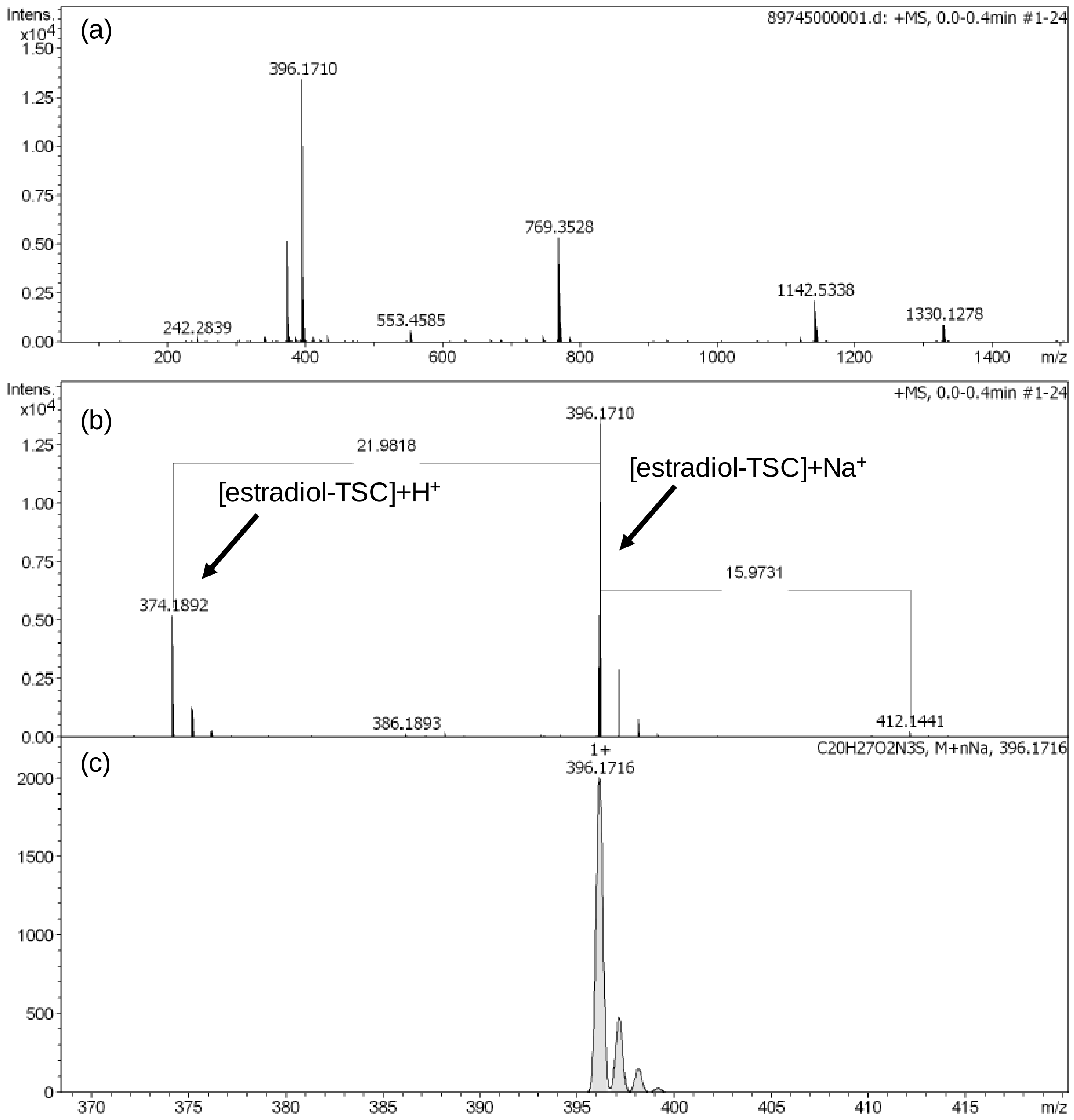
(a)
89745000001.d: +MS, 0.0-0.4min #1-24
396.1710
769.3528
1142.5338
1330.1278
553.4585
242.2839
(b)
+MS, 0.0-0.4min #1-24
396.1710
21.9818
[estradiol-TSC]+Na+
[estradiol-TSC]+H+
15.9731
374.1892
386.1893
412.1441
(c)
C20H27O2N3S, M+nNa, 396.1716
1+
396.1716
Intens.
x10^4
m/z

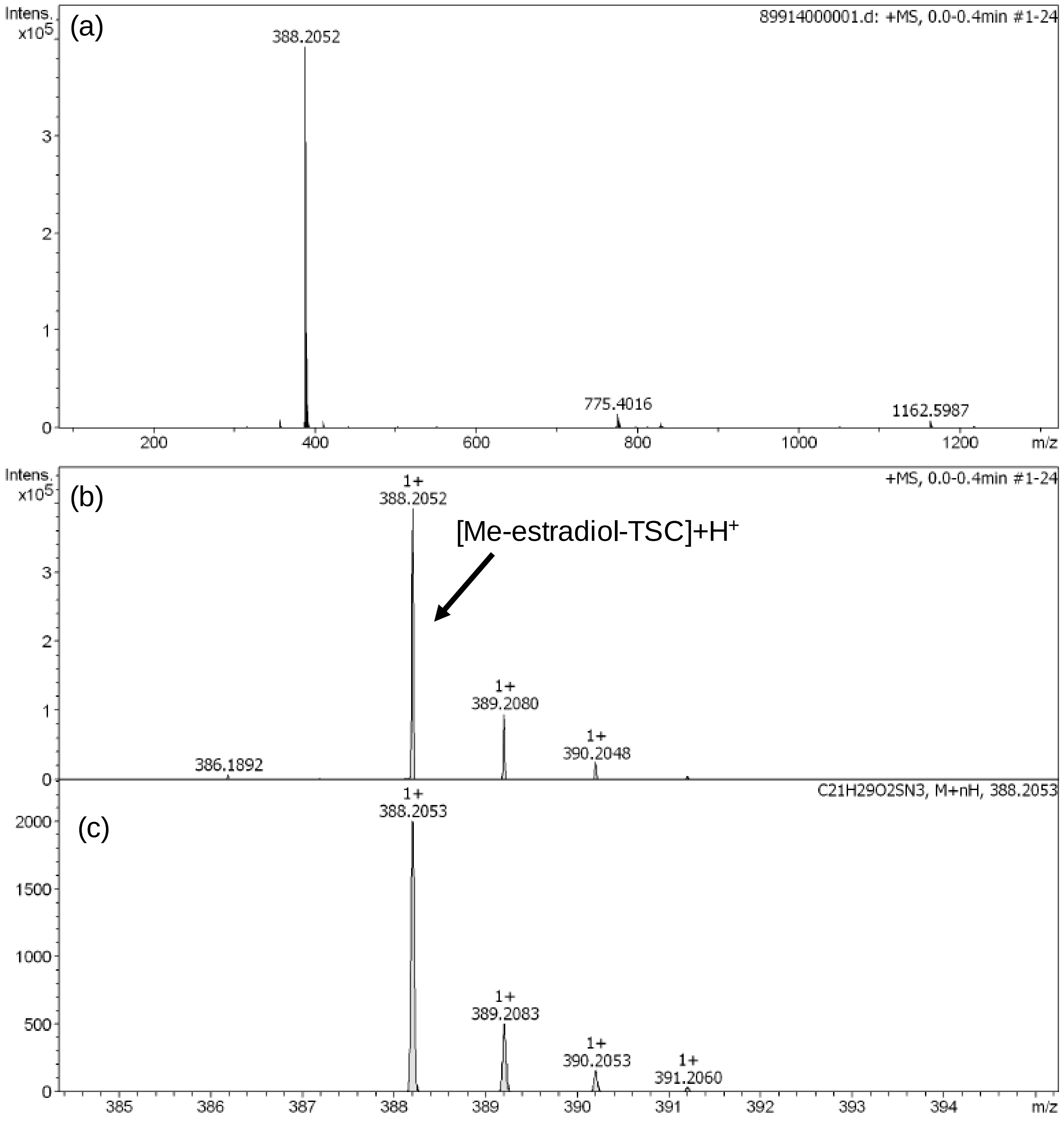

Intens.
x10^5
(a)
89914000001.d: +MS, 0.0-0.4min #1-24
388.2052
775.4016
1162.5987
m/z
(b)
+MS, 0.0-0.4min #1-24
1+
388.2052
[Me-estradiol-TSC]+H+
1+
389.2080
1+
390.2048
386.1892
(c)
C21H29O2SN3, M+nH, 388.2053
1+
388.2053
1+
389.2083
1+
390.2053
1+
391.2060
m/z

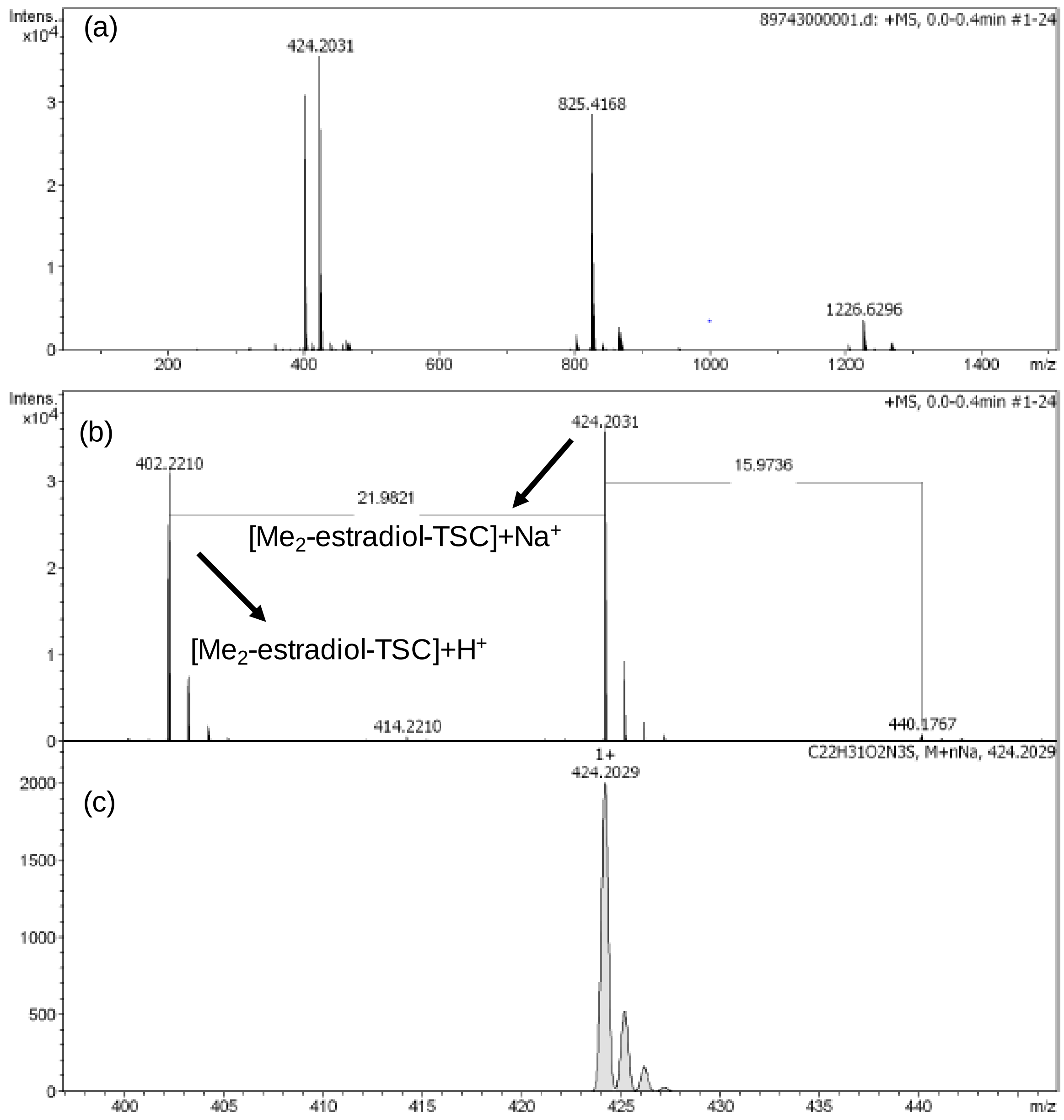

Figure S28. ESI-MS spectra of the indicated novel (thio)semicarbazones. (**a**) Measured, (**b**) zoomed range, (**c**) simulated MS spectra. Samples were prepared in methanol.
